# *Down syndrome cell adhesion molecule 1*: testing for a role in insect immunity, behaviour and reproduction

**DOI:** 10.1098/rsos.160138

**Published:** 2016-04-20

**Authors:** Robert Peuß, Kristina U. Wensing, Luisa Woestmann, Hendrik Eggert, Barbara Milutinović, Marlene G. U. Sroka, Jörn P. Scharsack, Joachim Kurtz, Sophie A. O. Armitage

**Affiliations:** Institute for Evolution and Biodiversity, University of Münster, Hüfferstrasse 1,48149 Münster, Germany

**Keywords:** *Dscam1*, *Drosophila melanogaster*, fecundity, immune defence, locomotion, *Tribolium castaneum*

## Abstract

*Down syndrome cell adhesion molecule 1* (*Dscam1*) has wide-reaching and vital neuronal functions although the role it plays in insect and crustacean immunity is less well understood. In this study, we combine different approaches to understand the roles that *Dscam1* plays in fitness-related contexts in two model insect species. Contrary to our expectations, we found no short-term modulation of *Dscam1* gene expression after haemocoelic or oral bacterial exposure in *Tribolium castaneum*, or after haemocoelic bacterial exposure in *Drosophila melanogaster.* Furthermore, RNAi-mediated *Dscam1* knockdown and subsequent bacterial exposure did not reduce *T. castaneum* survival. However, *Dscam1* knockdown in larvae resulted in adult locomotion defects, as well as dramatically reduced fecundity in males and females. We suggest that *Dscam1* does not always play a straightforward role in immunity, but strongly influences behaviour and fecundity. This study takes a step towards understanding more about the role of this intriguing gene from different phenotypic perspectives.

## 1. Introduction

Dscam1 is an immunoglobulin-containing cell adhesion molecule with functions in diverse processes such as neural wiring and the immune system [1–4]. In addition to this duality, *Dscam1* has received attention, particularly in arthropods, because of the remarkable intra-individual mRNA diversity that it can produce via a combination of germline exon variability [1,5] and mutually exclusive alternative splicing of these exons [1,6]. In brief, in Pancrustacea (insects and crustaceans), multiple exon duplications have evolved within three exon clusters, exons 4, 6 and 9 in *Drosophila melanogaster*, which encode parts of the extracellular domain of the protein (electronic supplementary material, figure S1). Although the fact that one molecule has evolved a function in diverse biological processes is not in itself unusual (e.g. MHC class I [7]), the diverse potential functions of one molecule are not frequently examined in parallel. Therefore, in this study, we have combined different approaches to try to understand more about *Dscam1* in several situations that are relevant to fitness: immunity, behaviour and reproduction.

Dscam1 was initially found to be a vital cell surface receptor in the nervous system of *D. melanogaster* [1]. It plays an essential role in self- and non-self-recognition, which leads to neurite self-avoidance. Dscam1 isoform diversity underlies its function as a molecular surface code, such that when sister neurites express identical isoforms, homophilic recognition occurs between these isoforms and the two neurites are repulsed from one another (for a review, see [8]). However, the expression of *Dscam1* is not restricted to the nervous system. A number of studies have shown that several pancrustacean tissues express *Dscam1* [2,9–12], including the two main tissues responsible for immune defence: the fat body and haemocytes [2,10,13–15]. The fat body is a major insect biosynthetic and storage organ also producing antimicrobial peptides [16,17], and haemocytes are the insect and crustacean immune cells ([18,19], respectively). As models in this study, we used *D. melanogaster* and the red flour beetle *Tribolium castaneum*. To the best of our knowledge, there have been only two studies [20,21] examining *Dscam1* in *D. melanogaster* in relation to immunity since Watson *et al*.'s [2] original discovery, and no representatives of the most speciose order of insect, Coleoptera, have been studied, even though the alternatively spliced *Dscam1* exons have been described for *T. castaneum* [2]. Therefore, in order to establish whether *T. castaneum* shows similar gene expression patterns to *D. melanogaster*, our first objective was to test *Dscam1* expression across life-history stages and tissues (including immune tissues). By using these two distantly related holometabolous insect species, we hoped to be able to make robust interpretations of our findings.

Dscam1 appears to play a role in the immune defence of a number of pancrustaceans (for reviews, see [22–24]). The knockdown of *Dscam1* can reduce phagocytosis [2,4] and recombinantly expressed Dscam1 has been found to bind to bacteria [2,10,15]. The hypothesized role of isoform diversity in Dscam1 in immunity is that it might provide potential receptor, opsonin or effector diversity for recognizing or reacting with some degree of specificity towards diverse pathogen and parasite antigens [2,4,10,25–28], although how this might function mechanistically is unclear [29]. Evidence from mosquitoes shows that expression of *Dscam1* exon 4 variants can relate to exposure to different pathogens. However, a strong and distinct association between the expression of *Dscam1* splice variants after exposure to pathogens was not found in *D. melanogaster* [20,21] or *Daphnia magna* [21].

Furthermore, and of direct relevance to this study, mixed results have been obtained with respect to overall *Dscam1* transcript expression after pathogen exposure: in the majority of cases, upregulation has been found at some time point after exposure [10–12,14,30], but there are also cases where downregulation [12] or no expression changes have been found [10,20,31]. At present, it is difficult to make generalizations about whether these results are host-specific or pathogen-specific, as different study organisms, life-history stages and experimental conditions were used (for review, see [22,24]). If the general *Dscam1* expression response is dependent upon the invading pathogen, then one might predict that different host species would respond to the same pathogen in a similar way, i.e. up, down or no regulation. Therefore, the second objective of our study was to test whether exposure to some of the same bacteria species affects *Dscam1* expression in our two model insect species in a similar way. For the majority of infections, we introduced bacteria through the cuticle and into the haemocoel via wounding. However, we also took advantage of the fact that one of the bacterial species, *Bacillus thuringiensis morrisoni* biovar *tenebrionis*, is orally infective for *T. castaneum* [32]. Irrespective of whether *Dscam1* expression was modulated by our bacterial treatments, we reasoned that knockdown of the gene might have a negative impact on *T. castaneum* survival after bacterial exposure. Our rationale was based on the fact that mosquitoes showed reduced survival after *Dscam1* knockdown and subsequent bacteria exposure [4]. Therefore, our third objective was to knockdown *Dscam1* by injection of double-stranded RNA (dsRNA), expose the larvae to bacteria and test survival relative to controls.

Vertebrate *DSCAM* [33] shows conserved protein structure with pancrustacean *Dscam1* [34,35] and, although *DSCAM* produces only two isoforms, the functions in neuronal development are also to some degree conserved across these taxa [35]. One consequence of *DSCAM*'s involvement in nervous system formation was that a reduction in mouse brain *DSCAM* mRNA levels resulted in decreased motor function: the walking posture was different compared with controls and movement was uncoordinated within three weeks following birth [36]. To the best of our knowledge, the possible role of *Dscam1* in motor function in insects has not been behaviourally examined. We therefore injected larval *T. castaneum* with dsRNA against *Dscam1*, allowed the individuals to go through metamorphosis where considerable neuronal rewiring takes place [37], and subsequently performed assays related to locomotor behaviour on the adult stage. In our assays, we took advantage of the fact that *T. castaneum* has an innate response to climb and adapted an assay from Michalczyk *et al.* [38] to test whether knockdown beetles showed reduced climbing abilities compared with controls. Additionally, we tested whether knockdown has an effect on movement by placing the beetles in an open arena; in the behavioural literature, this kind of open-field arena assay has been used to examine insect boldness [39]. Finally, we asked whether *Dscam1* might be important for reproductive success in *T. castaneum.* Little is known about how *Dscam1* affects adult fecundity, although homozygous *Dscam1* mutations are embryonically [3] and larvally [1] lethal. We therefore tested whether there are fecundity-related costs in *T. castaneum* at the adult stage when *Dscam1* knockdown is done in larvae; we predicted that knockdown would affect egg production and/or offspring viability.

## 2. Material and methods

### 2.1. Experimental organisms

As host insects we used wild-type *D. melanogaster* and *T. castaneum*, collected in Germany and Croatia respectively (please see electronic supplementary material, methods for more details, both here and throughout the methods). For infection experiments, *Bacillus thuringiensis morrisoni* biovar *tenebrionis* (hereafter *B. thuringiensis*; BGSCID 4AA1), *Escherichia coli* (DSM 498) and *Pseudomonas fluorescens* (DSM 50090) were used.

### 2.2. *Dscam1* nomenclature

In *D. melanogaster*, *Dscam* [1] and *Dscam-hv* [13] have been used synonymously, but the most recent nomenclature is *Dscam1* [8,40]. The orthologous gene in *T. castaneum* has also most recently been referred to as *Dscam1* [41]. Therefore, we have used *DmDscam1* to refer to *D. melanogaster Dscam1*, and *TcDscam1* to refer to *T. castaneum Dscam1*.

### 2.3. Experiments

#### 2.3.1. *Dscam1* expression in *Tribolium castaneum* and *Drosophila melanogaster*

We used three replicates of 10 pooled whole eggs, young and old larvae, pupae and adults, and three replicates of tissues from 10 animals, including brain, fat body, haemocytes from larvae and adults (*T. castaneum* only), and reproductive organs from females and males. RNA extraction was done with initial isolation in TRIzol reagent (Ambion, USA) and chloroform and further processing with the SV Total RNA Isolation System (Promega). Reverse transcription used random hexamer primers and SuperScript III™ (Invitrogen). Real-time quantitative PCR (RTqPCR) using gene-specific primers (*Dscam1* and two reference genes (*Rp49*, *RpL13a*); electronic supplementary material, table S1, and see Supplementary methods for an annotation of *TcDscam1*), was performed with Kapa SYBR^®^ Fast qPCR Mastermix (Peqlab Ltd) and a LightCycler480 (Roche).

#### 2.3.2. *Dscam1* expression after haemocoelic bacterial exposure in larval *Tribolium castaneum*

Fifteen-day-old *T. castaneum* larvae were pricked (haemocoelic exposure) with a needle previously dipped in a bacterial suspension (*B. thuringiensis*: 1 × 10^10^ ml^−1^, *E. coli*: 1 × 10^10^ ml^−1^ or *P. fluorescens*: 2 × 10^7^ ml^−1^), or with phosphate-buffered saline (treatment control), or they were not pricked (naive control). Bacterial concentrations here and for subsequent experiments were chosen based on preliminary survival experiments and transcriptomic data; for more details, please see the electronic supplementary material, Supplementary methods. We sampled fat body and haemocytes 6 and 18 h after treatment. The sampling times for this and the following infection experiments were chosen based on studies that found changes in *Dscam1* mRNA transcript frequency after bacterial infections [4,10–12]. We note that we cannot exclude the possibility that we might have had some fat body contamination in our haemolymph samples (e.g. as also suggested by Bartholomay *et al*. [42]). We produced six replicates per combination of treatment/tissue/time point, whereby each replicate consisted of tissue from 10 pooled animals, and an additional six animals were included to monitor survival (6 replicates × 5 treatments × 2 tissues × 2 time points × 16 animals = 1920 larvae). *Dscam1* expression analyses were performed as in the previous experiment. As a positive control that the immune system had been stimulated, we included three immunity genes: *Attacin2* (*Att2*), *Coleoptericin1* (*Col1*) and *Imd* (electronic supplementary material, table S1); for details regarding the choice of genes, please see electronic supplementary material, Supplementary methods*.* Relative fold expression differences between treatment groups and naive control groups were calculated according to Pfaffl [43] and statistical significance calculated using REST^©^ 2009 software [44].

#### 2.3.3. *Dscam1* expression after haemocoelic bacterial exposure in larval *Drosophila melanogaster*

Late second-instar *D. melanogaster* larvae were subjected to a similar experimental set-up as above, except that they were injected with *B. thuringiensis* (7.5 × 10^6^ ml^−1^) or *E. coli* (1 × 10^10^ ml^−1^), or *Drosophila* Ringer (treatment control), or left naive (6 × replicates × 4 treatments × 2 tissues × 2 time points × 16 animals = 1536 larvae). *Dscam1* expression analyses were performed as above; *Diptericin* (*Dpt*), *Drosomycin* (*Drs*) and *Imd* (electronic supplementary material, table S1) were the additional positive control genes.

#### 2.3.4. *Dscam1* expression after oral bacterial exposure in larval *Tribolium castaneum*

Seventeen-day-old *T. castaneum* larvae were exposed to a 1 × 10^9^ ml^−1^ *B. thuringiensis* spore-containing diet or to a diet containing no spores (control diet) [32]. The guts were removed 6 and 18 h post initial exposure giving a total of 6 replicates × 2 treatments × 1 tissue × 2 time points × 10 larvae = 240 larvae. Gene expression analyses were performed as above.

#### 2.3.5. Effect of *Dscam1* knockdown on larval *Tribolium castaneum* survival and development after haemocoelic bacterial exposure

To perform *Dscam1* knockdown in *T. castaneum* larvae, we used RNA interference (RNAi) following the protocol of Posnien *et al.* [45] using two non-alternatively spliced regions within the *Dscam1* gene, exons 12 (D-ex12^RNAi^) and 15 (D-ex15^RNAi^) (electronic supplementary material, table S1 and figure S1). As an RNAi treatment control (TC^RNAi^), we used a region of the *E. coli* gene *asparagine synthetase A*. Eleven day old larvae were injected with dsRNA (D-ex15^RNAi^: *n* = 256; TC^RNAi^: *n* = 256; dsRNA concentrations for this and the following experiments can be found in the electronic supplementary material, Supplementary methods). A third group was included as a naive control (N^RNAi^: *n* = 256). Four days after the dsRNA injections, whole bodies or haemocytes of 2 × 10 larvae per treatment were used to verify the knockdown using RTqPCR, as above. Twenty-seven larvae from each dsRNA group were pricked with a needle dipped into a bacterial suspension (*B. thuringiensis*: 1 × 10^10^ ml^−1^ or 3 × 10^10^ ml^−1^; *E. coli*: 1 × 10^11^ ml^−1^), received a wound only, or were left as naive controls. Survival and development were monitored for 7 days. Larval survival over the 7 days following haemocoelic bacterial exposure was analysed using a mixed-effects Cox proportional hazards model in R [46,47] (RStudio v. 0.99.441 for Macintosh). Development time to pupa and adult was analysed using JMP v. 9.0.0 for Macintosh OS X.

#### 2.3.6. Effect of *Dscam1* knockdown on larval *Tribolium castaneum* survival after oral bacterial exposure

Eleven-day-old larvae were injected with dsRNA (D-ex15^RNAi^: *n* = 192; TC^RNAi^: *n* = 192; N^RNAi^: *n* = 192). Four days after the dsRNA injections, 3 × 5 larvae from each treatment group were sampled to verify the knockdown for whole body and gut, respectively. Forty-eight larvae from each dsRNA group were randomly assigned to either 5 × 10^9^ ml^−1^ *B. thuringiensis* spore exposure or to a control diet. The oral bacterial exposure was performed as described above and survival was monitored for 4 days. Larval survival after spore exposure was analysed as described above.

#### 2.3.7. Life-history effects of *Dscam1* knockdown in *Tribolium castaneum*

*Behavioural tests.* For this section (§2.3.7), we used the same animals for behavioural and then fecundity experiments. We injected 11 day old larvae with dsRNA (D-ex12^RNAi^: *n* = 144; D-ex15^RNAi^: *n* = 144; TC^RNAi^: *n* = 192; N^RNAi^ (for behavioural assays only): *n* = 96). The knockdown was verified 25 days post-knockdown, from 3 × 10 animals of every sex and treatment combination. Ten males and females from each of the four treatment groups were tested with the behavioural assays. We adapted an assay from Michalczyk *et al.* [38] to test the speed at which beetles vertically climb. The beetles were offered a strip of paper, and we measured the time that it took for a beetle to completely pass a 30 mm mark. To investigate beetle behaviour in an open arena [39], the beetles were placed in the centre of an open arena (diameter: 195 mm, wall height: 51 mm) surrounded by a dark circular wall and given 2 min to reach the wall [39]. Representative beetles were filmed with a Canon EOS 5D mark II camera. The time taken for N^RNAi^ and TC^RNAi^ beetles to reach the wall was analysed using an ANOVA in JMP^®^ v. 9.0.0 for Macintosh.

*Fecundity test.* After the behavioural tests, we used the same dsRNA-injected animals (D-ex12^RNAi^: *n* = 144; D-ex15^RNAi^: *n* = 144; TC^RNAi^: *n* = 192) to test the effect of *Dscam1* knockdown on fecundity. Individuals were paired in seven combinations, *n* = 20 pairs per combination; female first: TC^RNAi^ × TC^RNAi^; TC^RNAi^ × D-ex12^RNAi^; TC^RNAi^ × D-ex15^RNAi^; D-ex12^RNAi^ × TC^RNAi^; D-ex15^RNAi^ × TC^RNAi^; D-ex12^RNAi^ × D-ex12^RNAi^; D-ex15^RNAi^ × D-ex15^RNAi^. The pairs were sieved every 3 days for a total of 12 days. Fecundity was measured as the total number of eggs laid, and the proportion of eggs hatched. Female survival was analysed as described previously. An ANOVA was used to test whether the TC females differed significantly in the number of eggs that they laid depending upon the male they had been paired with.

#### 2.3.8. Mating behaviour and physiology

In a separate experiment to §2.3.7, we injected 11 day old larvae with dsRNA (D-ex12^RNAi^: *n* = 72; D-ex15^RNAi^: *n* = 72; TC^RNAi^: *n* = 72; N^RNAi^: *n* = 72). We set up female–male pairings in 10 combinations, *n* = 5 pairs per combination; female first: TC^RNAi^ × TC^RNAi^; TC^RNAi^ × D-ex12^RNAi^; TC^RNAi^ × D-ex15^RNAi^; D-ex12^RNAi^ × TC^RNAi^; D-ex15^RNAi^ × TC^RNAi^; N^RNAi^ × N^RNAi^; N^RNAi^ × D-ex12^RNAi^; N^RNAi^ × D-ex15^RNAi^; D-ex12^RNAi^ × N^RNAi^; D-ex15^RNAi^ × N^RNAi^. Prior to pairing, using a dissection microscope, males and females were checked for externally everted genitalia [41]. Each pair was given a maximum of 1 h during which we observed them continuously to determine mating latency and copulation duration. An attempted copulation was defined as an interaction where genital contact appeared to be maintained for at least 35 s [48]. Females were separated from the males, and after 3 days fecundity was measured as described above. A subset of 20 females were photographed and dissected on the same day that the eggs were counted and ovaries were photographed under a dissecting microscope.

On the same day as the pairs were set up, five males and five females from each of the four treatment groups were placed individually into a thin glass tube with a strip of paper lining the bottom. The last 30 mm of the tube were covered with a piece of black cotton. We filmed the beetles laterally, and we left them inside the tube for a maximum of 1 min and noted whether or not they reached the darkened area of the tube.

## 3. Results and discussion

### 3.1. *Dscam1* expression in *Tribolium castaneum* and *Drosophila melanogaster*

*DmDscam1* is expressed by neurons, fat body cells and haemocytes [2], but to date *TcDscam1* expression has not been examined. We found that *DmDscam1* and *TcDscam1* were expressed in all life-history stages (electronic supplementary material, figure S2*a*) and tissues (electronic supplementary material, figure S2*b*) examined. In general, the constitutive expression of *Dscam1* was low relative to the reference genes (*Rp49* and *RpL13a*). Indeed, the only tissue in which *Dscam1* expression was higher than the reference genes was in the brain of *T. castaneum* larvae and adults (electronic supplementary material, figure S2*b*). Comparatively high expression of *Dscam1* in the brain was also observed in the crustaceans *Pacifastacus leniusculus* [10] and *Eriocheir sinensis* [12], and is not surprising given its vital nervous system function. We observed that larval and adult *T. castaneum* showed expression of *TcDscam1* in immune tissues. *Dscam1* was expressed in the reproductive organs of both species (electronic supplementary material, figure S2*b*), which has previously only been reported for two crustacean species: the testes of *P. leniusculus* ([10]; ovaries were not examined) and the ovaries and testes of *E. sinensis* [12,49]. Although during dissections of the reproductive organs we attempted to remove any non-reproductive tissue, we cannot exclude the possibility that the samples may have contained haemocytes, fat body or neurons that e.g. innervate the lateral oviduct and peritoneal sheath [50], and thus these may have contributed to the overall *Dscam1* expression that we detected in the reproductive tissues.

### 3.2. *Dscam1* expression after haemocoelic bacterial exposure in larval *Tribolium castaneum* and *Drosophila melanogaster*

The role that *Dscam1* plays in the immune system of pancrustaceans has been the subject of a number of studies (reviewed in [22–24]), nevertheless as of yet it is difficult to disentangle whether changes in *Dscam1* expression after pathogen exposure are host-specific or pathogen-specific. Here, we tested whether the fat body or haemocytes showed increased *Dscam1* transcription in two model insect hosts after haemocoelic exposure to bacteria. As a positive control that the immune system had been activated, we tested the expression of three immune genes for each host species: *Imd* and two species-specific antimicrobial peptides (AMPs), *Att2* and *Col1* for *T. castaneum* [51–53] and *Dpt* and *Drs* for *D. melanogaster* [53–56].

**Figure 1.**
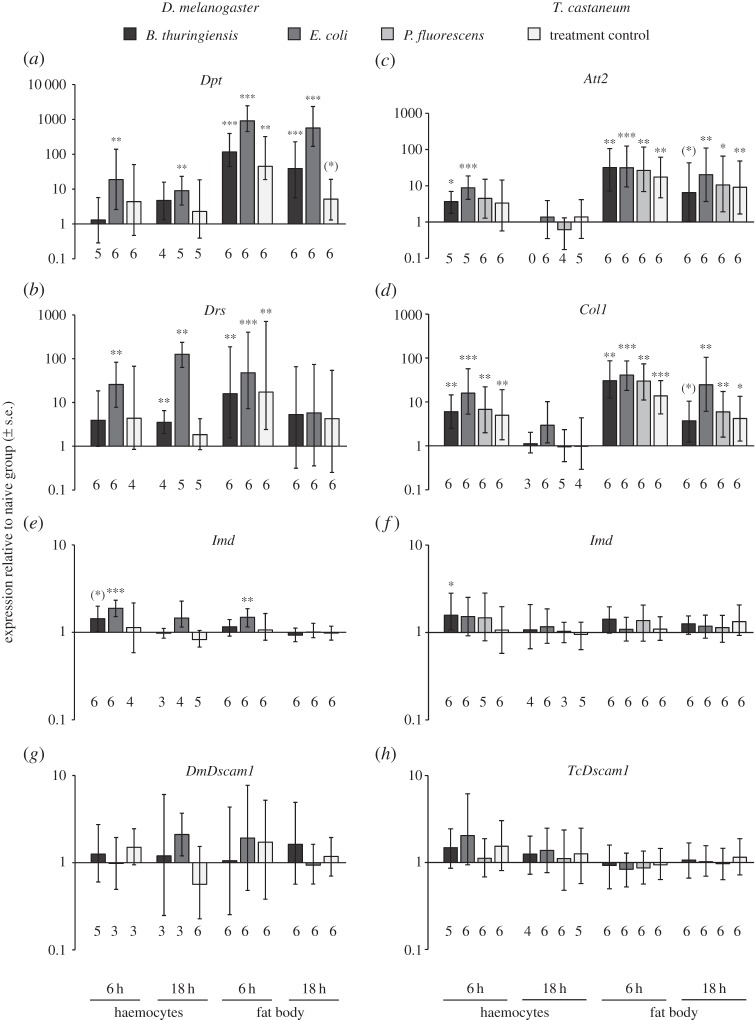
*D.~melanogaster* and *T. castaneum* larval gene expression after haemocoelic bacterial exposure. Expression is shown relative to the naive group at 6 and 18 h after haemocoelic bacterial exposure to *B. thuringiensis*, *P. fluorescens* or *E. coli*, or after a treatment control. The expression of (*a–d*) two antimicrobial peptide genes, (*e,f*) *Imd* and (*g,h*) *DmDscam1* and *TcDscam1* from the fat body and haemocytes are shown relative to the naive control groups. The expression of the reference genes *Rp49* and *RpL13a* was used to normalize the expression of the target genes. Means and standard errors are as calculated according to the REST^©^ software [44], i.e. the results of the 2000 random reallocations. Means that are significantly different from the control group after Benjamini Hochberg (false discovery rate, FDR) corrections are indicated: *p ≤ 0.05, **p ≤ 0.01 and ***p ≤ 0.001. Means that were significant before FDR are indicated by parentheses (*). The numbers of replicates are given under each figure; each replicate contains the fat body or haemocytes of 10 animals.

For both host species, we found a significant increase in AMP expression in bacteria-exposed groups as well as the treatment control relative to the naive control groups, and the responses were tissue, time point and bacteria-specific (figure 1*a–d*), verifying that all bacteria and wounding alone led to immune activation. In contrast to the strong upregulation of AMPs, *Imd* showed less than a twofold upregulation, which was statistically significant in some comparisons (figure 1*e,f*)*.* In a subsequent analysis, we also compared bacteria-exposed insects to the treatment control group (see electronic supplementary material, figure S3). Given that, to the best our knowledge, this is the first study in *T. castaneum* to test expression of these genes in haemocytes and fat body after infection with bacteria, it is unclear whether these genes do not significantly increase in expression under these conditions, or whether the time point after exposure or the bacteria dose were responsible.

Similar to the previous experiment (electronic supplementary material, figure S2), overall the *Dscam1* expression level in the haemocytes and fat body was low compared with the reference genes and AMPs (see raw data file for Cp values). Furthermore, in some cases, the standard errors were quite large, which may partly be owing to a reduced number of replicates in some groups (see figure 1*g* and electronic supplementary material, Supplementary methods). We note that there are other experiments where *Dscam1* expression showed increased variance at some time points after infection compared with others [30] and also where there was low variance [10]. Unlike the AMPs and *Imd*, neither *DmDscam1* nor *TcDscam1* showed significant changes in expression after wounding or after haemocoelic bacterial exposure (figure 1*g,h* and electronic supplementary material, figure S3*g,h*). Similar observations were made in a transcriptome analysis, where no significant changes in *TcDscam1* transcript frequencies were detected in whole larval bodies after haemocoelic exposure to live *B. thuringiensis* [57], and RTqPCR data from *D. melanogaster*, where no change in *DmDscam1* expression was found in adult bodies after haemocoelic exposure to *E. coli* [20]. Although some studies using similar infection methodology have found significant *Dscam1* upregulation in a short-term time frame that is similar to our experiment [10,12], other studies have reported transcriptional changes at time points of up to 3 days post-pathogen exposure [15]. Therefore, we cannot exclude the possibility that transcriptional changes might have occurred at a later time point, or indeed using a different bacterial dose.

### 3.3. *Dscam1* expression after oral bacterial exposure in larval *Tribolium castaneum*

Recent studies in *D. melanogaster* and *T. castaneum* have suggested that the route of infection, i.e. oral bacterial exposure versus haemocoelic exposure, plays a critical role in host defence [57,58] and elicits the expression of different genes and pathways against the same pathogen [57]. Therefore, we tested whether oral exposure to *B. thuringiensis* affects *TcDscam1* expression. As a positive control, we tested whether *B. thuringiensis* oral exposure resulted in the upregulation of immunity genes in the gut. Because previous studies have observed modulation of AMP expression in the gut after feeding with bacteria [59] or a *B. thuringiensis* toxin [60], we predicted an upregulation in *Att2, Col1* or *Imd* expression after exposure to *B. thuringiensis*. However, we found no significant changes in immune genes or in *TcDscam1* (electronic supplementary material, figure S4). Contrary to our findings, *Apis mellifera ligustica* that were orally exposed to a microsporidium or a trypanosome showed a significant upregulation of *Dscam1* in the abdomen 6 h after exposure [11]. Interestingly, Schwarz & Evans [11] also found upregulation at later time points, i.e. 24 and 72 h in the abdomen, and 72 and 120 h in the gut. There are a number of differences between that study [11] and the present study in terms of the host and the pathogens used, so it is unfortunately not possible to make any generalizations. Schwarz & Evans [11] pointed out that differences in *Dscam1* expression in their study might vary as a result of contamination by haemocytes, fat body and nervous tissue in the sampled gut and abdomen. However, it might be interesting to note that in a whole larval body analysis, Behrens *et al.* [57] found no significant change in expression of *TcDscam1* at 6 or 18 h after oral exposure to *B. thuringiensis*.

Despite the lack of expression change in total *Dscam1* after bacterial exposure, the expression of specific *DmDscam1* and *TcDscam1* alternatively spliced variants might have been affected by the bacteria exposure, as was shown for *Anopheles gambiae* [4]. We are unable to examine this with our dataset, however neither Armitage *et al*. [20] nor Smith [21] found significant changes in the composition of *DmDscam1* isoforms after exposing adult *D. melanogaster* to *E. coli*, one of the bacteria used in our study.

### 3.4. Effect of *Dscam1* knockdown on larval *Tribolium castaneum* survival and development after haemocoelic or oral bacterial exposure

Bacterial exposure did not result in expression changes in *Dscam1*. A previous study on *A. gambiae* also found that infection did not affect overall transcript levels, but *AgDscam* RNAi and subsequent infection with *Staphylococcus aureus* and *E. coli* resulted in reduced survival [4]. To test whether *Dscam1* is essential for survival after infection in *T. castaneum,* we used RNAi to knockdown *TcDscam1* exon 15 (D-ex15^RNAi^; electronic supplementary material, figure S1, for knockdown efficiencies see figure S5). Four days later, which is a similar time frame to that used by Dong *et al*. [4], we exposed *T. castaneum* larvae to haemocoelic *B. thuringiensis* infection. Contrary to our expectations, *Dscam1* knockdown did not affect survival after haemocoelic exposure: there was no significant interaction between the knockdown and the infection treatment (*χ*^2^ = 1.23, d.f. = 8, *p* = 0.99; electronic supplementary material, figure S6*a–e*). There was also no effect of the knockdown treatment itself (*χ*^2^ = 0.70, d.f. = 2, *p* = 0.70). As expected, there was a significant overall effect of bacterial exposure treatment (*χ*^2^ = 243.6, d.f. = 4, *p* < 0.0001), where in pairwise comparisons with the naive group the *B. thuringiensis*-infected larvae had poorer survival (naive versus *B. thuringiensis* 1 × 10^10^ ml^−1^: *z* = 5.542, *p* < 0.0001; naive versus *B. thuringiensis* 3 × 10^10^ ml^−1^: *z* = 7.089, *p* < 0.0001).

Overall, the developmental speed of the surviving larvae was not greatly affected by the knockdown (electronic supplementary material, Supplementary methods). In agreement with our results from the haemocoelic exposure, we found no effect of *TcDscam1* knockdown on survival after oral exposure to *B. thuringiensis* spores (electronic supplementary material, figure S6*f,g*), i.e. there was no significant interaction between the knockdown and the infection treatment (*χ*^2^ = 0.04, d.f. = 2, *p* = 0.98). These findings are contrary to those of Dong *et al*. [4,27], where knockdown of *AgDscam* resulted in an increased permissiveness towards the orally infecting malaria parasite. One explanation is that *Plasmodium* and *B. thuringiensis* have different infection processes thus potentially explaining the discrepancy. We found no effect of the knockdown treatment itself on survival (*χ*^2^ = 0.30, d.f. = 2, *p* = 0.86). However, as expected, spore-exposed larvae had a higher risk of death than control larvae (*χ*^2^ = 16.72, d.f. = 1, *p* < 0.0001).

Why did we not find the predicted effect of the knockdown on survival after bacteria exposure? The simplest explanation is that *TcDscam1* is not essential for survival after infection with the bacteria that we used. A number of non-mutually exclusive alternative possibilities exist, e.g. we do not know the half-life of the Dscam1 protein, perhaps it is still present in sufficient quantities to be functional 4 days after the knockdown. Alternatively, the knockdown might not have been strong enough to detect immune phenotypes (however, see results for behaviour below). Second, most studies testing *Dscam1* in pancrustacean immunity have used the adult stage for their experiments (reviewed in [22]), whereas we used larvae; one hypothesis is that *Dscam1* could serve different functions in different life-history stages. Third, the classical immune pathways in *T. castaneum* [51] may compensate for any loss of functional *TcDscam1*. Fourth, perhaps other bacteria concentrations, or indeed other pathogen or parasites species could result in lower survival after knockdown. At the moment these remain open questions.

### 3.5. Effect of *Dscam1* knockdown on *Tribolium castaneum* behaviour

Although we did not find an effect of *TcDscam1* knockdown on survival after bacteria exposure, the same concentration of dsRNA produced strong effects on adult behaviour. Both dsRNA injection treatments resulted in poorer adult motility compared with controls. A simple observation we made was that on the morning of the behavioural assays, only one control beetle was dorsal side down, yet a minimum of 40% of *TcDscam1* knockdown beetles were in this orientation (electronic supplementary material, figure S7). In the climbing assay, despite all beetles being able to grip onto the paper, none of the *TcDscam1* knockdown beetles successfully climbed over 30 mm, whereas a minimum of 90% of the control groups were successful (electronic supplementary material, figure S8*a*). In the open arena test, 100% of the controls reached the wall, whereas only three of the *TcDscam1* knockdown beetles did so (electronic supplementary material, figure S8*b,c* and movie S1–S4). Our results suggest that RNAi targeting either constitutive D-ex12 or D-ex15 results in a similar knockdown efficiency (electronic supplementary material, figure S5*b*) as well as a similar adult phenotype. In the pupal stage of holometabolous insects, neurons undergo a process of neuronal degeneration and replacement and existing larval neurons are remodelled [37]. Therefore, we hypothesize that the reduced locomotion ability that we observed at the adult stage might be due to neuronal wiring being negatively affected by reduced *Dscam1* expression during pupation, although this remains to be tested.

### 3.6. Effect of *Dscam1* knockdown on *Tribolium castaneum* fecundity

**Figure 2.**
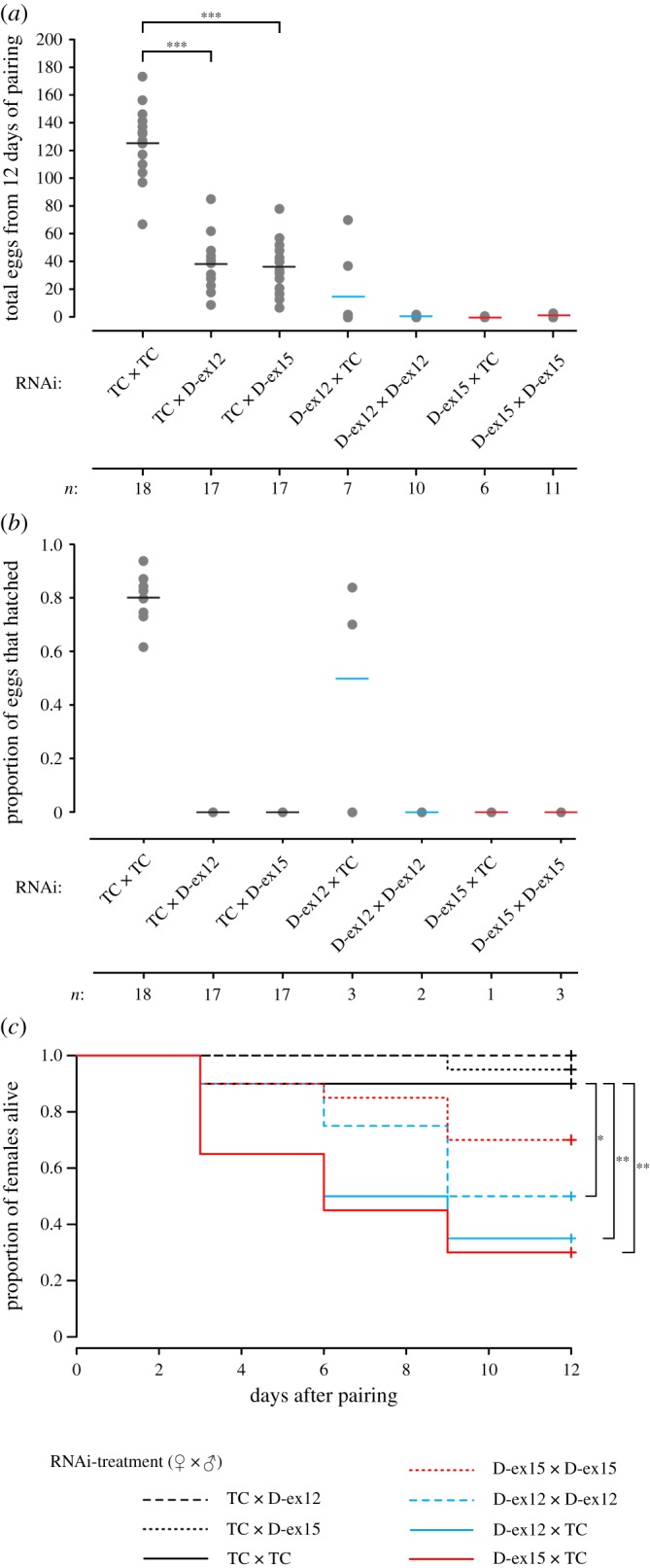
Fecundity and survival of *T. castaneum* adults after *Dscam1* knockdown. Fecundity was measured as (*a*) the total number of eggs laid over 12 days of pairing and (*b*) the proportion of hatched eggs per pair. The pairing treatment is indicated beneath the figure with female RNAi treatment first; grey dots show the value for each pair and the means are shown as a line. Only values for pairs where the female and male survived the full 12 days are shown; ‘*n*’ below the figures indicates the number of pairs. (*c*) Survival of female beetles after pairing with males. Pairing treatments are indicated below the figure. Each curve is the cumulative survival of 20 paired females and significant differences compared with TC^RNAi^ × TC^RNAi^ pairings are shown by *p ≤ 0.05 and **p ≤ 0.01. TC indicates the RNAi treatment control, and D-ex12 and D-ex15 indicate the two *Dscam1* RNAi knockdown groups, where ex12 and ex15 refer to *Dscam1* non-alternatively spliced exons.

Because our study (electronic supplementary material, figure S2*b*) and others [10,12,49] have found *Dscam1* expression in the reproductive organs, we examined whether knockdown via injection of dsRNA affects offspring production. In general, there was a strong effect of the knockdown on female and male fecundity. Only nine (26%) *TcDscam1* dsRNA-injected females that survived to the end of the experiment laid eggs, and of these females, the majority (seven out of nine) laid three or fewer eggs (figure 2*a*). In an RNAi screen, Dönitz *et al.* [41] also showed that egg production was lower than expected in females after *TcDscam1* knockdown, where dsRNA of exons 11–13 had been injected. Furthermore, the number of eggs that females from the RNAi treatment control (TC^RNAi^) group laid was dependent upon the male with whom she had been paired (*F*_2,49_ = 109.46, *p* < 0.0001; figure 2*a*); females laid more eggs when paired with a TC^RNAi^ male compared with either of the D-ex12^RNAi^ or D-ex15^RNAi^ males (*p* < 0.0001 for both comparisons), but there was no significant difference between D-ex12^RNAi^ and D-ex15^RNAi^ (*p* = 0.879). With the exception of two D-ex12 females paired to TC^RNAi^ males, only TC^RNAi^ × TC^RNAi^ pairings produced larvae (figure 2*b*). These results indicate that both males and females pay a strong fecundity cost to *TcDscam1* knockdown.

The risk of a female dying was significantly affected by a combination of her own knockdown treatment and that of her partner (*χ*^2^ = 48.84, d.f. = 6, *p* < 0.0001; figure 2*c*), which therefore affected the sample sizes in the above fecundity analyses (figure 2*a*). It is unclear why the knockdown generally resulted in higher female mortality than in the control, one possibility is that the knockdown has a negative effect on feeding rate, and hence survival. Pertinent to this possibility, Dönitz *et al.* [41] found that *Dscam1* knockdown beetles were in a bad nutritional state 22 days post-dsRNA injection, which we suggest could also indirectly affect fecundity and locomotion.

### 3.7. Effect of *Dscam1* knockdown on *Tribolium castaneum* mating behaviour and physiology

Our final objective was to attempt to understand more about the point in the reproductive process at which *TcDscam1* knockdown affects fecundity. For example, virgin *T. castaneum* are able to lay eggs, so we do not know whether *TcDscam1* knockdown males were physically unable to copulate with females because of motility impairment, they were unable to successfully transfer sperm to females, or the sperm was unable to fertilize the eggs. Therefore, we observed attempted copulations and allowed any laid eggs to hatch, revealing which of any attempted copulations were successful.

Prior to pairing we observed that 70% of female and 21% of male *TcDscam1* knockdown individuals exhibited everted genitalia (electronic supplementary material, table S2 and figure S9). This phenomenon has previously been described for ovipositors of *TcDscam1* knockdown females [41]. At present, it is unclear why the ovipositors should be everted. All N^RNAi^ and TC^RNAi^ males attempted to mate, regardless of the female treatment and ovipositor eversion (electronic supplementary material, table S2). However, none of the *TcDscam1* knockdown males attempted to mate (electronic supplementary material, table S2) in the hour of observations. One hour should have been ample time for mating to take place given that the mating latency of the control treatment pairings was short (mean latency in seconds ± 1 s.e.: N^RNAi^ × N^RNAi^ = 199 ± 95; TC^RNAi^ × TC^RNAi^ = 60 ± 30). The absence of mating most likely explains the lack of larvae resulting from pairings in our previous experiment where males were from the *TcDscam1* knockdown (figure 2*b*). Naive and TC beetles passed through the glass tube within 1 min, but only one out of 20 knockdown beetles reached the darkened area of the tube. The movements of the knockdown beetles were generally slower and they moved in what appeared to be an uncoordinated manner (electronic supplementary material, movies S5–S8). Further analyses would be necessary to describe this phenomenon in more depth.

Similar to the previous experiment, *TcDscam1* knockdown females produced few eggs (electronic supplementary material, figure S10). Except for one D-ex12^RNAi^ × N^RNAi^ pairing, none of the *TcDscam1* knockdown females produced larvae (electronic supplementary material, figure S10), which is consistent with the findings of our previous experiment. In contrast, all observed copulations between control beetles, except for one, produced larvae, indicating that these matings were largely successful (electronic supplementary material, figure S10).

The *TcDscam1* knockdown females had reduced ovariole differentiation, and when they contained eggs, the eggs were occasionally misshapen (electronic supplementary material, figure S9). These results give potential proximate causes for the lack of viable offspring from *TcDscam1* depleted females, although our sample size was low (*n* = 8 dissected knockdown females). The abnormal ovary morphology might be explained by feeding status (see discussion regarding Dönitz *et al.* [41] above) or incorrect development during pupal metamorphosis caused by depletion of *Dscam1*.

## 4. Conclusion

Recent studies describe *Dscam1* as a molecule with dual functions. It acts as a neuronal guidance factor in the nervous system and as an immune factor that contributes to defence against certain pathogens. Here, we have taken a multi-pronged approach to understand how *Dscam1* affects fitness. The data do not support an essential pathogen defence role for *Dscam1* under our experimental conditions. However, this study shows that the knockdown of *Dscam1* has strong consequences for locomotor behaviour and fecundity, underscoring *Dscam1*'s essential function in developmental processes and fitness-related traits, in addition to other work showing it acting as an immune factor.

## Supplementary Material

Figure S1. Schematic illustration of genomic and mRNA Dscam1 sequences from D. melanogaster and T. castaneum.

## Supplementary Material

Figure S2. Dscam1 expression in different life history stages and tissues of T. castaneum and D. melanogaster.

## Supplementary Material

Figure S3. D. melanogaster and T. castaneum larval immune gene expression relative to the treatment control group 6 and 18 hours after haemocoelic bacterial exposure to B. thuringiensis, P. fluorescens or E. coli.

## Supplementary Material

Figure S4. T. castaneum larval gut gene expression after oral exposure to B. thuringiensis spores.

## Supplementary Material

Figure S5. Expression of T. castaneum Dscam1 relative to the treatment control (TCRNAi) after injection of Dscam1 dsRNA.

## Supplementary Material

Figure S6. Survival of T. castaneum larvae after Dscam1 knockdown and haemocoelic bacterial or oral bacterial exposure.

## Supplementary Material

Figure S7. The proportion of adult T. castaneum found on their backs after larval knockdown of Dscam1.

## Supplementary Material

Figure S8. Climbing and open arena assays of control and TcDscam1 dsRNA-injected adult T. castaneum.

## Supplementary Material

Figure S9. Morphology of control and TcDscam1 dsRNA-injected female beetles and their reproductive organs.

## Supplementary Material

Figure S10. Fecundity of control and TcDscam1 ds-RNA-injected females from pairings with differently treated males.

## Supplementary Material

Table S1. Primer sequences.

## Supplementary Material

Table S2. Summary of results of pairing behaviour after Dscam1 knockdown in T. castaneum.

## Supplementary Material

Supplementary Methods.

## Data accessibility

The raw data supporting the results of this manuscript have been deposited in the Dryad digital repository and are available at http://dx.doi.org/10.5061/dryad.0k0mt.
