# Supplementary material for: Down syndrome cell adhesion molecule 1: testing for a role in insect immunity, behaviour and reproduction
Source: R Soc Open Sci. 2016 Apr 20;3(4):160138. doi: 10.1098/rsos.160138 (PMC4852650; doi:10.1098/rsos.160138)
Supplement: Figure S1. Schematic illustration of genomic and mRNA Dscam1 sequences from D. melanogaster and T. castaneum. [file rsos160138supp1.pdf]

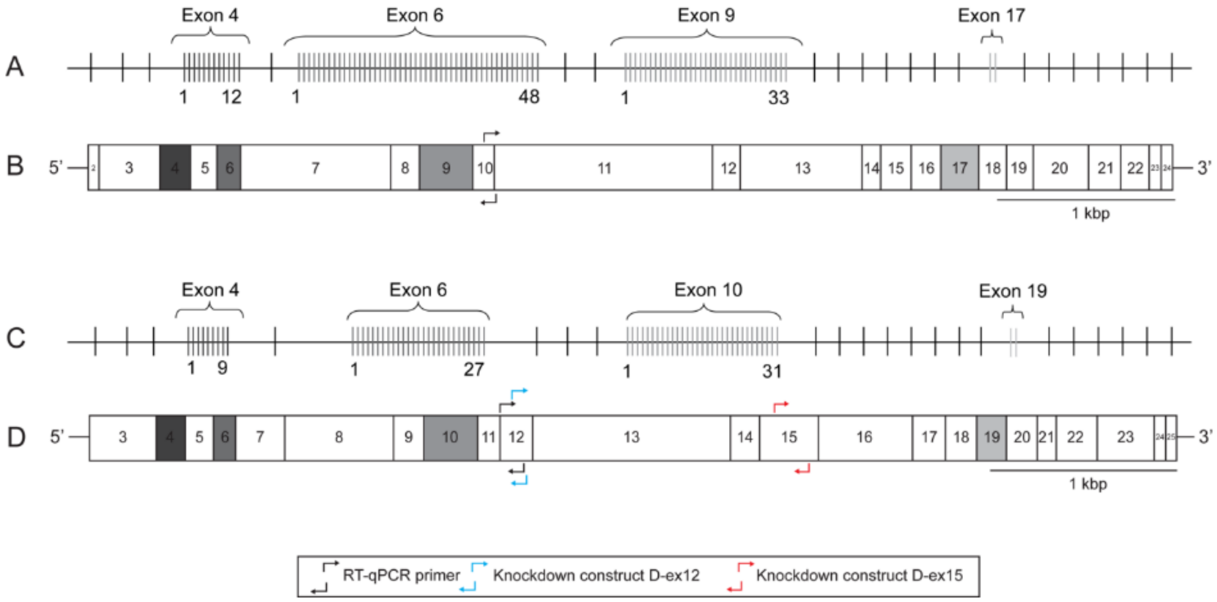

Figure S1. Schematic illustration of genomic and mRNA *Dscam1* sequences from *D. melanogaster* and *T.* castaneum. (**A**) Genomic illustration of *D. melanogaster Dscam1* (gene accession no.: FBgn0033159). (**B**) *D. melanogaster Dscam1* mRNA with corresponding exon numbers. (**C**) Genomic illustration of *T. castaneum Dscam1* (gene accession no.: TC012539). (**D**) *T. castaneum Dscam1* mRNA with corresponding exon numbers. Alternatively spliced exons are indicated in grey (with number of different exons given). Mutually exclusive alternative splicing occurs for exon 4, 6, 9 and 17 in (**A**) and for 4, 6, 10 and 19 in (**C**) which creates a vast number (ca. 38,000 [**A**] and ca. 15,000 [**B**]) of potential splice variants. Black arrows in (**B**) and (**D**) indicate the position of RTqPCR primers for expression analyses. Turquoise (D-ex12) and red (D-ex15) arrows in (**D**) indicate the position of knock-down constructs for RNAi mediated depletion of *Dscam1* in *T. castaneum*, and this colour scheme is used throughout the figures. Scale in (**B**) and (**D**) indicates 1 kbp.
