# Supplementary material for: Down syndrome cell adhesion molecule 1: testing for a role in insect immunity, behaviour and reproduction
Source: R Soc Open Sci. 2016 Apr 20;3(4):160138. doi: 10.1098/rsos.160138 (PMC4852650; doi:10.1098/rsos.160138)
Supplement: Figure S2. Dscam1 expression in different life history stages and tissues of T. castaneum and D. melanogaster. [file rsos160138supp2.pdf]

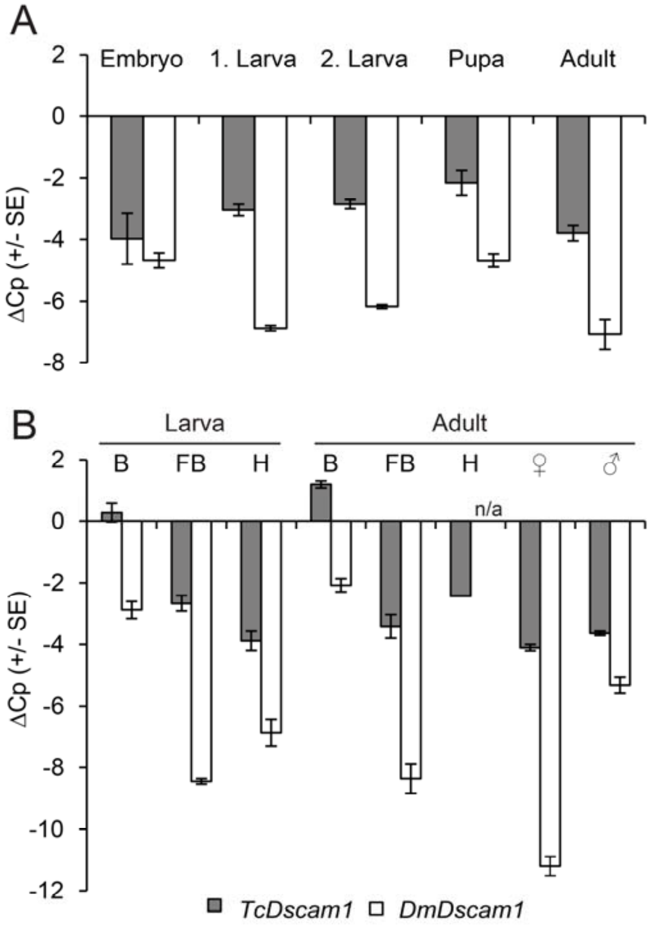

Figure S2. *Dscam1* expression in different life history stages and tissues of *T. castaneum* and *D. melanogaster*. (A) *Dscam1* expression in whole body samples. 1. Larva refers to 1st instar larvae in *D. melanogaster* and to 3rd -4th instar larvae in *T. castaneum*; 2. Larva refers to 3rd instar larvae in *D. melanogaster* and to 6th - 7th instar larvae in *T. castaneum*. **(B)** *Dscam1* expression in adult and larval tissues. Brain [B], haemocytes [H], fat body [FB] were obtained from 3rd instar larvae in *D. melanogaster* and 6th - 7th instar larvae in *T. castaneum*. Reproductive organs from females [♀], and males [♂], [B], [H] and [FB] were sampled from 1-2 week old adults. (n/a) we could not obtain haemolymph in sufficient quantities. Error bars indicate one standard error (SE). *Dscam1* expression was quantified relative to the geometric mean of the two reference genes, *Rpl13a* and *Rp49*, and apart from the brain samples from *T. castaneum*, *Dscam1* was expressed at a lower level than the reference genes, hence the negative values. n = 3 biological replicates for each bar, each replicate contained 10 animals.
