# Supplementary material for: Down syndrome cell adhesion molecule 1: testing for a role in insect immunity, behaviour and reproduction
Source: R Soc Open Sci. 2016 Apr 20;3(4):160138. doi: 10.1098/rsos.160138 (PMC4852650; doi:10.1098/rsos.160138)
Supplement: Figure S3. D. melanogaster and T. castaneum larval immune gene expression relative to the treatment control group 6 and 18 hours after haemocoelic bacterial exposure to B. thuringiensis, P. fluorescens or E. coli. [file rsos160138supp3.pdf]

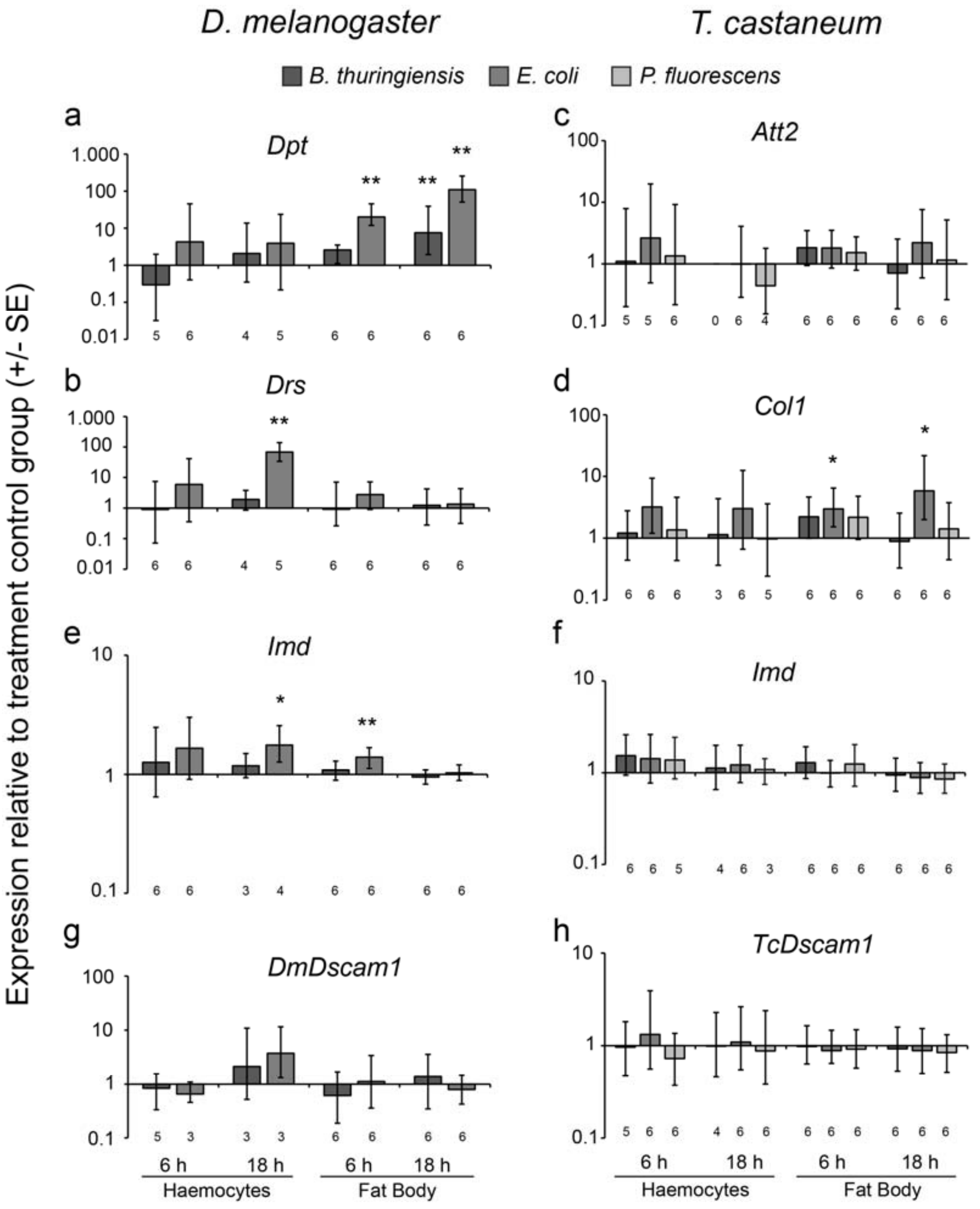

Figure S3. ***D. melanogaster*** **and** ***T. castaneum*** **larval immune gene expression relative to the treatment control group 6 and 18 hours after haemocoelic bacterial exposure to** ***B. thuringiensis*****,** ***P. fluorescens*** **or** ***E. coli*****.** The expression of two antimicrobial peptide genes (**a**: *Diptericin* [*Dpt*] and **b**: *Drosomycin* [*Drs*] for *D. melanogaster* and **c**: *Attacin2* [*Att2*] and **d**: *Coleoptericin1* [*Col1*] for *T. castaneum*) *Imd* (**e** & **f**) and *Dscam1* (**g** & **h**) in *D. melanogaster* fat body and haemocytes and *T. castaneum* fat body and haemocytes are shown relative to the treatment control groups. Means that were significantly different from the control group after Benjamini Hochberg (FDR) corrections are indicated with: * for $p \leq 0.05$, ** for $p \leq 0.01$ and *** for $p \leq 0.001$. Means that were significant before FDR are indicated with $^{(*)}$. Each mean is calculated from given number of replicates, each replicate containing the fat body or haemocytes of 10 animals. For *D. melanogaster* this analysis indicated that the response against *B. thuringiensis* and *E. coli* can be higher than against the injection of buffer alone. For *T. castaneum, E. coli* induced increased AMP expression, but there were less distinct differences for the other bacteria.
