# Supplementary material for: Down syndrome cell adhesion molecule 1: testing for a role in insect immunity, behaviour and reproduction
Source: R Soc Open Sci. 2016 Apr 20;3(4):160138. doi: 10.1098/rsos.160138 (PMC4852650; doi:10.1098/rsos.160138)
Supplement: Figure S4. T. castaneum larval gut gene expression after oral exposure to B. thuringiensis spores. [file rsos160138supp4.pdf]

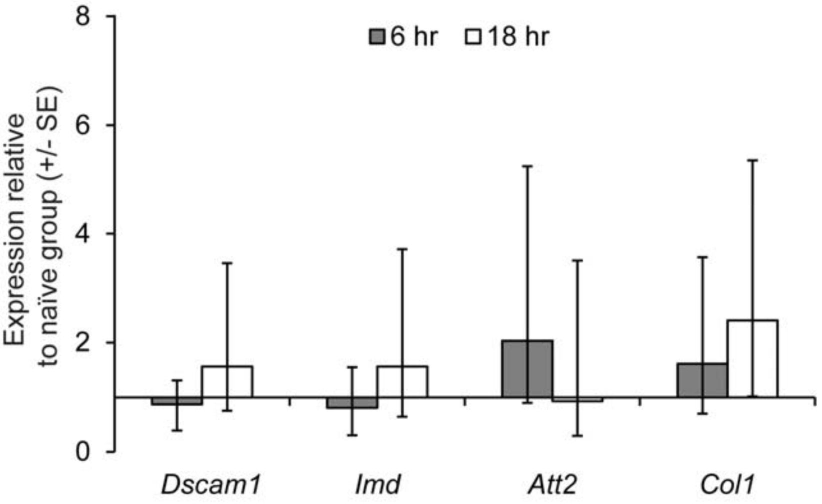


**Figure S4. *T. castaneum* larval gut gene expression after oral exposure to *B. thuringiensis* spores.** The expression of *Dscam1*, *Imd* and two antimicrobial peptide genes in the gut is shown relative to the naïve control groups at 6 and 18 hours after bacteria exposure. Bacteria exposed larvae did not differ significantly from the control larvae. Each mean is from six biological replicates, each replicate containing the guts of 10 animals.
