# Supplementary material for: Down syndrome cell adhesion molecule 1: testing for a role in insect immunity, behaviour and reproduction
Source: R Soc Open Sci. 2016 Apr 20;3(4):160138. doi: 10.1098/rsos.160138 (PMC4852650; doi:10.1098/rsos.160138)
Supplement: Figure S5. Expression of T. castaneum Dscam1 relative to the treatment control (TCRNAi) after injection of Dscam1 dsRNA. [file rsos160138supp5.pdf]

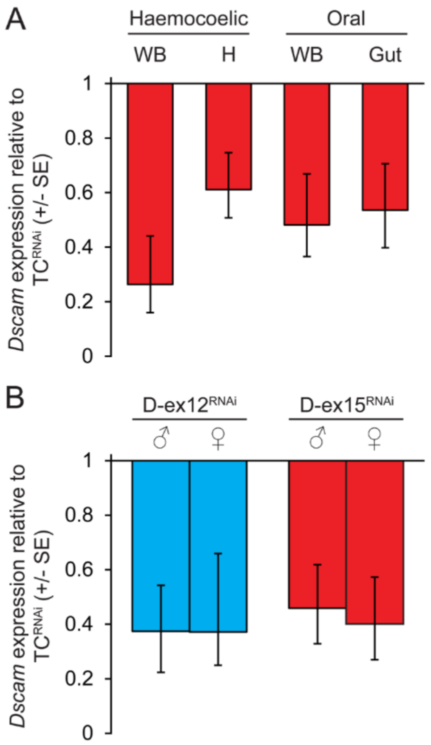

Figure S5. Expression of *T. castaneum Dscam1* relative to the treatment control (TC^{RNAi}) after injection of *Dscam1* dsRNA. (**A**) For both the haemocoelic bacterial and oral bacterial exposure experiments *Dscam1* was down-regulated in larvae four days after the injection of D-ex15 dsRNA (red bars). Whole body [WB], Haemocytes [H] were sampled from a subgroup of animals from the haemocoelic bacterial exposure experiment and WB and Gut samples were sampled from a subgroup of animals from the oral bacterial exposure experiment. (**B**) For the behavioural and fecundity experiment, *Dscam1* was down-regulated in whole body samples of males (♂) and females (♀) 25 days after the injection of D-ex12 (turquoise bars) and D-ex15 (red bars) dsRNA. The expression of the reference genes *Rpl13a* and *Rp49* was used to normalise the expression of the *Dscam1*. Means and standard errors (SE) were calculated with the REST software; n = 3 biological replicates for each bar, each replicate contained 10 animals.
