# Supplementary material for: Down syndrome cell adhesion molecule 1: testing for a role in insect immunity, behaviour and reproduction
Source: R Soc Open Sci. 2016 Apr 20;3(4):160138. doi: 10.1098/rsos.160138 (PMC4852650; doi:10.1098/rsos.160138)
Supplement: Figure S6. Survival of T. castaneum larvae after Dscam1 knockdown and haemocoelic bacterial or oral bacterial exposure. [file rsos160138supp6.pdf]

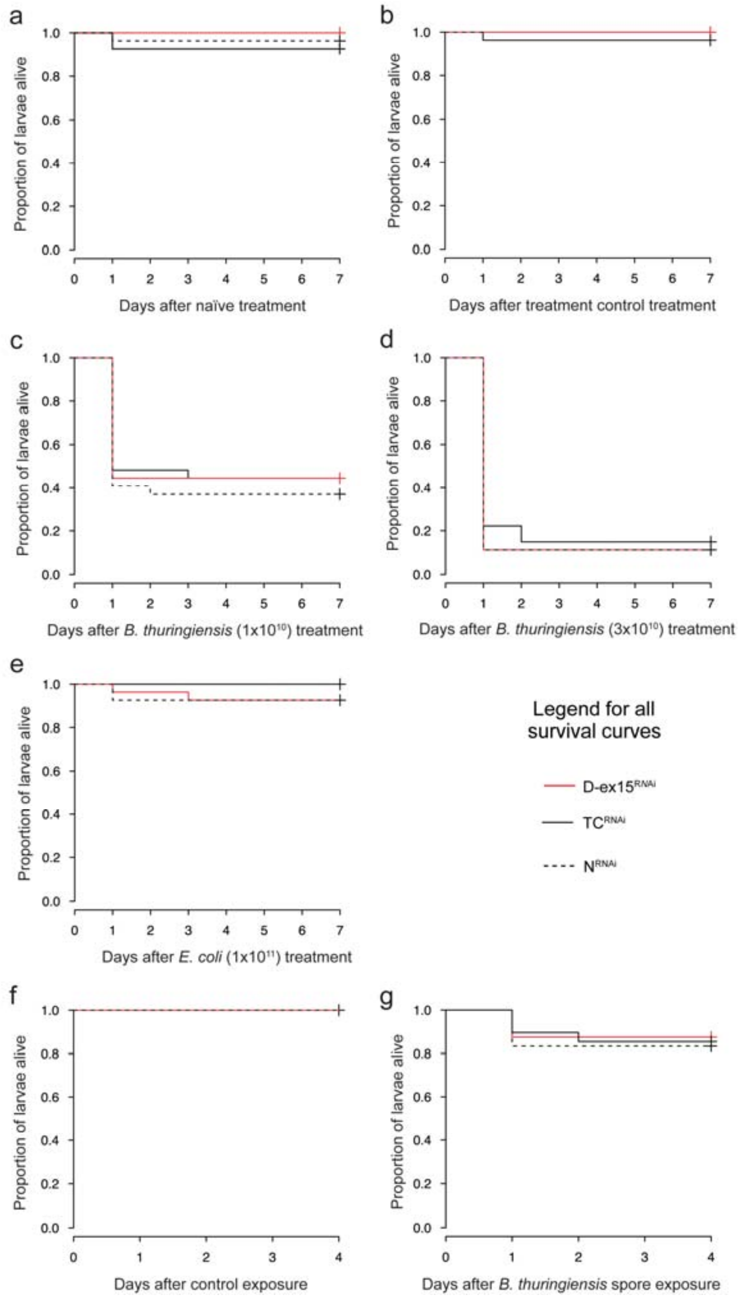


**Figure S6. Survival of *T. castaneum* larvae after *Dscam1* knockdown and haemocoelic bacterial or oral bacterial exposure.** Larvae received either *Dscam1* (D-ex15$^{RNAi}$) knockdown (red line), were injected with a treatment control (TC$^{RNAi}$; black line) or left untreated (N$^{RNAi}$; black dotted line) four days before bacteria exposure. For the haemocoelic bacterial exposure experiments, (**a**) control larvae were left untreated, (**b**) treatment control larvae were pricked, (**c**) and (**d**) larvae were exposed to one of two concentrations of *B. thuringiensis* or (**e**) *E. coli*, and survival was followed for seven days. Each curve is the cumulative survival of 27 individuals. For the oral bacterial exposure, larvae were (**f**) exposed to a control or (**g**) a *B. thuringiensis* spore containing diet. Survival was followed for four days. Each curve is the cumulative survival of 48 individuals. For both infection routes there was no significant interaction between the knockdown and the infection treatment (see main text for details).
