# Supplementary material for: Down syndrome cell adhesion molecule 1: testing for a role in insect immunity, behaviour and reproduction
Source: R Soc Open Sci. 2016 Apr 20;3(4):160138. doi: 10.1098/rsos.160138 (PMC4852650; doi:10.1098/rsos.160138)
Supplement: Figure S7. The proportion of adult T. castaneum found on their backs after larval knockdown of Dscam1. [file rsos160138supp7.pdf]

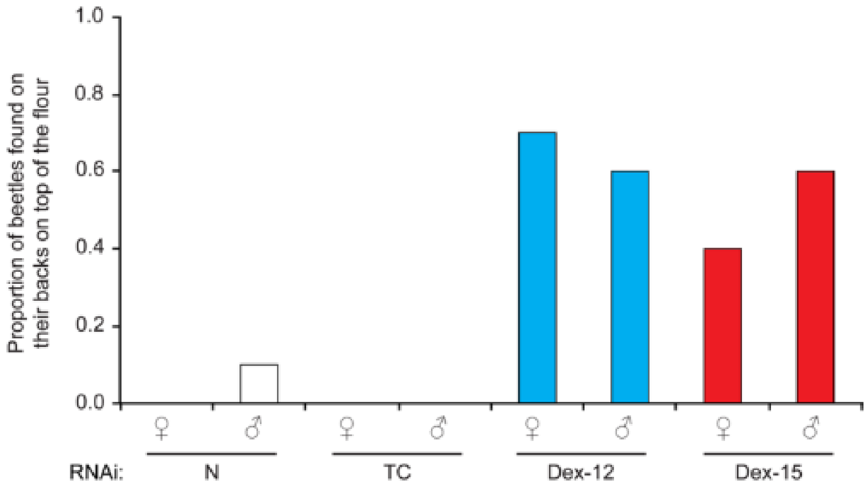

Figure S7. The proportion of adult *T. castaneum* found on their backs after larval knockdown of *Dscam1*. The proportion of beetles (females [♀] and males [♂]) from D-ex12^{RNAi} (turquoise), D-ex15^{RNAi} (red), TC^{RNAi} and Naïve^{RNAi} (white bar) that were positioned on their backs 25 days after dsRNA injection. The proportions were calculated from n = 10 animals for each bar.
