# Supplementary material for: Down syndrome cell adhesion molecule 1: testing for a role in insect immunity, behaviour and reproduction
Source: R Soc Open Sci. 2016 Apr 20;3(4):160138. doi: 10.1098/rsos.160138 (PMC4852650; doi:10.1098/rsos.160138)
Supplement: Figure S8. Climbing and open arena assays of control and TcDscam1 dsRNA-injected adult T. castaneum. [file rsos160138supp8.pdf]

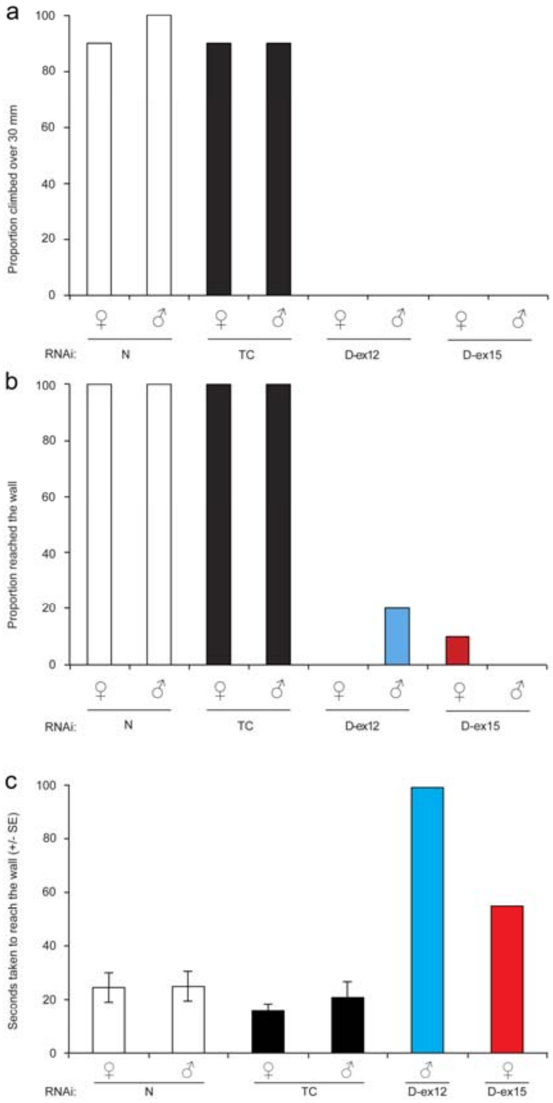

Figure S8. Climbing and open arena assays of control and *TcDscam1* dsRNA-injected adult *T. castaneum*. Assays were performed on 10 females and 10 males for each of the following groups: naïve (N^{RNAi}; white bars), dsRNA treatment control (TC^{RNAi}; black bars) and Dscam1 knockdown ($D\text{-}ex12^{RNAi}$ & $D\text{-}ex15^{RNAi}$; turquoise and red bars, respectively).Climbing assay: (**a**) the ability of the beetles to climb vertically. The proportion of each treatment group that successfully climbed over 30 mm in one minute is shown. Open arena assay: (**b**) Beetles were placed in a circular arena and the proportion that successfully reached the wall in two minutes is shown. (**c**) Mean time that it took control and *TcDscam1* dsRNA-injected adult *T. castaneum* to reach the wall of the arena. All RNAi controls (N [white bars] and TC [black bars]) females and males reached the wall. However, only one $D\text{-}ex15^{RNAi}$ female and two $D\text{-}ex12^{RNAi}$ males reached the wall (no error bar since it took the same time for both males). Because only three *TcDscam1* knockdown beetles reached the wall we were unable to statistically analyse the data for these animals. However, analyses of the controls showed that there was no significant difference in the time it took for males or female ($t = -0.61$, $p = 0.55$) or the naïve or TC beetles to reach the wall (treatment: $t = -1.03$, $p = 0.31$).
