# Supplementary material for: Down syndrome cell adhesion molecule 1: testing for a role in insect immunity, behaviour and reproduction
Source: R Soc Open Sci. 2016 Apr 20;3(4):160138. doi: 10.1098/rsos.160138 (PMC4852650; doi:10.1098/rsos.160138)
Supplement: Figure S9. Morphology of control and TcDscam1 dsRNA-injected female beetles and their reproductive organs. [file rsos160138supp9.pdf]

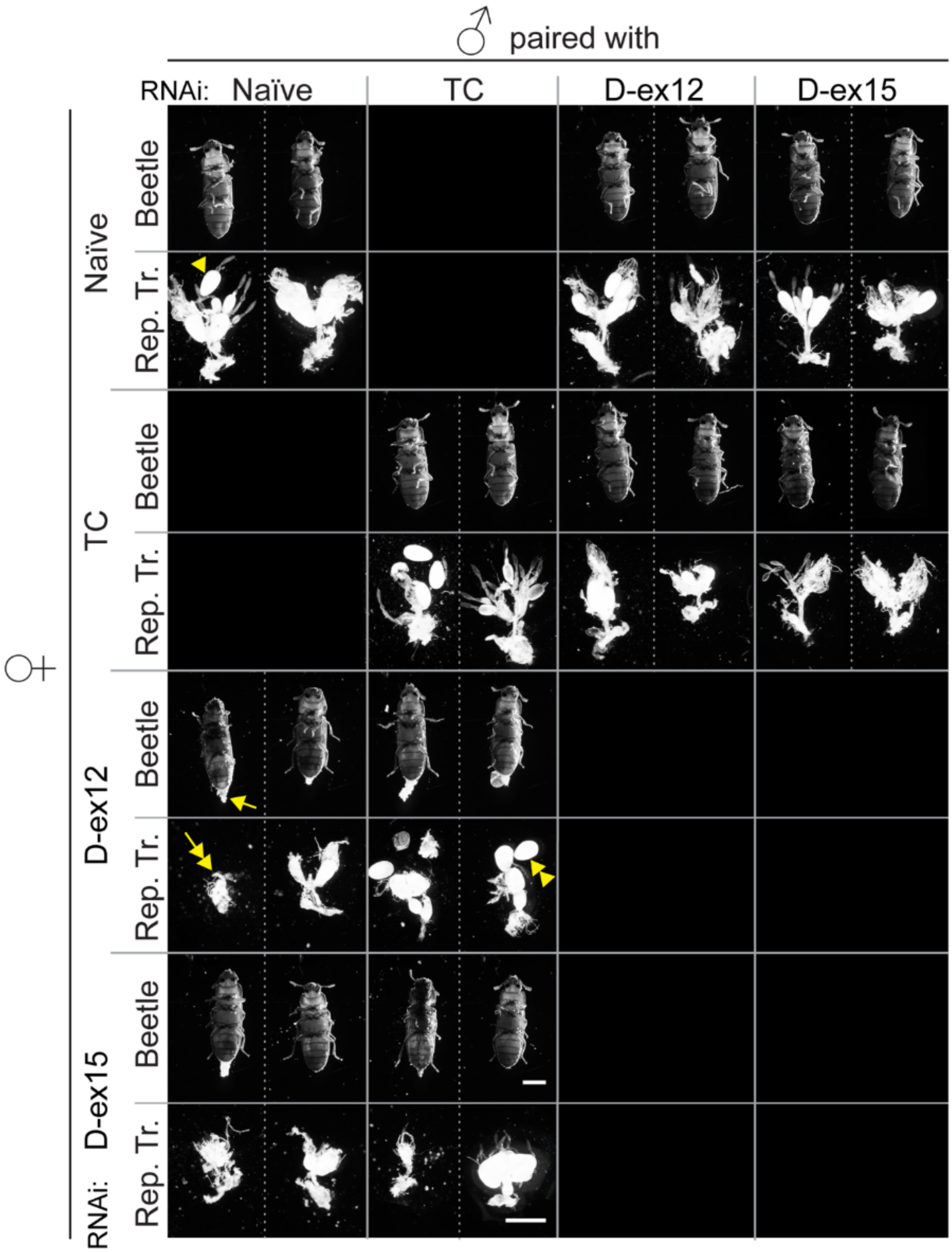

Figure S9. **Morphology of control and *TcDscam1* dsRNA-injected female beetles and their reproductive organs.** The y-axis indicates the treatment group of the female and Rep. Tr. Indicates reproductive tract. The top x-axis indicates the treatment group of the males with which the females had been paired, three days previously. An everted ovipositor is indicated by a single yellow arrow. A normal-shaped egg is indicated by a single yellow arrowhead and a misshapen egg by yellow double arrowheads. Ovaries lacking eggs are indicated by a yellow double arrow. The white scale bar next to the beetle indicates 1 mm and the one next to the reproductive tract 0.5 mm.
