# Supplementary material for: Down syndrome cell adhesion molecule 1: testing for a role in insect immunity, behaviour and reproduction
Source: R Soc Open Sci. 2016 Apr 20;3(4):160138. doi: 10.1098/rsos.160138 (PMC4852650; doi:10.1098/rsos.160138)
Supplement: Figure S10. Fecundity of control and TcDscam1 ds-RNA-injected females from pairings with differently treated males. [file rsos160138supp10.pdf]

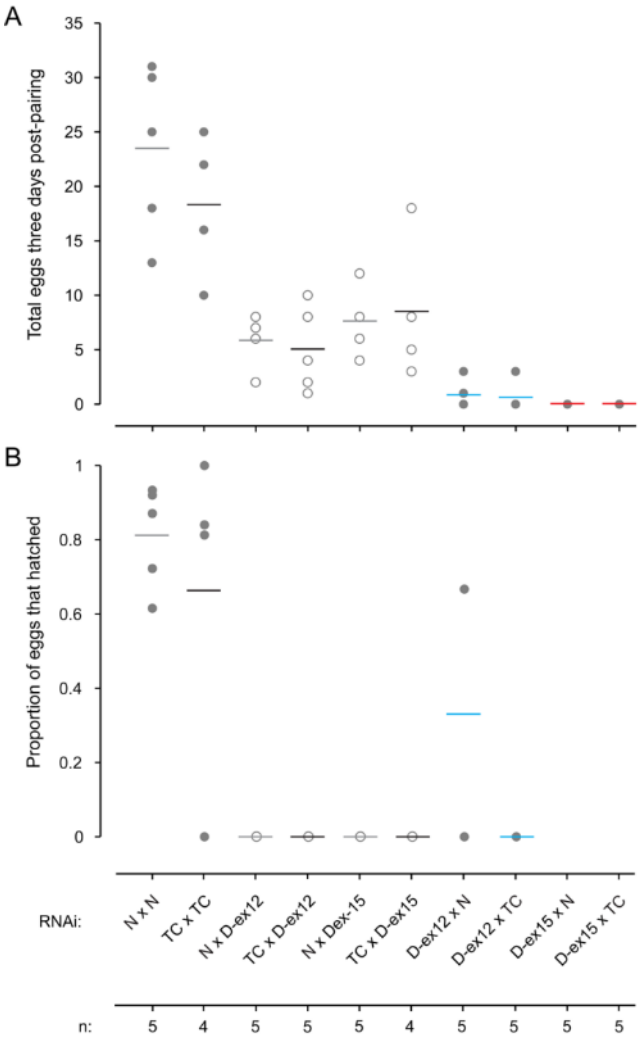

Figure S10. **Fecundity of control and *TcDscam1* ds-RNA-injected females from pairings with differently treated males.** (**A**) The total number of eggs laid over a three-day period following male removal; (**B**) the proportion of eggs that hatched. For both measures, the pairing treatment is indicated beneath the figure with female treatment first; grey-filled circles indicate pairs where copulation was attempted and white-filled circles indicate where no copulation was attempted. Mean values are shown as a grey, black, turquoise or red line, depending on the female RNAi treatment. "n" below the figures indicates the number of pairs.
