# Supplementary material for: Down syndrome cell adhesion molecule 1: testing for a role in insect immunity, behaviour and reproduction
Source: R Soc Open Sci. 2016 Apr 20;3(4):160138. doi: 10.1098/rsos.160138 (PMC4852650; doi:10.1098/rsos.160138)
Supplement: Table S1. Primer sequences. [file rsos160138supp11.pdf]

Table S1. **Primer sequences.** Efficiencies (E) are given for primers that were used for RTqPCR. An arrow within primer sequences indicates where an intron lies within a RTqPCR primer.

Organism	Gene Symbol	Name	Gene Accession Number	Use (E / Fragment length bp)	5' – 3' primer sequence	Primer origin
D. melanogaster	*Dpt*	*Diptericin*	FBgn0004240	RTqPCR (1.979 / 68)	F: GCTGCGCAATCGCTTCTACT<br>R: TGGTGGAGTGGGCTTCATG	(1)
	Drs	*Drosomycin*	FBgn0010381	RTqPCR (1.945 / 79)	F: CTGCCTGTCCGGAAGATACAA<br>R: TCCCTCCTCCTTGCACACA	(2)
	Dscam1	*Down syndrome cell adhesion molecule 1*	FBgn0033159	RTqPCR (2.000 / 105)	F: TAAGGCCTTCGCCCAGGGATCC<br>R: TCTCCGGGGGTGTCGC↓CAACT	(3)
	Imd	*Immune deficiency*	FBgn0013983	RTqPCR (1.954 / 85)	F: TTGGCATGTCGGAAGGACAG<br>R: AACAGCTGGTATATCAC↓CTCTCTAA	This study
	rp49	*Ribosomal protein 49/L32*	FBgn0002626	RTqPCR (2.000 / 97)	F: CGCCCAGCATACAGGCCCAA<br>R: TGCGCCATTTGTG↓CGACAGC	(3)
	rpL13a	*Ribosomal protein L13a*	FBgn0037351	RTqPCR(1.980 / 104)	F: AAGGCAGTCCGAG↓GCATGATCCC<br>R: CGACGCTTGTCGTAGGGCGA	(3)
T. castaneum	*Att2*	*Attacin2*	TC007738	RTqPCR (1.949 / 163)	F: CAAACGACCAAAG↓GGAAACTA<br>R: CTTCCTCCAAGCAAAGTTGG	(4)
	Col1	*Coleoptericin1*	TC005093	RTqPCR (1.972 / 120)	F: TTTGGCACTTTTTGCACTTG<br>R: GGGATGTCCTGTTCTACGGA	(5)
	Dscam1	*Down syndrome cell adhesion molecule 1*	TC012539	RTqPCR (2.000/ 83)	F: AGGGCTACTG↓GGGTTTCACC<br>R: GTAAGAGTGCCGTCTTCGA	This study
	Imd	*Immune deficiency*	TC010851	RTqPCR (1.970 / 129)	F: CCTCCAAGGGATGAAGTCAA<br>R: ACTGGCAAAAG↓CAGATGGTC	(4)
	rp49	*Ribosomal protein 49*	TC006106	RTqPCR (1.955 / 132)	F: TTATGGCAAACTCAAA↓CGCAAC<br>R:GGTAGCATGTGCTTCGTTTTG	(6)
	rpL13a	*Ribosomal protein L13a*	TC013477	RTqPCR (2.000 / 186)	F: GGCCGCAAG↓TTCTGTCAC<br>R:GGTGAATGGAGCCACTTGTT	(4)
	Dscam1	*Down syndrome cell adhesion molecule 1*	TC012539	D-ex12^{RNAi} (NA / 145)	F: CGGCGATTACAAAGATTTCAA<br>R: AACTGCAGATAATCCTGATCCAA	This study
				D-ex15^{RNAi} (NA / 282)	F: CGGAGGCTCCAATCGCCGTT<br>R: TCGACGGTTGCCCTTCTCCA	This study
E. coli	*AsnA*	*Asparagine synthetase A*	ECK3738	TC ^{RNAi} (NA / 304)	F: ATGGTGGCGGCAATACGTGGATC<br>R: GATTACTCCATCGCAGAAGCTGC	This study

1. Romeo Y, Lemaitre B. *Meth. Mol. Biol.* 2008;415:379-94.
2. Fellous S, Lazzaro BP. *Mol. Ecol.* 2011;20(7):1558-67.
3. Armitage SAO et al. *PLoS ONE*. 2014;9(10):e108660-e.
4. Peuß R et al. *Proc R Soc B*. 2015;282(1819).
5. Zou Z et al. *Genome Biol.* 2007;8:R177-R.
6. Konopova B, Jindra M. *Proc. Nat. A. Sci. USA*. 2007;104:10488-93.
