# Supplementary material for: Down syndrome cell adhesion molecule 1: testing for a role in insect immunity, behaviour and reproduction
Source: R Soc Open Sci. 2016 Apr 20;3(4):160138. doi: 10.1098/rsos.160138 (PMC4852650; doi:10.1098/rsos.160138)
Supplement: Table S2. Summary of results of pairing behaviour after Dscam1 knockdown in T. castaneum. [file rsos160138supp12.pdf]

Table S2. Summary of results of pairing behaviour after *Dscam1* knockdown in *T. castaneum*. Female and male columns indicate the treatment of each animal in the pairing. Grey shading indicates a phenotype similar to those pairings where females and males were both N^{RNAi}, i.e., the contol.

Female	Male	Female genital eversion (grey = no)	Male genital eversion (grey = no)	Mating attempt (grey = yes)	Eggs (grey = yes)	Larvae (grey = yes)
N^{RNAi}	N^{RNAi}	grey	grey	grey	grey	grey
N^{RNAi}	N^{RNAi}	grey	grey	grey	grey	grey
N^{RNAi}	N^{RNAi}	grey	grey	grey	grey	grey
N^{RNAi}	N^{RNAi}	grey	grey	grey	grey	grey
N^{RNAi}	N^{RNAi}	grey	grey	grey	grey	grey
TC^{RNAi}	TC^{RNAi}	grey	grey	grey	grey	grey
TC^{RNAi}	TC^{RNAi}	grey	grey	grey	grey	
TC^{RNAi}	TC^{RNAi}	grey	grey	grey	grey	grey
TC^{RNAi}	TC^{RNAi}	grey	grey	grey	grey	grey
N^{RNAi}	D-ex12^{RNAi}	grey	grey		grey	
N^{RNAi}	D-ex12^{RNAi}	grey	grey		grey	
N^{RNAi}	D-ex12^{RNAi}	grey	grey		grey	
N^{RNAi}	D-ex12^{RNAi}	grey			grey	
N^{RNAi}	D-ex12^{RNAi}	grey	grey		grey	
TC^{RNAi}	D-ex12^{RNAi}	grey			grey	
TC^{RNAi}	D-ex12^{RNAi}	grey	grey		grey	
TC^{RNAi}	D-ex12^{RNAi}	grey	grey		grey	
TC^{RNAi}	D-ex12^{RNAi}	grey	grey		grey	
TC^{RNAi}	D-ex12^{RNAi}	grey	grey		grey	
N^{RNAi}	D-ex15^{RNAi}	grey	grey		grey	
N^{RNAi}	D-ex15^{RNAi}	grey	grey		grey	
N^{RNAi}	D-ex15^{RNAi}	grey	grey		grey	
N^{RNAi}	D-ex15^{RNAi}	grey			grey	
N^{RNAi}	D-ex15^{RNAi}	grey			grey	
TC^{RNAi}	D-ex15^{RNAi}	grey	grey		grey	
TC^{RNAi}	D-ex15^{RNAi}	grey	grey		grey	
TC^{RNAi}	D-ex15^{RNAi}	grey	grey		grey	
TC^{RNAi}	D-ex15^{RNAi}	grey	grey		grey	
D-ex12^{RNAi}	N^{RNAi}		grey	grey		
D-ex12^{RNAi}	N^{RNAi}		grey	grey		
D-ex12^{RNAi}	N^{RNAi}		grey	grey	grey	
D-ex12^{RNAi}	N^{RNAi}		grey	grey		
D-ex12^{RNAi}	N^{RNAi}	grey	grey	grey	grey	grey
D-ex12^{RNAi}	TC^{RNAi}		grey	grey		
D-ex12^{RNAi}	TC^{RNAi}		grey	grey	grey	
D-ex12^{RNAi}	TC^{RNAi}		grey	grey		
D-ex12^{RNAi}	TC^{RNAi}		grey	grey		
D-ex12^{RNAi}	TC^{RNAi}		grey	grey		
D-ex15^{RNAi}	N^{RNAi}	grey	grey	grey		
D-ex15^{RNAi}	N^{RNAi}		grey	grey		
D-ex15^{RNAi}	N^{RNAi}		grey	grey		
D-ex15^{RNAi}	N^{RNAi}		grey	grey		
D-ex15^{RNAi}	N^{RNAi}	grey	grey	grey		
D-ex15^{RNAi}	TC^{RNAi}		grey	grey		
D-ex15^{RNAi}	TC^{RNAi}	grey	grey	grey		
D-ex15^{RNAi}	TC^{RNAi}		grey	grey		
D-ex15^{RNAi}	TC^{RNAi}	grey	grey	grey		
D-ex15^{RNAi}	TC^{RNAi}	grey	grey	grey		
