# Supplementary material for: Down syndrome cell adhesion molecule 1: testing for a role in insect immunity, behaviour and reproduction
Source: R Soc Open Sci. 2016 Apr 20;3(4):160138. doi: 10.1098/rsos.160138 (PMC4852650; doi:10.1098/rsos.160138)
Supplement: Supplementary Methods. [file rsos160138supp13.pdf]

Supplementary methods

Study insects and bacteria.

Tribolium castaneum (strain Cro1) were wild-collected in Croatia in 2010 (see 1) and adapted to laboratory conditions for at least 12 generations before the start of the experiments. Beetles were raised on organic wheat flour (Alnatura type 550) with 5 % brewer's yeast at 30 ºC, 70 % humidity, in a 12 hour light/dark cycle. *Drosophila melanogaster* (1_4WS) originated from inseminated females that were wild-collected in Münster, Germany in 2008 and allowed to adapt to laboratory conditions for at least 50 generations before the start of the experiments. These species have been estimated to have last shared a recent common ancestor around 300-327 million years ago (2, 3). Flies were kept on fly food (1.79 % brewer's yeast; 3.57 % malt extract; 7.14% corn flour; 1 % soy flour; 0.4 % sugar beet syrup; 1.07 % agar; 0.14 % Nipagin; 0.4 % propionic acid) at 25ºC, 70 % humidity, in a 12 h light/dark cycle, with non-overlapping generations.

The bacteria used for the infection experiments were *Bacillus thuringiensis morrisoni var. tenebrionis* (*B. thuringiensis*; BGSCID 4AA1) aquired from the *Bacillus* genetic stock center (BGSC), and *Escherichia coli* (DSM no. 498) and *Pseudomonas fluorescens* (DSM no. 50090) which were both acquired from the German collection of microorganisms and cell cultures (DSMZ). For experiments, 50 µl of bacteria glycerol stocks were added to 50 mL of LB medium in a 500 mL baffled Erlenmeyer flask and incubated at 30 ºC, 200 rpm for 15 hours. Bacteria were washed in phosphate buffered saline (PBS, Calbiochem®) at 4 ºC, 2,151 rcf, 15 min) and resuspended with 2 mL PBS and their concentration estimated with a Thoma counting chamber, and the concentration was adjusted as necessary.

Dscam1 *expression in* D. melanogaster *and* T. castaneum*.*

For both insect species, samples for gene expression profiling consisted of three replicate pools of whole body or tissue samples, each containing ten individuals.

Dscam1 *expression in* D. melanogaster.

For *D. melanogaster* we used whole body samples of eggs, first and third instar larvae, pupae, and adults were used. In addition we sampled fat body, haemocytes and brain from third instar larvae and fat body and brain from adults. We also sampled testes and ovaries from adults. To produce the samples, flies (4-5 days post pupal eclosion) were allowed to lay eggs for two hours on agar plates (1.5 % agar; 1 % vinegar) covered in a thin layer of yeast. The eggs were washed off the plates with PBS and three replicates of ten 12-14 hour old eggs were frozen. The flies were allowed to lay eggs for an additional 10 hours, after which time the eggs were washed off the plates with PBS and added to food vials at a constant density (methods after 4) and allowed to develop. Whole body samples of first instar larvae (35-45 hours after egg laying), third instar larvae (120-132 hours after egg laying), pupae (156-168 hours after egg laying), and adults (14 days after egg laying; i.e. around 4 day old adults) were used. The haemolymph from third instar larvae was obtained by carefully pulling animals apart on a glass slide without disrupting the gut. For fat body dissection third instar larvae were pulled apart by the mouth hooks and cuticle and the fat body was separated from other tissues. For adults, the fat body was obtained by removing the abdomen from the body; the gonads were discarded and the fat body tissue was collected into 50 µl of ice-chilled *Drosophila* Ringer's solution (182 mM KCl; 46 mM NaCl; 3 mM $CaCl_2$; 10 mM Tris•HCl; (5)) and shock frozen. Ovary and testis sampling were performed in the same manner as for the fat body but with an additional centrifugation step. All samples were immediately centrifuged after dissection (4 ºC, 23 rcf, 5 min), the supernatant was discarded and the samples were snap frozen in liquid nitrogen and stored at -80 ºC until RNA extraction. All samples from the pupal and adult stages, other than testes and ovaries, contained 5 males and 5 females.

For RNA extraction a tissue specific volume of TRIzol reagent (Ambion, U.S.A) was added to the frozen homogenised tissue samples, incubated for 10 min at room temperature (RT) and vortexed two times during incubation. To ensure complete lysis of the fat body, samples were first incubated with 250 µl TRIzol with an additional shock freezing and sonification step (5-10 min) before adding another 250 µl TRIzol and 10 min incubation at RT. Samples were centrifuged at 4 ºC, 94 rcf for 10 min. The supernatant was transferred to a new tube and mixed with 100 µl of chloroform and incubated for 15 min at RT. Samples were centrifuged at 4 ºC, 9,391 rcf for 15 min. The upper aqueous phase was used to isolate RNA with the SV Total RNA Isolation System (Promega) according to the manufacturer instructions, which included a DNase I digestion step. To optimise RNA concentration, the purified RNA samples were reduced to a volume of approximately 30 µl by vacuum-drying at 45 ºC for 45 min. Samples were stored at -80 ºC until use. For reverse transcription of RNA, SuperScript III™ (Invitrogen) was used according to the manufacturer's instructions using random hexamer primers and a tissue specific amount of RNA as a template.

The resulting cDNA was used for qPCR analyses using gene-specific primers (Table S1). Where genes contained more than one exon, primers were designed such that the forward or reverse primer spanned an intron. Amplification efficiencies (E) of the primer pairs were determined with five dilutions (undiluted, 1:10, 1:100, 1:1,000, 1:10,000) of template cDNA, where $E = 10^{-1/slope}$. The qPCR was performed in a 384-well plate format, with a total reaction volume of 10 µl in each well. From each cDNA sample two technical replicate qPCR reactions were performed using the Kapa SYBR® Fast qPCR Mastermix according to the manufacturer's instructions. The reaction was run on a LightCycler480 (Roche) using the following protocol: 95 °C for 5 min, followed by 40 cycles of annealing and amplification at 60 °C for one min and denaturation at 95ºC for 15 sec. As a final step the products were heated up to 95 °C with continuous fluorescence measurements to obtain the melting curves

and subsequently cooled to 40 °C. The crossing point (Cp) values (see raw data) were calculated using the Fit Point method using a Noise Band threshold of 10, with the LightCycler®480 software and the average Cp values from each of two technical replicates were used for analyses. *Dscam1* expression for each sample was calculated relative to the geometric mean of two reference genes (ribosomal protein 49 (*Rp49*) and ribosomal protein 13a (*RpL13a*)), used in the formula: $E^{(reference)} - E^{(target)} = \Delta ct$. The genes *Rpl13a* and *Rp49* were chosen as reference because they have been found to have relatively stable expression across treatments, life history stages and tissues (e.g., 6, 7-9); however it is important to recognise that it is difficult to find universally suitable reference genes (9). Although across tissues and life history stages, the Ct values of the two reference genes were tightly correlated with each another (*D. melanogaster*: $r^2 = 0.991$, df = 34, $p < 0.0001$; *T. castaneum*: $r^2 = 0.986$, df = 37, $p < 0.0001$), it can be expected that reference genes do not necessarily show identical expression across different tissues and life history stages therefore we discuss the results in terms of expression of *Dscam1* relative to the mean expression of the two reference genes.

Dscam1 *expression in* T. castaneum.

A similar experimental set up was used for *T. castaneum*: whole body samples of eggs, ten and fifteen day-old larvae, three day-old pupae and seven day-old adults were used. In addition we sampled fat body, haemocytes and brain from fifteen day-old larvae and from seven day old adults, and the testes and ovaries from adults. In detail, to produce the samples, two to three week old adult beetles were allowed to lay eggs for 24 hours. Three replicates of ten eggs were collected and frozen, and the remaining eggs were further cultivated as described above. After ten days, larvae were individualised in 96-well microtitre plates containing *ad libitum* flour and 5 % yeast and incubated until sampling. Haemolymph was taken by pricking the larvae and adults with a fine needle (2 - 10 µm Ø) between the head and thorax. Clear haemolymph was collected with 1 µl glass capillaries (Hirschmann, Germany)

and immediately transferred into 50 µl ice-chilled Schneider's insect medium (Sigma) containing 10 % fetal calf serum (FCS). For fat body dissections, larvae were cooled on ice and transferred to a microscope slide with a drop of PBS. Larvae were cut transversally at the first and last segment. The gut was pulled out and the fat body was then squeezed out with forceps and collected in 50 µl ice-chilled PBS. For adult fat body dissection, equal numbers of males and females were cooled and decapitated. The gut and gonads were removed and the fat body was collected in 50 µl ice-chilled PBS. Testes were sampled by cooling the beetle and cutting the abdomen by cutting beneath the last pair of legs on the ventral side. After transfer into a drop of PBS the testes were squeezed out of the abdomen and collected in 50 µl ice-chilled PBS. To dissect ovaries, females were cooled and cut on the ventral side between second and third pair of legs. The abdomen was then cut laterally and transferred into a drop of PBS. Ovaries were push out of the abdomen and transferred into 50 µl ice-chilled PBS. The qPCR was performed in a 96-well plate format, with a total reaction volume of 15 µl in each well. All samples were further treated as described for *D. melanogaster*.

Dscam1 *expression in larval* D. melanogaster *and* T. castaneum *upon infection.*

To address whether there is a change in *Dscam1* expression after bacterial challenge we performed experiments using two different infection routes (e.g., see 10, 11). The first two experiments examined gene expression after haemocoelic bacterial exposure of both *D. melanogaster* and *T. castaneum*. In the third we took advantage of the fact that *B. thuringiensis* can also be orally administered and cause pathogenesis to *T. castaneum* (1) and therefore examined *Dscam1* expression after oral exposure. Infections and dissections were performed blind with respect to treatment as far as possible and treatment orders were randomised within each replicate block.

As a positive control that the immune system had been activated, we additionally tested the expression of three immune genes for each host species. We monitored the expression of *Imd*

and two species-specific antimicrobial peptides (AMPs), *Attacin2* (*Att2*) and *Coleoptericin1* (*Col1*) for *T. castaneum* (12-15) and *Diptericin* (*Dpt*) and *Drosomycin* (*Drs*) for *D. melanogaster* (15-19). In brief, *Imd* plays a signal transduction role in the Imd signalling pathway, this pathway together with the Toll pathway, is responsible for the expression of AMPs after recognition of a bacterial infection (16). *Imd* has been found to be upregulated in *T. castaneum* after infection with some microorganisms (12). In *D. melanogaster* the Imd pathway is activated by Gram-negative bacteria (or Gram-positive bacteria with DAP-type peptidoglycan) and leads to the expression of, e.g., *Dpt*; the Toll pathway is activated by Gram-positive bacteria and fungi and leads to the expression of e.g., *Drs* (19, 20). Both bacteria species we tested, *B. thuringiensis* and *E. coli*, have previously been found to result in increased expression of at least one of our chosen AMPs (e.g., 16, 17). It has been suggested that *T. castaneum* has more promiscuous activation of AMPs than *D. melanogaster* (14), and the AMPs that we chose to test have been shown to be upregulated after infection by bacteria, including *E. coli* (12) *Bacillus subtilis* (14), and another Gram-positive bacteria, *Micrococcus luteus* (12).

Dscam1 *expression after haemocoelic bacterial exposure in larval* T. castaneum.

The experiment consisted of six replicates, each replicate being performed on a different day. Adult beetles were therefore allowed to lay eggs for 24 hour periods over a course of six days. 10 day old *T. castaneum* larvae were individualised in 96-well microtitre plates containing flour and 5 % yeast and incubated as described above. Fifteen day old larvae were pricked dorsolaterally between the second and third segment with a fine needle (2-10 µm Ø) that had previously been dipped into a bacterial suspension or PBS (treatment control, hereafter termed TC), or they were left untreated (naïve control, hereafter termed Naïve). The bacteria concentrations used were: *B. thuringiensis*: 1×10^{10} mL^{-1}; *E. coli*: 1×10^{10} mL^{-1}; *P. fluorescens*: 2×10^{7} mL^{-1} all suspended in PBS. For this and the following *Dscam1* expression after infection experiments we generally aimed to use bacterial concentrations that resulted in

low mortality (typically between 0 and 20 % over three days following exposure). We did not want to create strong selection for a small sub-group of survivors from which we estimated gene expression, yet we wanted to elicit an immune response. The bacterial concentration information was gained from a combination of preliminary experiments and data that we have previously published. In detail, the *B. thuringiensis* concentration for pricking (1 x 10^{10} cells / mL^{-1}) was the same as in (11). Similarly as for *D. melanogaster*, the *E. coli* strain we used is non-pathogenic for *T. castaneum*, therefore we used the same concentration (1 x 10^{10} cells / mL^{-1}) as we used for *D. melanogaster*. The *P. fluorescens* concentration was based upon preliminary experiments showing that 2 x 10^{7} cells / mL^{-1} resulted in approximately 20 % mortality at 3 days post exposure. After pricking, the larvae were put into fresh 96-well microtiter plates containing flour and 5 % yeast. The fat body and haemocytes were sampled six and 18 hours after treatment as described before. We produced six replicates per combination of treatment / tissue / time point, whereby each replicate consisted of tissue from ten pooled animals (6 replicates x 5 treatments x 2 tissues x 2 time points x 10 animals = 1,200 larvae). Survival at six and 18 hours was examined at the time of sampling and we also produced an extra 14 animals per replicate / treatment / tissue / time point to estimate survival at 6, 18 and 24 hours (n = 1,680). Average mortality 6 hrs after pricking was less than 1 % for all five treatment groups; at both 18 and 24 hrs after pricking mortality in the *B. thuringiensis* pricked larvae was ~ 10 %, and for all other treatment groups was it was ~ 3 % or less.

RNA was extracted, cDNA synthesised and qPCRs run as described in before. For each sample, *Dscam1* (Table S1) expression was examined as well as the expression of three immune-related gene: *Attacin2* (*Att2*), *Coleoptericin1* (*Col1*) and *Imd* (Table S1). Cp values were calculated using the Fit Point method with a Noise Band threshold of 3.8844. In some cases for the infection experiments, the melting curves fulfilled our quality criteria (i.e., were above detection limit and had a single clear peak at the expected melting temperature), but the standard deviation (SD) of the Cp value across the two technical replicates was ≥ 0.8. If after

repeating the qPCR the SD was still ≥ 0.8, or if there was no mRNA signal at all (e.g., *T. castanuem*, *Attacin2*, 18 hour haemocyte sample) we removed the sample (gene/tissue/time point/treatment combination). For *D. melanogaster* this was the case for 38 out of 576 samples, and for *T. castaneum* this was the case for 36 out of 720 samples. These removals explain why in Figure 1 there are sometimes less than 6 biological replicates. Of the remaining samples, in some cases the melting curve fell below the detection limit of the LightCycler®480 software. In these cases we re-did the qPCR. If after the re-run the melting curve was still below the detection limit we used one technical qPCR replicate for that biological replicate where the melting curve of the remaining technical replicate met our quality criteria. This was the case for 19 of the remaining 538 samples for *D. melanogaster* and 16 of the remaining 684 samples for *T. castaneum*. We analysed the data using REST© 2009 (relative expression software tool; 21) and compared the gene expression of two groups at a time: in each case the naïve control group was tested against the treatment control or one of the bacteria-exposed groups. REST calculates the relative fold expression differences by using the expression of reference genes (*Rpl13a* and *Rp49*) to normalise the expression levels of the target genes (*Dscam1, Att2, Col1* and *Imd*), whilst taking the reaction efficiency (E) of the PCR into account; it is based on the following formula (21, 22):

$$\text{Relative expression} = E_{target}^{\Delta Cp\ target\ (control\ -\ sample)} / E_{reference}^{\Delta Cp\ reference\ (control\ -\ sample)}$$

A pair wise fixed reallocation randomisation test© is performed to examine whether there are significant differences between the two groups. We allowed 2000 random reallocations of the observed Cp values to the two groups being tested; REST© notes the expression ratio change for each reallocation, and the proportion of these effects gives the p-value assuming a 2-sided test. In our figures we present the mean and standard errors as calculated according to the REST© software, i.e., the results of the 2000 random reallocations.

Dscam1 *expression after haemocoelic bacterial exposure in larval* D. melanogaster. The experiment consisted of six replicates, each replicate being performed on a different day. four to seven days post-eclosion, flies were allowed to lay eggs on agar plates, as described before. Eggs were collected in the morning and evening for six days, and adults were replaced every two days. Prior to bacteria challenge, late second instar *D. melanogaster* larvae were removed from the food vials and briefly rinsed in *Drosophila* Ringer solution and gently dried on a paper towel. Glass capillaries (Hilgenberg GmbH, Germany) that had been pulled to a fine point with a dual-stage glass micropipette puller (PC-10 Narishige) were filled with the bacteria or control solutions and injected into the larvae with a FemtoJet microinjector (Eppendorf AG, Germany). The capillary was inserted laterally towards the posterior end of the larvae at approximately a 20 ° angle. Bacteria suspensions were prepared in *Drosophila* Ringer's solution, diluted 1:10 with a sterile filtered bromophenol blue solution (0.5 mg / mL), such that the final bacterial concentrations were: *B. thuringiensis*: 7.5 x 10^6 ml^{-1}; *E. coli*: 1 x 10^{10} mL^{-1}. The strain of *E. coli* that we used is non-pathogenic to *D. melanogaster*, therefore we could use a relatively high concentration (1 x 10^{10} cells / mL^{-1}) of bacteria to elicit an immune response resulting in low mortality (~ 95 %, 3 days post-injection). *B. thuringiensis* is more pathogenic than *E. coli*, and preliminary experiments showed that 1 x 10^7 cells / mL^{-1} resulted in 20-30 % mortality 3 days post-infection, and 1 x 10^6 cells / mL^{-1} resulted in low mortality, therefore we chose the concentration of 7.5 x 10^6 cells / mL^{-1}. Bromophenol blue was used to check the injection success, since it is not toxic when fed to larvae (e.g., 23) and it did not induce mortality of larvae after injection and did not negatively affect bacteria survival (personal observation). We produced six replicates per combination of treatment / tissue / time point, whereby each replicate consisted of tissue from ten pooled animals (6 replicates x 4 treatments x 2 tissues x 2 time points x 10 animals = 960 larvae). After injection the larvae were placed individually into 0.2 mL PCR reaction tubes containing fly food, recipe as described above. Survival at 6 and 18 hours was examined at the time of

sampling and we also produced an extra 7 or 8 animals per replicate / treatment / tissue / time point to estimate survival at six, 18 and 24 hours (n = 725). Average mortality 6 and 18 hrs after pricking was less than 2 % for all five treatment groups; at 24 hrs after injection mortality in the *B. thuringiensis* exposed larvae was less than 4 %, and for all other treatment groups it was it was 0 %. The methods for RNA extraction, cDNA synthesis and qPCR were the same as for *T. castaneum* except that the immune genes examined were: *Diptericin* (*Dpt*), *Drosomycin* (*Drs*) and *Imd* (Table S1). Relative fold expression calculation and statistical analysis was done as described for *T. castaneum*.

Dscam1 expression after oral infection in larval T. castaneum.

The experiment consisted of six replicates, with two replicates being performed per day. Adult beetles were therefore allowed to lay eggs for 24 hour periods over a course of three days. Seventeen day old *T. castaneum* larvae were exposed to *B. thuringiensis* spore-containing diet or to a control diet. The *B. thuringiensis* spore-containing diet was prepared as described in (1) and using the same concentration. In brief, the spore concentration was adjusted to 1 x 10^9 mL^{-1} with PBS, and 0.15 g of flour with 5 % yeast was added per mL of spores. Forty microlitres of liquid diet per well was pipetted in a 96 well plate; the plates were covered with breathable sealing foil for culture plates (Kisker Biotech) and placed into individual plastic boxes (Curver, New Grand Chef, 2.6 L) with holes in the lids, which were plugged with foam stoppers to allow for air circulation. To dry the diet the boxes were placed at 50 °C for approx. 17 hours. The diet for the control larvae was prepared in the same way, except flour with yeast was mixed with only PBS. Larvae were exposed to spore-containing or naïve diet for three hours and subsequently transferred to 96-well plates containing the diet without spores, at which point the survival was also monitored. Gut samples were taken six and 18 hours after the initial exposure. To do this, larvae were ice anesthetised, the first and the last segments were removed using a scalpel, and a drop of PBS was added to the sample. The gut was carefully pulled out with a pair of forceps and the fat body was removed. The

guts were washed in a droplet of clean PBS, and ten guts were pooled in 1.5 mL centrifuge tubes containing 50 µL of ice-chilled PBS and the tube was frozen in liquid nitrogen. We produced six replicates per combination of treatment and time point, whereby each replicate consisted of ten pooled animals (6 replicates x 2 treatments x 2 time points x 10 animals = 240 larvae). In addition to monitoring survival at the point of transfer from the spore-containing to the spore-free diet, survival at six, 18 and 24 hours was monitored from additional larvae (n = minimum 480). No larvae had died by 3 or 6 hours after exposure, but at 18 hours 5.1 % *B. thuringiensis*-exposed and 0.3 % control larvae had died; additional mortality was noted at 24 hours (total ~ 8 %) in the *B. thuringiensis* group, but there was no further mortality in the control group. Before RNA extraction, the guts were homogenized over liquid nitrogen with a pestle. Further RNA isolation, cDNA synthesis and qPCR was done as described in before, with the addition of immune gene expression (*Att2, Col1,* and *Imd*). Cp values were calculated using Fit Point method with a Noise Band threshold of 6.

Effect of Dscam1 *knockdown on larval* T. castaneum *survival and development after haemocoelic or oral bacterial exposure.*

To address whether there is an effect of *Dscam1* knockdown on survival and developmental time we performed two experiments using different infection routes. The first tested survival and development after haemocoelic bacterial exposure and the second tested survival after oral spore exposure.

Effect of Dscam1 *knockdown on larval* T. castaneum *survival and development after haemocoelic bacterial exposure.*

RNAi is a powerful and well established molecular technique in this species (see 13, 24). To perform knockdown of *Dscam1* in *T. castaneum* larvae we followed the protocol of Posnien *et al.* (25). A non-alternatively spliced region within the *Dscam1* gene, exon 15, was used for RNAi (Figure S1, Table S1; See end of this file for the annotated *T. castaneum Dscam1*

gene), which did not overlap with the *Dscam1* qPCR primer pair. A gene-specific primer pair for *Dscam1* exon 15 (D-ex15) was used to amplify the region of interest from whole body (larval) cDNA. Briefly, RNA extraction and cDNA was produced as described above. To amplify the region of interest we used 1 µL of template cDNA, 10 µL 5x Green GoTaq Flexi Buffer, 26.75 µL water, 5 µL MgCl2 (2.5 mM), 5 µL dNTPs (0.2 mM), 1 µL primers (250 nM) and 0.25 µL GoTaq polymerase (0.5 U) (Promega) and standard PCR conditions (Denaturation: 95°C 2 min; followed by 34 cycles of denaturation: 95 °C 30 sec, annealing: 60 °C 40 sec, and extension: 72 °C 40 sec; final extension: 72 °C 5 min, finally hold at 12 °C). As a treatment control (TC^{RNAi}) for the dsRNA injection we used a 304 bp fragment of the *E. coli* BL21 (DE3) (Invitrogen) gene asparagine synthetase A (*AsnA*; gene accession number ECK3738) The maximum sequence similarity of the fragment with the *T. castaneum* mRNA database is a 16 bp overlap with XM_964818.3 (PREDICTED: *Tribolium castaneum* death-inducer obliterator 1) which is not sufficient to trigger an RNAi effect (26). The TC construct was synthesized in the same way as the knockdown constructs for *Dscam1* with the exception that the gene of interest was directly amplified from a standard colony PCR where we used the following settings: 1 µL of a 1:100 dilution (PBS) from a *E. coli* colony, 10 µL 5x Green GoTaq Flexi Buffer, 26.75 µL water, 5 µL MgCl2 (2.5 mM), 5 µL dNTPs (0.2 mM), 1 µL primers (250 nM) and 0.25 µL GoTaq polymerase (0.5 U) were mixed and standard PCR conditions (Denaturation: 95°C 10 min; followed by 34 cycles of denaturation: 95 °C 30 sec, annealing: 56 °C 40 sec, and extension: 72 °C 40 sec; final extension: 72 °C 5 min, finally hold at 12 °C) were used. The resulting fragments were cloned into *EcoRV* linearized pZErO-2™ vector (Invitrogen) according to manufacturer instructions. Constructs pZErO-D-ex15 and pZErO-TC were used as a template in a PCR with vector-specific primers that flanked the fragment with T7 polymerase promoter sequence at their 5'-and 3'- end. The PCR product was purified with High Pure PCR Cleanup Micro Kit (Roche Diagnostics GmbH, Mannheim) according to the manufacturer's instructions, and approximately 500 ng

of purified product was used for *in-vitro* transcription with MEGAscript high yield transcription kit (Ambion) according to the manufacturer's instructions. Twenty microlitres of reaction mix was incubated for five hours at 37 ºC and precipitated with LiCl (25 µl LiCl, 30 µl H_2O) for one hour at -20 ºC. After centrifugation (10,000 rcf, 30 min, 4 ºC) and washing the pellet with 70 % EtOH, the pellet was air-dried for 20 to 30 min at RT. The dsRNA was resupended in PBS and an annealing step was done where the resuspended dsRNA was incubated at 95 ºC for two min. Immediately after incubation the dsRNA was incubated in pre-boiled water until the water temperature reached 70 ºC, after which followed another incubation at 95 ºC in a thermo block. After five min the thermoblock was removed from the heating source and cooled to room temperature. The dsRNA concentration was measured using NanoDrop (NanoPhotometer ™ Pearl, Implen) and stored at -80 °C until further use.

To produce the experimental animals, two to three week old adult beetles were allowed to lay eggs for 24 hours. Eleven day old larvae were injected with dsRNA (D-ex15^{RNAi}: n = 256; TC^{RNAi}: n = 256; N^{RNAi}: n = 256) as described in (25). Briefly, the concentration of dsRNA was 2.7 µg/µl for D-ex15^{RNAi} and 2.5 µg/µl for TC^{RNAi}. To inject the dsRNA we used glass capillaries that had been pulled with a micropipette puller (as mentioned before). Capillaries were filled with 10 µl dsRNA solution and approximately 300 nl of each dsRNA (ca. 0.8 - 0.9 µg per larvae as recommended by Posien *et al.* (25)) was dorsolaterally injected between the first and second larval abdominal segment using a FemtoJet microinjector. Injected and naïve beetles were individualised into 96-well plates with flour and 5 % yeast. To test the efficiency of the knockdown in the haemocytes and the whole body, we sampled haemocytes from 2 x 10 animals and 2 x 10 whole body larvae from each injection group (D-ex15^{RNAi} and TC^{RNAi}) four days after dsRNA injection. *Dscam1* expression was examined for all samples by using qPCR as described before, using *Rp49* and *RpL13a* as reference genes and TC animals as a control group.

Four days after the dsRNA injections, the mortality was as follows: N^{RNAi}: 4 %; TC^{RNAi}: 10 %; D-ex15^{RNAi}: 7 %. Twenty-seven larvae from each group were randomly assigned to one of the following bacteria treatments: *B. thuringiensis* (1x10^{10} cells / mL^{-1}), *B. thuringiensis* (3 x 10^{10} cells / mL^{-1}), *E. coli* (1 x 10^{11} cells / mL^{-1}), wounding control (TC) or naïve (N) groups. The *B. thuringiensis* concentration for pricking was 1 x 10^{10} (as in 11) and 3 x 10^{10} cells / mL^{-1} to increase mortality and potentially any differences between the knockdown treatments. The *E. coli* concentration was 1 x 10^{11} cells / mL^{-1} as used in (27). Survival and development were monitored for seven consecutive days with an additional check fourteen days post infection; by this later check only two larvae (one *E. coli*-challenged D-ex15^{RNAi} and one *E. coli*-challenge TC^{RNAi}) had died since the seven day check. Larval survival over seven days following haemocoelic bacterial exposure was analysed using the R statistical package (RStudio version 0.99.441) for Macintosh. Within R we used mixed-effects Cox models (packages required: 'coxme' (28), 'survival' (29, 30), 'nlme' (31)'bdsmatrix' (32) and 'Matrix' (33)), which allows for the inclusion of random effects. The model was fitted with day of death as the response variable: larvae that were alive at the end of the experiment were included as censored cases. The fixed effects were the knockdown treatment, the infection treatment and the interaction term between these two. Plate was included as a random factor, however it was removed during stepwise elimination of non-significant terms and the final model with only the fixed effects was tested using Cox proportional hazards. There were three groups where no larvae died (TC^{RNAi} – *E. coli*, D-ex15^{RNAi} – Naïve, D-ex15^{RNAi} – TC (knockdown – infection treatment)); because there was no event, there was no contribution to the likelihood, and a Cox proportional hazards model could not be fitted. Therefore we denoted one larva as dead in each of these groups allowing us to fit the model. Because the full Cox proportional hazards model did not fulfil the assumptions of proportional hazards over time (based on Schoenfeld residuals) we also tested the model using the survival regression (survreg) function in R. The results from the Cox proportional hazards and survival

regression did not qualitatively differ. Figures are presented without the additional dead larvae. Development time to pupa and adult was analysed using JMP version 9.0.0 for Macintosh OS X. One *Dscam1 – E. coli* individual remained as a pupa over the course of the experiment so we removed it from the analyses. When individuals died, they died as larvae, except for one D-ex15^{RNAi} – *E. coli* individual, which died as a pupa. The data could not be transformed to a normal distribution so we performed non-parametric statistics. First we examined whether there was a significant effect of plate on the time to become a pupa or an adult. There was no significant effect of plate on the time to become either a pupa or an adult (pupa: Chi-square = 10.19, df = 7, p = 0.178; adult: Chi-square = 11.28, df = 7, p = 0.127). The data set was then split by infection treatment and we separately tested, within infection treatment, whether there was an effect of the knockdown on the time to become a pupa or an adult. The only case in which the developmental speed was affected by the knockdown was the time after the infection treatment that it took the uninjected naïve control (N^{RNAi}) animals to become adults (Chi-square = 6.20, df = 2, p = 0.045), where the treatment control for the RNAi (TC^{RNAi}) animals took significantly longer than the N^{RNAi} ones to develop (z = -2.51, p = 0.0226; mean development ± 1 s.e. N^{RNAi} = 11.7 ± 0.23 days, TC^{RNAi} = 12.8 ± 0.39 days, D-ex15^{RNAi} = 13 ± 0.56 days).

Effect of Dscam1 *knockdown on larval* T. castaneum *survival after oral bacterial exposure.*

To test survival after oral infection with *B. thuringiensis* in *Dscam1* knockdown *T. castaneum* larvae, we injected 11 day old larvae with dsRNA (D-ex15^{RNAi}: n = 192; TC^{RNAi}: n = 192; N^{RNAi}: n = 192). The concentration of dsRNA was 2.6 µg/µl for D-ex15^{RNAi} and 2.9 µg/µl for TC^{RNAi}. Injections and preparation of the dsRNA were performed as described before. The efficiency of the knockdown was tested by dissecting three pools of five guts, as described before, and by pooling 3 x 10 whole body larvae from the D-ex15^{RNAi} and TC^{RNAi} injected

groups four days after dsRNA injection. *Dscam1* expression was examined for all samples by using qPCR as described before.

Four days after the dsRNA injections we checked larval survival from a subset of injected larvae (mortality: N^{RNAi}: 6 %; TC^{RNAi}: 17 %; D-ex15^{RNAi}: 16 %), and from each injection group 48 size-selected (34) larvae were randomly assigned to a *B. thuringiensis* spore-containing diet and 48 to a naïve non-spore containing diet. The oral infections were performed as described before with the exception that the spore concentration for this experiment was higher 5 x 10^9 mL^{-1}, so that we would induce a higher mortality. Larvae were exposed to spore-containing and naïve discs such that 16 larvae from each treatment were assigned to one infection or control 96-well plate. This was replicated three times resulting in a sample size of 48 larvae per treatment group. Survival was monitored for 4 consecutive days. Larval survival after exposure to spores was analysed as described before. None of the larvae from the N^{RNAi}, TC^{RNAi} or D-ex15^{RNAi} groups exposed to only the spore-free diet died; because there was no event, there was no contribution to the likelihood, and a Cox mixed effect model could not be fitted. Therefore we denoted one larva as dead in each of these groups allowing us to fit the model. The fixed effect was knockdown treatment and plate was included as a random factor. However, the latter was removed from the model as it was not statistically significant and the final model with only the fixed effect was tested using Cox proportional hazards.

Life history effects of Dscam1 *knockdown in* T. castaneum.

To examine whether there is a fitness effect, measured via fecundity, and an adult behavioural phenotype after *Dscam1* knockdown we performed two simultaneous experiments. We then performed a third experiment to further investigate aspects of fecundity. Unfertilised *T. castaneum* females will lay eggs (35), therefore we could examine whether knockdown affects egg production even when the mating was unsuccessful. *T. castaneum* is highly

polygamous – males can mate with up to seven different virgin females in 15 minutes (36). Males reach sexual maturity approximately two days after imaginal eclosion and females after four days (35); furthermore, copulations are brief, Edvardsson and Arnqvist (37) found that on average they last for around 100 seconds. Therefore they make an ideal model with which to examine questions relating to copulation and mating success.

Experimental animals were produced as described before. For these experiments we produced a second *Dscam1* knockdown treatment using exon 12 (D-ex12^{RNAi}; Table S1; Figure S1), the dsRNA was produced as described before. We injected 11 day old larvae with dsRNA (D-ex12^{RNAi}: n = 144; D-ex15^{RNAi}: n = 144; TC^{RNAi}: n = 192; N^{RNAi} (for behavioural assays only): n = 96). The concentration of all dsRNA constructs was 2.7 µg/µl. After injections the larvae were individualised in 96-well plates containing flour and 5 % yeast. After four days, larval survival was checked (mortality: N^{RNAi}: 0 %, TC^{RNAi}: 12 %; D-ex12^{RNAi}: 13 %; D-ex15^{RNAi}: 15 %) and the surviving larvae were transferred into individual glass vials (ø 12.7 mm and 40 mm height with 10 mg of flour plus 5 % yeast) to make it easier to monitor development. Eleven days after the knockdown the pupae were sexed. Twenty-five days post-knockdown the beetles were either used in the behavioural or fecundity assays; only beetles that had been adults for a minimum of three days were used (see 38). Beetles that had not reached the adult stage by 22 days post-knockdown were excluded from the experiment (two individuals from D-ex12^{RNAi}). Mortality between four days post-dsRNA injection and twenty-five days post-dsRNA was as follows: N^{RNAi}: 5 %; TC^{RNAi}: 1 %; D-ex12^{RNAi}: 10 %; D-ex15^{RNAi}: 4 %.

Behavioural tests.

Twenty five days post-knockdown we used ten males and ten females from each injection group for the behavioural assays (n total = 80). On the same day, for each of the four treatment groups, we froze three pools of two female and three pools of two male beetles for

later qPCR to check the knockdown (methods as described before), except for D-ex12^{RNAi}, where we froze two pools of females and four pools of males. The assays were performed between 09:30 and 17:00 (daylight hours for the beetle) at room temperature and under light conditions. The assays were carried out in ten blocks, where each block contained one male and one female of each of the treatment groups, i.e. eight beetles, processed in a random order with respect to treatment and sex within each block. We noticed that at the time when beetles were removed from their glass vials for the first behavioural assay that some beetles were positioned ventral side down and others were dorsal side down in the flour, we therefore noted for each beetle whether this was the case or not. When given the opportunity, *T. castaneum* has an innate response to climb. We therefore adapted an assay from Michalczyk *et al.* (39) to test the speed at which beetles climb vertically. The beetles were removed from their glass vials and placed on their backs in individual glass Petri dishes. The beetle was passed blind with respect to treatment to a second experimenter who offered the beetle a white strip of paper (2.5 mm wide x 100 mm long, with a pencil mark at 30 mm), such that the bottom edge of the paper was in contact with the beetle's tarsi. When the beetle gripped onto the paper, the paper was lifted up from the Petri dish by approximately 10 cm. We measured the time that it took for a beetle to completely pass a 30 mm mark (approximately 10 body lengths), if it managed this it was deemed to have successfully climbed. If the beetle gripped onto the paper and did not climb it was deemed to have successfully gripped (all beetles managed this). If the beetle had not passed the 30 mm mark after one minute the observation was stopped. The beetle was replaced into its original glass vial containing flour and yeast and left at room temperature for 30 min (e.g., as in 40), before a second behavioural test. The glass Petri dishes were wiped with 70 % EtOH between trials (e.g., as in 40).

Behavioural studies in *T. castaneum* have shown that adult beetles are attracted by tall dark shapes (41). By placing the beetles in an open arena surrounded by a dark-coloured wall, we were able to test locomotion ability with respect to the time beetles needed to reach the wall.

To investigate beetle behaviour in an “unprotected environment” (40), the beetle was taken from its glass vial and placed in the centre of an open arena (diameter: 195 mm) surrounded by a dark plastic wall (height: 51 mm). A clean glass vial was placed over the top of the beetle and it was allowed to acclimate for one minute. The glass vial was then removed and the time it took until when the beetle reached the wall (40) was noted; if it reached the wall it was deemed a successful trial. If the beetle had not reached the wall after two min the trial was stopped (unsuccessful trial). In all of the behavioural experiments, except for one, there were groups in which there was no variation in the response variable making it not possible to estimate where significant effects lie. Therefore we only describe these results. It was, however, possible to test whether there was an effect of sex or treatment, or an interaction between the two, on the time that it took the TC^{RNAi} and the N^{RNAi} beetles to reach the wall. The response variable, time, was Box-Cox transformed to achieve a normal distribution within each of the treatment groups. We positioned a Canon EOS 5D Mark II camera directly above the arena and filmed representative beetles from each of the knockdown treatments.

Fecundity tests.

On the same day as the behavioural assays we set up female-male pairs in seven different pairings, with twenty pairs per pairing: TC males and females were set up with one another (TC^{RNAi} x TC^{RNAi}) and with males and females of both of the *Dscam1* knockdown groups (female first: TC^{RNAi} x D-ex12^{RNAi}; TC^{RNAi} x D-ex15^{RNAi}; D-ex12^{RNAi} x TC^{RNAi}; D-ex15^{RNAi} x TC^{RNAi}), and we also set up males and females from the *Dscam1* knockdown treatment with the same treatment (D-ex12^{RNAi} x D-ex12^{RNAi}; D-ex15^{RNAi} x D-ex15^{RNAi}). Because of mortality in the D-ex12^{RNAi} female group, four beetles from the behavioural assay were also used for the fecundity assay. The pairs were kept in plastic vials with 4 g of pre-sieved flour plus 5 % yeast in controlled conditions as described above. The pairs were sieved every three days (e.g., 42) for a total of four sieves. Each time we counted the number of eggs that had been laid and put them back into flour plus 5 % yeast. Female survival was noted and the

pairs were placed in new vials with fresh flour plus 5 % yeast. If the male died we allowed the female to continue laying eggs until the end of the experiment. Ten days after each egg count we counted the number of larvae that had hatched. This is the first study to use this wild type stock population, Cro1, in mating assays, and as such we note that the hatching rate of eggs (~ 80 %) estimated from control (TC^{RNAi} x TC^{RNAi}) pairings is comparable to wild type *T. castaneum* hatching rates that have been found in other populations (35, 43). Egg cannibalism is known to occur in adult *T. castaneum* (e.g., 35, 44) therefore we cannot exclude the possibility that it might have occurred during the 12-day adult pairing period and affected some of our egg counts. We removed the eggs from the adult pair every three days (the same frequency was used for fecundity analyses by, e.g., 42), meaning that the resulting cohorts of developing larvae were synchronised, which is important because older larvae have been found to have higher egg cannibalism rates than younger larvae (45).

Female survival was analysed as described above, using Cox proportional hazards. The model was fitted with day of death as the response variable. The fixed effect was the combination of the female and male knockdown treatments giving a seven-level factor). There were no female deaths in the TC^{RNAi} x D-ex12^{RNAi} group, therefore for the same rationale as given above, one female was denoted dead in order to run the model. Because some beetles died during the course of the fecundity experiment, to analyse the total number of eggs over the four sieves we excluded any pairs where either the female or the male had died; this resulted in the following numbers of pairs being included in the analysis: TC^{RNAi} x TC^{RNAi} = 18; TC^{RNAi} x D-ex12^{RNAi} = 17; TC^{RNAi} x D-ex15^{RNAi} = 17; ; D-ex12^{RNAi} x TC^{RNAi} = 7; D-ex12^{RNAi} x D-ex12^{RNAi} = 10; D-ex15^{RNAi} x TC^{RNAi} = 6; D-ex15^{RNAi} x D-ex15^{RNAi} = 11. Only nine (26 %) of the females from either of the *Dscam1* (D-ex12^{RNAi}, D-ex15^{RNAi}) knockdown groups laid eggs, we therefore only tested whether the *TC* females differed significantly in the number of eggs that they laid depending upon who they had been paired with. The data were normally distributed and had equal variances so we performed an ANOVA using JMP. Apart

from two D-ex12^{RNAi} knockdown females paired with TC males, larvae only hatched from the pairings between TC^{RNAi} treated females and males, we therefore did not statistically analyse this data set.

Mating behaviour and physiology.

In the fecundity experiment above the females and males were placed together continuously and no observations were made of mating behaviour. In this experiment we therefore aimed to examine whether knockdown females and males mate and also whether female knockdown beetles show evidence of reduced ovaries. Experimental animals were produced as described before. We injected 11 day old larvae with dsRNA (D-ex12^{RNAi}: n = 72; D-ex15^{RNAi}: n = 72; TC^{RNAi}: n = 72; N^{RNAi}: n = 72). The concentration of dsRNA constructs was 2.4 µg/µl for D-ex12^{RNAi} and D-ex15^{RNAi}, and 2 µg/µl for TC^{RNAi}. After injections the larvae were individualised in 96-well plates containing flour and 5 % yeast. After four days, larval survival was checked (mortality: N^{RNAi}: 1 %, TC^{RNAi}: 21 %; D-ex12^{RNAi}: 10 %; D-ex15^{RNAi}: 14 %) and the surviving larvae were transferred into individual glass vials (ø 12.7 mm and 40 mm height with 10 mg of flour plus 5 % yeast) to make it easier to monitor development. Eleven days after the knockdown the pupae were sexed. Twenty-eight days post-knockdown the beetles were assayed; only beetles that had been adults for a minimum of six days were used. Mortality between four days post-dsRNA injection and twenty-eight days post ds-RNA was as follows: N^{RNAi}: 8 %, TC^{RNAi}: 20 %; D-ex12^{RNAi}: 47 %; D-ex15^{RNAi}: 25 %.

We set up females and males in ten different pairing combinations. As a full control, we paired TC^{RNAi} x TC^{RNAi}. We additionally included N^{RNAi} x N^{RNAi}. We also set up all combinations of TC^{RNAi} and N^{RNAi} females and males with both knockdown females and males, i.e., female first: TC^{RNAi} x D-ex12^{RNAi}; TC^{RNAi} x D-ex15^{RNAi}; D-ex12^{RNAi} x TC^{RNAi}; D-ex15^{RNAi} x TC^{RNAi}; N^{RNAi} x D-ex12^{RNAi}; N^{RNAi} x D-ex15^{RNAi}; D-ex12^{RNAi} x N^{RNAi}; D-ex15^{RNAi} x N^{RNAi}. Because in the previous experiment D-ex12^{RNAi} x D-ex12^{RNAi} and D-ex15^{RNAi} x D-

ex15^{RNAi} did not produce eggs we did not include these combinations. All pairings were set up in five replicates, except for TC^{RNAi} x TC^{RNAi} and TC^{RNAi} x D-ex15^{RNAi} where we had four replicates each. The assays were performed between 11:00 and 18:00 (daylight hours for the beetle) at room temperature and under light conditions. In a previous experiment we had observed that some knockdown females had an everted ovipositor, this phenotype has also since been detailed on ibeetle (46), therefore prior to pairing we checked all females and males under a dissecting microscope for everted genitalia. Females were allowed four minutes to acclimatise to a plastic Petri dish (3.5 cm diameter), where the bottom surface had been thoroughly scratched to provide a rough surface for the beetles to right themselves if they fell over. After four minutes we added the male and noted the time at which he had been added. We noted the time at which the male appeared to start copulating with the female (when he mounted her and it looked as if genital contact had been made), and also the time at which the pairing ended. An attempted copulation was defined as an interaction where genital contact appeared to be maintained for at least 35 seconds: Edvardsson & Arnqvist (37) found that male *T. castaneum* with a copulation duration of shorter than 36 seconds did not father any offspring. The mating durations that we found in this experiment for our wild-type (mean duration in seconds ± 1 S.E.: N^{RNAi} x N^{RNAi} = 207 ± 113; TC^{RNAi} x TC^{RNAi} = 134 ± 13) are comparable to previous findings from other wild-type *T. castaneum* populations e.g. (37, 47). If directly after copulation the male had no immediate further interest in the female we separated the pair. However, if the pair separated and the male immediately had further interest in the female, by attempting to mount her and rubbing the lateral edges of her elytra with his tarsi (37), we left the pair together. In these cases where there were multiple attempts by the male to mate the female, we observed them until there was a 10 minute period with no interaction and then separated them. With the exception of one TC^{RNAi} x TC^{RNAi} pairing that had two mating attempts, multiple attempts to mate always involved a knockdown female paired with a TC^{RNAi} or N^{RNAi} male. The pairs were given a maximum of one hour in which to

mate, after this time they were separated and females were placed in individual plastic vials containing 4 g of pre-sieved flour plus 5 % yeast and kept in controlled conditions as described above. The flour was sieved after three days and the number of eggs was counted. The eggs were kept for ten days and after this time the numbers of larvae were counted. The ovaries of twenty females were dissected on the same day that the eggs were counted; they were then photographed with a Canon EOS 5D Mark II under a dissecting microscope. We present the data from this experiment as descriptive because the responses measured were mostly binary and because of the relatively low sample sizes.

On the same day as the pairs were set up, five males and five females from each of the four treatment groups were placed individually into a thin glass tube (100 mm x 5 mm) with a strip of paper (width 3.5 mm) lining the bottom. The last 30 mm of the tube were covered with a piece of black cotton. We positioned a Canon EOS 5D Mark II camera with a macro lens to the side of the glass tube to film the beetles, thus providing some lateral footage of the beetles' movements as they walked, and we left the beetles inside the tube for a maximum of one minute and noted whether or not they reached the darkened area of the tube.

Annotation of the *TcDscam1* gene

Date accessed website: 11.09.14
Official *Dscam1* Annotation from Tcas4 data (beetlebase & aug project)
Found here: http://bioinf.uni-greifswald.de/gb2/gbrowse_details/tcas4?name=TC012539

For alternatively spliced exons 4 and 6 we follow the exon numbering from (48-50). In the below annotation there are 3 exons after exon 6, therefore we call the third alternatively spliced region exon 10.

```
ATGTTCCGTG CGATAATCGT GACTTTGCTC GTCCTCGGAG CGTCCGCCGA GGACGACACC TCCGGGCCCG TGTTCGTGCG
CGAGCCGCCC AACCGCATCG ACTTCTCCAA CACCACCGGA GCGGTGGTGG AGTGCAGCGC CCACGGCAAC CCCACGCCGG
ACATCATCTG GGTCAGGTCG GATGGGACCG CCGTCGGCGA CGTCCCCGGA CTGCGCCAAG TGCTCGCCAA TGGAAATCTG
GTGTTTCCGC CGTTCAGGGC CGAGGACTAC CGCCAGGAGG TGCACGCGCA GGTCTACGTC TGTCTGGCGA AGAACAGCGT
GGGCTCGGTG CATTCGAGGG ACGTCAACGT GAGGGCAGGT TGGTGGAACG CGCCGGAAAA GCGATGGGCA ATAATGGAAA
TGGGGGCCGG GCACGGAACT GCATATTTAT GAATTGTGGG ACAGAAACAA GACGGATGAT AATAATATAC AGAACAGAGT
CTCATTTTTT ATTGAATTCG AATACCTAAA TAACAACAGT AAAAGTGATA ACTGATAAAG CAAGCAATTT TCGTATAAGA
CATTTAAAAA AGTACCAAAT GCAAAAATCA TTCACTTGAT CAGTGTTTTA AATGCTGAGT AGGACTTACT ACGGTGTAAT
TTTCAGTTTA AGTATTTTTA ACTAATATTC ACACAGAAAT CAGCGTTTGC CATTAATTAA GTACTGATTT TTTTTTCTCT
AGGCATGTGA TTTTTTCCAG AATTTTTCAA TAATCCTTTA TAATAAATAC AAACTGGCCG CACAAAATTT ATATCTTTGT
TATGAGTCAT TTTTCAAAAA ATCCTTGAAT TTTTTTATGT TTTATAAGTA TTTTTTCCAA AACAGATATC TAGTTTCATT
TTATTTTGAA TTTATACAAA AAGGCAGGAA GTAAGTCCTC TGATACATTA TTAACAGAAC GATTAAGATA GAACTGATAA
CTTAGGTATT CCTAATAAGT AATAAGCAAC GTTACAGGAA TAAAATCATT GCCCTGTCTA ATGAATTTAA TAACACAAAA
CTGCATTGTA AAATAATAAT TGTCATCATT ATTTGTAGAA AAAGGAATCA TATTGACAAA AGTAAAATAA AGTTAAAACT
GAAGTTAAGA CAAACTTTAA AAACGCAACC AGGATGTTTC CTGGTTTACG ATAATAATAA AAATAATAAT TTATTAATCA
GGTAAGGTAG GTATGTAGTA AAAATCAAAT TGTTTCAAAA ATTAAGCAAA AATCAGAATG AAAGTTCAAC TTTCCTATAA
ATACAACAGC GTTTATAGTT AAAAAATGAC TTAACGCTTT TAAAAAAATA TTATAATTTT GACTAAATTT AGAGATTTTT
TCGAACGATA ACAAAATTTC GGAAAAAATA AAAAATTTTC GCGTTATTTT TGACGAAGTA TTGCGAGTGA TGGCGTGTTT
CAGTTAGCTT TAGAAAAACT TTGTAAGAAA ACAGATGATT TTAGCTCGAA AAATCATATG CCATCATAGA GTTAGTGTTT
TTTTTAACTA GTTTTGAAAA ATTTTGCAAA TGAAATAAGT GATGGTGGTG TTTCTGGATA AAAAAAGTAG TAAAATGTTC
AATAATGTTT TTTTTTATAA TGACTTAGTC ATTTTTGGTG AAAATGAATA GGTGTTCAAA AACTAAACAA AATTGTGCAT
ATGATTTAAT TAGGTACTGG CAGAGGTAAG ACAAAAGGGT AGTCATGAAA ACTTATTAAT AAATTGCGAA TTTAAGAAAA
TTTTGAATGA CAATAAATTA GAAACGGTTA GTTAAAAAAA GCTCAATTAT AGACGAAACA GACATGATTC AGTAAAAAAT
ACCCTAAATG ATTCTTTACA TACCATTTTT TCAAAAGTTA TTAATTTATT ATATTTTTAT TATAAATTAA AGTTATCAAT
AAACCACAGA TAAAAATTGT GTTTAGTTTA ATAAAACTAG ATTTTTTATT AATTAAACAA AAAACCAGAG TAAAGTTCAA
CTACTCAAAT CATTTCATAG GTATTTTAAT ATTATATTAC ATAGTTATTT TAAAAATAGG ACGAAATCTT TAACGTTATT
GGAATAACTT GAAATAAAAA AGACAATTTT GGTTGGTTTT TTCTTACGGT GAAAACTAAA TTCAAGTAAG TTGATATAAT
TTAATGTTCT ATAAAGTATT CTTTAATGTA AATACAAATT AAATCGATTT TATTATTTGT TTATGTTTTT TTCCGAGCCT
TAAATCTTAA GGACTTGCTA CTGAAAACAG ACGACTACTC GTTGAACTAC GAGTTTTACG AATTTTGTCA AATTCTCATT
TAGGATGTTC ACAAACTGAA TTTCAGGAAG TCGGACTATA TTTTGACTTT TTGTTTATCC TGTTTTCCTA AAATTAGCTG
AAATTGAAGT AAGGAACAGC ATCTGGTTCG CATTGTAAAT GTTAGTTTTG ACAAGTCTTA GGTAATTACA GAATGATCGC
TGTAGTTTCA ACTCGAAAAT GAAAAAAATA CAAAATAATG GAAAAAACAA AGAAATAAAG TGCATAATAA ATGCAACACA
CAATGGTTTT TTCAGTCTTT AACAGTTAAA CACTGGCCGA TAATTTACTT TTTAATAATG CTTTGCTTTA TTAAGCACTT
CAAATGTGGT GTAAATAAAC AAAATAATAA ATAAAACAGT TTAAGTAACG TAAAATGTTC ATTAGCTATG AATTTGAATT
TTTTGTTTAT TTTTACGAAA TTCTTTGTAA ATCCCAGATT GGCAACCTCT CTTGTGGAAC GTTTAATAAC AATTATTACA
AAATTACAAC GTATACATTA CATAGAATAG AGACTTCCGA CGCCAACATT TTTATGGTTG CATGTCTGAT ATGAATAAAG
GAGTGATTTA TAATGTGAAT GTTATTTGAA ATTAGAGTCA AGTAAATAAT AACCGTATGC ACAGATGTTG AAATACGTTA
TTTACCAACC GTGCCCGAAT GATTCTTCAC TTTCTTCCAC ATCTTCGTAA TTTAAAACTA GACCATTGAT CCATTATTGA
CCATCGCGGA ATAATTCATT TTCAGGTCGA TTTAATTTAA AACAAGGTTT AGACATAGTT ATAATTAGGT GCAGCCTTGG
AGCAGGCTTC CAGCGTGCAA TTTTGGTGTT TTTCTTTCAC TTATTAGGCA ATAATTTTCG CAGTGGTGGC TCAGTTCTAC
GACACCGACG TCAATAAGGA GTATGCGATT CGGGGGAATG CCGCCATCCT CAAGTGCCAA ATACCCTCGT TTGTGGCGGA
TTTTGTCAGT GTTGTTTCGT GGCATACTGA TCAGAACGAG AATTTTTATC CGGGCGCGCC TGACGGTAAT GACCGAAAGT
CGCACTTTCT TCCCTTGTGT CTCTCTTGCA GTTGTCCAGC AGTTTTACCA GACCGAAGTC AATAACGAGT ACGTCATCCG
TGGAAATGCC GCCGTTTTAA AATGCTCGAT ACCGTCGTTT GTCGCCGATT TTGTCACGGT CGTGTCCTGG CACGATACCG
AAGGCCACAG CTATGGGCTC GAGCAGACCA ATTATGGTAC TTTTGGGGTG GGGGGTCGAC AAGGGGCTGT GGAAGGACAT
GGCCCTTCAC ATTTGTTTCA AAACGATAGA ATGATTAAAC AAGAAACAAT AAAATCTTTT TTTTTAATTT TTTGCATATC
TCGGACACAG TTAACAATAC CTAAGCCTAT CTTATTCAAA GTGTTTCGCT TTATCCGTTT CTTTAGTAAA TAATACTTTT
AGGATGCTTT TTAGCCGAAA ATTTTCGATC AAAACACATC TCATTTTTGG TGGATGCTAA AGTATTGATT CCTCGTTTAA
AAATGACTAA AATCACCTAG AAATACTTTA ATAGTCGTCT TAATTTTGCA TTTTGTTTAA AATAAGACAA TTTTATTCGA
TCCTTGATTT AAAAATATAT ATTATAACGT TGTTTTTCTT CGGTAATTCA CTTATAGGCG TGACAAATCG GTCGGGAATA
ATTCAAGTCG GCTTATTTTG ATTTGATTTG ATTTGAAATT GATTCATAAC GTAGAAAACA TAATCATTTT GAAAATAATT
ACCTACCTTC TTGGCTCAAG AACGTGTTAA GAATCTAAGA AAAAAGTAAT GTTTTGAATA AATTTACTCA TTTCTCTTTC
GGGTTAAATA AAATTTTGTG AATTTTATTT CAGATGCGTG TTTCAATGCT TTGAAAAAAG CAAATCTTGC ACTATTTTTG
AAATTTCATA AAATATTCGA TTACCAAATT CGGCGTTTTT TTTCTTATTT TTTTTTCGAT GTTTCGCAAA AGAATTGCGT
TTTAAACGAG AACTTTACAA ATATCACGCC GAAATAATGT CATTGTAGAT TTAATTTTAA AGTGATTTGT AAAGCAATTG
ACGTAAAAAC GTGTTCAAGC ACCTGAAAAG GGCAAAAATA ACACCTCTTT GGACTGAGTT TATAACTTTT TTTTAATGTT
TGGTGAACAA TTGCTTCGAC CCTATTTATT ATGTATTGCT TTATTAACTC GACTTTTTAA AATTTTGAAA AAGCTTTTTT
TCTCAATGAT GCTCGAAAAT GCAAATATAT TGCCTCATTT GTAAACTAAG AGACTTCTCG GTTAATTTTT GTACTGATTT
TTCGGTTATC TTTTGAGAAA TTCTGCAAAA AATTGCGTCC TTGAAATACG AAGTAAGACG CCTGAAAAAT GCGAAACTAT
ATACCAATTT CACTTTTCGG GTTTATTTCA GATATCATGT TGTTAGTAAA ACATCGTGCT AAAACGGTCG GAAAAGGGAG
GAATATTGAG AAATTTTTGA GTTTTATGCC AGTTATTTTA GATGTCGGTT AAACTAAAAT AATTCTTGGT TACCAATTTT
TTTCTGATAG CGTATTTCAG CTTTGATTTC GATCTAACCG CTGTTAAAAC TTGACCTTTT TTGCGATTCT TGAGAATTTA
ACCCAATTTC TATTAAAGCA CTGAAATACG TTCTTAAAAT CTTGGGCACA GGATTTCTTG GGTTAGATAC CCTGGATTCT
AATCCCGTTT TTCTTCTCAG GTAACAAAAT TTCGCAAAAT CGTTTAAAAA AGAGAAAATA ATTTCGACA CAATTTGGAT
```

robert peuß 1/6/2015 15:31
Comment [1]: Exon 3
358 bp

TACGTTTGGC GAAATAATAG TGTTTCTAAT TGACATTTTT TGAATTTTTC AGCCAAAAAG AGCCATATTG CCTGGAATAA
GTTAAAATCG TTTTAACTTT TCACTGGTTT TGATGAAATA AGTTGATTTT TGGCCATTCT TTGCATAAAA AAATGAGCTA
AATCACTGGA AACACCTCAA TTTGTTTAAT AAGAATTGCT TGCTGTAAGT CCGAGAGGTG CATAGAAATG AAAAAAAAAG
AACTGGATCA AGCTTTAAGC AAATCCCTCA ATAAAAAAAG TTTTTGGATA GTTTTTGTCT TGGGGTTTGA AACCAACGTG
AAGGCTCCGA AAGTCCCAAA ATCCGGGTCT GGGCTTCCCA CAGCAGCGCG CCCGCAGTTT CCTCTTCCTT TCCACGGATG
GTTTATTCAC TTCCGGCCTT TCAGTCCGCC CTTTTTTCCA G**TGGTCAATC AGTACTACGA GGCCGAGGTG GTCTCCGAGT**
ACGTAATCCG CGGCAATACG GCCGTCCTCA AGTGCAACAT CCCCTCTTTC GTAGCCGATT TCGTCCGTGT GGAGGCCTGG
GTCGGCTCCG ACGGCTCTCT TTACAATCAC ACCGCCAATT ACGGTATTAA TCGATCGGCC GATATTTTTA TCGTACCTGT
TGCACCAATT TCCCGCATTA AATCGCTTCC TCTGCTCTCT TCCAGTGGTG AATCAGTTTT ACAAAGCGGA AATTTTGACC
GAGTATGTGA TTCGGGGGAA CACGGCGGTA CTCAAGTGTA GTATTCCGTC GTTCGTCGCC GATTTCGTTT ACGTAGAGTC
TTGGATTGAC GATAGCGGGA GCGTTATGAA AGCGTCGAGC GATTACGGTA ACTAATTGGG TTTTTGGTGT ACAGTTGTCG
CCCAGTACTA TGTGACCGAG GCCGAAAACG AGTACGTCAT TAGGGGCAAT GCGGCCGTTA TGAAATGCAA GATCCCGAGT
TTTGTGGCAG ATTTTGTGCA AATCGATGCC TGGATCGCAG ACGATGGCCA AGTTCATAAG TACAACGACA ACAGTAATTA
CGGTATTCAA AGTTAGCGAG TCGGAAGCAA ATCCTTTGAC CTTTTCGTTC CCATTGTTTT TATCCTCGCT CAGGATGTGT
CAGCTTCGCC GAGAATTGGA GAGAAGCTAT CTTTTTGAAC AGGGTCCAAG TTGTCTGGTT TTCTTGTCTT CATGTCCATC
TTCGATTACG TTATCTTCCT AAATTCTGGT TCTATAAATA TCTGAACTGG AGATGAGTTC ACGGAATATT TCAAATATTG
GCTTCCTGAA CCTACAGTTT CGTTATGGAT TTTACTCTGC AGACGGTTTG GATATGCTCT GTTTTCGTGT TTTTAATAGC
AACCAAAATA AATTACTAGT AACTTAACTT AACATTAAAG TAAATCGATT TTTTTAAATT ACTCAAATAT CGACGGGAAC
GTATTATGTT GTGAAGCAAC ATTTCATTTT GTTAAAAATC TTGAGTTATT GATGATCCTG AATACAATTT AGAAATTAAA
TTTTACTGTT ACGAAGAGGA AATTGCATTA GGGATATTGA TTTTCTCAAG TTACGAACGA GTGATGTAGA AACCGTTGAT
ATTATGTTTG TAATATGTCT GTTTTGCCAA ACAATAAAGG TTTATATTTA GTGCCTGTTT TACTTACCTG TGCTTTGATA
CTTTTGGAAG AGAAAAACGG AAAACGTGAA GGTTTAGTAA TGAAAAAGAT AATTAAAAGA CCAAAATGAA AAATACTTCT
ATCAAAGATA ATTTATGTAC CTGACCTGAT CGACTGTGAG TGATAAAAAC TTAAGACAAT GAAAAAAAAA GAGAACTTTT
ATGAAAATAC TTTAGACAGG TAGGTACCTC TCCTGACCTG ACCTGACTGT GGTGAGATGA AAACATTAAG ACAATAAAAA
AAATCTCAAA GAAAAGGAGA TTTTTTTAAA GATATGAAAA TACTAATAAG ACAATAAGGA AAAAGCAAAA ATCTAAAAAA
AAAGAAAACT TTCGTCAAAA ATATTTTAGA GCTACGTATA ATGTACCTGA CCTGACTTCA GAAAACATCA ATACAACAAA
TAAAAAAACA AATCTAGCAA AAAGAAGAAC TTTTTGATAT AGCAACATAA GAACAATGAA GAATAAAAGA AAACAGTATT
TCACTGACAA TATTTTAGAG AGATATTGTG TACTTGACCT GACCTGACCT GACTGAGTGG AATGAAAAAC ATTAAGACAA
CAAAGATAAA ACAAAAGGTC GAAAATAAGT GAACTCTTAT CAGTATTTTA GAGAGGTAAG TGTTATGAAC TGGGGCTGAC
TTGATTACAA AGATTAAAGA ACTCAAAAAA GAACGTTTAT TATGTACCTT ATCTCACCTA AGTTGTTATG TACCTGACCT
GAGCTAAGAC ATTAAAAAAA AATCTTAGAG AAGGAGAATT TTTTTAAAGA TATGAAAATA CTATTAAGAC GATAAGGTAG
GTATTATGTT CCTGAGCTGA CTTCAGTCAG AAAACATTAA AACAACAAAT AAAAAATAAA AATACTTTTA ATAACTTTTT
ATTATTGACA TAATAATACT ACGACAATGA AGAAAAAAGA AAGGAGTACT TCATTGAAAA TATTTTACCT GATCTGACCT
GACCTGACTG AGTGGAATGA AAAACATTAA GACAAGGAAA ATAAAAGAGA AGCTCGAAAA TAAGTTAATT TTTATCAAGA
GATAGGGGTT ATACACTGGA CCTGATCTGA TCTGACTGAG TGATATGAAA AAGGAAAAAC TCAACAAAGG AGAAAGTTTA
TTACGTACCT TACCTCACCT GAGTTGTAAA AAAAGATCTT TCATTAAAAA CAATCACTGA CTGATGAAGA AAAGAAAAAG
AAAAGACATC AAAAAAAGAA ATATCTCGAT AAAAAAGGAG AACTTTATTA GAGTTGGGAA ACTGTTAAGA CAGTAAAGAA
AAAGCAAACA AAAAATCTAG AAGAGAATGA GTACTTTTTT AGTGATATGC AACAATAAAG AAAAAAGCAT TATTATAAAA
TTAAAAAAAT ACATGAAAAT ATTGGGAGAT GAAAAAATGA AGACATCAAA AAAAGAAATA TCTCGAAAAA AAGAAAACTT
TTTTAGAGTT GGGAAACTGT TAAGACAGTA AAGAAAAAGC AAACAGAAAA TCTAGAAGAA GATGAGAACT TTTTTAGTGA
TATGCAACAA TAAAGAAAAA CGAAAACAAG ATAAAACTAA AAAAAGATAC CGTTCGAAAA AATATTTTAT TATTGCATTA
TGCAATCACC AATTGTGCAA TAATTTATTA AACAAAATCA AAATGTGTAA CGATGATAAA TCATGGCTTG ATGCATCAGC
AGCTTGGGAA TCTCTTATAC CTACAATATA TTTTGTTTCC TTTGGGATCA AAGTTTATTG AAACAATATA GACGTTGTAT
TATTATAATC GAATTGTGTT ATCTCATAGT CTGCAAAGAT ATTCGCTCGT TGTGAGTTTT ATAGTCCGGC TTTCATGCTT
TTATCTTAAC AAGACGGGTA TAACACTACA ATAATCTTTT ATACTGCGGC TTCTGTCCAC GAAAACTATC CATTTCTCGC
AACAATCTTC CACTGAAACG CAATATCTCA TCACTCCCAC CGATAACAAT CTTCCACGGC GTGAATCTAT TTGTGAAACG
TGTCCACCAC ATGTGTGAAC CCCGGGCGAC GTTTTCCCCT TGTTTTGATT GAAATTTTTT GGTGCAGTCG TCGCCCAATC
CTTCGAAGTG GAAGCCGACA ACGAGTACGT CATCCGCGGC AACTCGGCCG TGATGAAATG CGAAGTCCCT TCGTTCGTTT
CAGATTTCGT CCAAGTCGAA AACTGGCAGG ACTCCAACGG CAATATGTAC TTGCCGGGGG ACGACTACGG TAACGGCACC
GGCAACCCTC CCGTCCCGCA ATTTCGCCCA CTTCCAGTCT CTTTATTTTC CAGTCGTGAA GCAATTCTAT CAGAGTCGCG
TCATCGACGA GTTTGTCCTC AAAGGCAACA CTGGCATTCT CAAGTGTCTG GTCCCGAGTT TCGTCACAGA TTTCGTCCAA
GTGGAGGCCT GGCTGGCAGA CGATGGCACT GTGTTCGCTT ACGATCCCAC TCTGAAGGGT AAAAACCCGG GAAAAACCAA
TCGATCCTGT GTCTCTTCCC GAACTGAGTC CTGTGCTTGT TCCAGTGGTT AATCAATATT ACGAGGCGCA AGTTTACGAC
GTGTTCGTAA TCAAGGGCAA CACCGCGGTC TTCAAGTGCC AGATCCCGTC GTTTGTGTCC GATCATGTGG AGATCGTCTC
CTGGCAAGAC ACGGCCAACA ACAAATTTCT GCCTCCTGGT GACGATTACG GTAGTGCCAC TTTTTACGCG AAGCCACCAC
CTGTTGATCC CGTCCACTGC ACTCTCCATA CCAGAGCTGA TTCGATTTAA CAAAGGGGTG GATTTATTTG GGGTTTTCGG
CGGAGAATGT ATCAATATTC GTTCAAACAT AAGCGTATTT ATGGAAGCGG CGAAAATGAT AACCAGGCTA CTGACCCCAG
AAATCTTGCT CTGGCTTTCC AAACAATGTG GGTTTACAAA TGAAATGTAT GAAGCATAAA TGAGTTACAT AAAGTGCAGC
CGACCTGAAT GTGTGTTGAA ATAAATGGGG AGTTGGATAT TTTTGACGTT TTTGATCTTA TCGTGCGCAG CTGCACTGCA
TTTTACCTCT TGAATTTCGG TCGTCAGGAA GGTTGAAAAA CCAAAAATTA CACCAGTTTG TAGATAATAA CTTGTTTCAA
ATTTCAGCTT TAATCAAATT ATTTATGTTT ATTTATCTTC TATCAAATAA AAATTTTTCC TCCAACACGT CACAGTTTTT
TACTACGAAA TAGCTATTTG GTATAGCACC AAGGGAGGAA AGTCATGTAA TAATATGATT TTTTTCGACG GCTTCATCTG
AAAAAATCGT TTCCTACCTT CCAATATCCC ATGAAAGTAC GTTATTTCCT CCCGAAGGAA AAAAATCTAG AAACAGACAA
ACAGGGCGAG TATTTTGATC AAGATACAGA AACACCAGAA AACACAAAAT TACGTCCGAA ACGTGTCTTT CGAGCTTGAT
TTGTTCTATC TAGAGCATCT TCGGACGCCA AATTCGATAA AATTCGAAAG ATTTGTTCAA CAAAAATACA AAAGCAAATT
ACTTCCCAAA ATCTTAAAAT AACTTTAATC AAAACAAAGT TTAAAATCCT TAAATTATGT GTTAAATCTT TGTTATTTCT
ATGAATGTGA GAACATTTTT CAATAAAATA TTCCTTACTA TGTTGCAAAA ATTGTAGAAA GTTGCAGAAT TTAGTCTGAA
ATGAGTTATT AGCGCTTGAA TTGACGCTTG TATTTGGCTA AAAATTCATT AAATTCAAAA GATTCTCTTG AAACAATGTG
GAAAAGGTTC GTTAGATTTG CTTCATCTGA CATTTAGTAT AGCGTTTTTA TAAGAAGCTC ATTAAATTTA ACCGATTTTG
CCAAAATTTA GTACAAAATC ACTAAACTAT GAAATTTATG AGTTTTACCA AAAGTCATGG AAAATCGCAA AAGTGAAAGT
GAAAGTGAAT CATTTTTCAA AATTCCCAAA ATTACAAGAA AAATGCAAAA TTAATCTATT ATGTGTGATT GTTTTAATAG
TTTTAGACAT TTTTCGAAAA GAAATTCACT ATTAATTTCG AAAAAATCTG CAAAAAAACA AGCAAAAAAT TATTTATCTT
ATTTTTTCAA AGAAAAGTTT AAAAAATTTC GACAATTTGC CGAAACAAAA TGGAAAACCT TTAATAAATG TGAATTTTCA
ATAATTTCAG GAACCTTTTT AGTGACTTCC TAGCTTAGAA TAAGACATTT TGCCGAAAGA AAGCAAAAAA TCGAATGCAT
AATTTCTTGT AAATTTAGGA ACATTTTTAT GAAAGACAGA AAACTATAAA ACAAAGTCCG AACTTTGTTT TTGAGCCTGT
TTTAGATTTT AGAATTAGAA CAGTGTAAAA GCGTTACATC AAGTTTAAAA TATATTTTTC TGTGAAATTT TTTTTACTAA
ATGAGAAAAA TTCAGAAAAT TACAAAGTTA AGAAAATATT CACTTCTTCC GATAGGTTTC TTATAAGTGT TTTATAAGAA
ACATTAAATT CAACTGATTC GACTATAATT TAATAGAAAA TTTCCAAATT AGGCCAAATT ACAAAATCTA ATCCATTTTT

robert peuß 1/6/2015 15:31
Comment [2]: Exon 4
162 bp
Orthologous to exon 4 in D mel (Ig2)

GAGCTAAAAA CTAAGTAAAA TATTTCATTC CGATTTGTTG TTGTTTATGA ATTATATTTT TGTAATTTCA TTAGCTGCAT
ATTTCTTAAA CGTGACGGTT GATTTTTTAT TAGGTATATT TTTCAACTCA GTGCCTGAAA GTCAAGAAAT TATAACTTTC
AGCTAAGACA TAAAAATGTG TAAACTGGTG TTTTATTTTT GCTTCGACTT TCCTGACACC AATTAAGCGC TCCATATTAG
TAGCCTGTCA AAACCTCAAC CTTTATGCGG AACTTATGGC ACAGGAAAGC AGCGATGCAT TGAAAAAGAG ACATTTCCCT
CGATCCACTT AGACAGGTGT CCATCGTCCC GGGAATGAAT ATTTCATTGT TGATTTTCAT CAGTATTGCG TGCTTCATGA
AATAATCACA GCACTTCACT TCATTTCTTT TCCAGTTGTT TCGCAATCTT ACAACGCGAA TGTGATGGAC GAAAGCGTCC
TTAAAGGCAA CACGGCCATC TTCAAGTGCC ACATTCCCAG TTTTGTCAGC GACTACGTTT CGGTCGTGTC CTGGCTGCAG
GACGACAATA ATGAAATTGT CAACAAGGAC GATCGTTACG GTGCGTTGCG AGTCTTTTCG GTATTTTCGA TTTTTTCAAA
CGTCGTTGGG CTACTGGAAA TCCCATCCAA CGATCCTTGA TCCTGGCTGA CTCGACCCTC CCGCTCTGCC CGCTTTTGCC
CAATTTAACA CCGAGACCAG TTTTTAATTA ACACAATTGG GCAAATTCAT TCGATAAGCG CAGAATTAGT AATTAAAAAT
TGGTCTCGAA TAAGTTGCGG ATTCGAGGAA GCTTCAATCG ATACGGGTTG TCAACATATT AATTAATTTA TTAATTAGAG
CGTGCACTGT TGCCGGCATT TTCTTCAATC GGCTTTTATT GATTGACACC TTGAGTTCGC ATCAACTTTG AGAAATTTAA
CGTTTCAATT CTCCCAACTT CACTCTTGAA TTTGAGTCAT GTTCAAGGCT GAAAATTGAG AGCTTTGAAA CAGGCGGTTT
CAAATGACTA ATTTTTTTCT CAATTAACAG ACAGCAAATA TCTTGTTCTA CCATCTGGAG AATTACACAT CAGAGATGTT
GGGCCTGAAG ATGGTTATAA ATCTTATCAA TGTCGCACGA AGCATCGGCT CACAGGAGAG ACAAGATTAA GCGCAACCAA
GGGCAGACTG GTCATCACGG GTAAGTGATT TTGAATTGTG AAAAGTTTAA GTGGGGATGC CTTCAATTTA TAATTTTTGC

robert peuß 1/6/2015 15:31
Comment [3]: Exon 5
150 bp

AGTTGGTTTT TCGGTGGTAA TAACTTGAAA ACAAAGTTAA AGCTTCCGGA TAGCGAAACT AAGAAATATA AGAAATATTA
GCGAAATCAA TTTTTGTTTG CTCTTTTGGT ACAATTCTCA CCCGAATATT GACAACTGAT GAAAAGCGCT CAAATTGCTT
TTGTGACATT CCTAAAAAAT TCATGCCACC CACAGACCGA ATGTGTGAAT TCGAACTGTC GATTCCTTTC ATGAAGTAAC
GTTTATATTT AGCCGCTGAT AATAATTTGT TGTAAATAAA TTGGTGGCAT CCTTACAAAA TGATAAAAAG TCAACGCAGT
TAAATTTATT ATTCTTCAGC CATCATTCAT TCTAACGACC GTTCACATGT TGACTTTTTA TTATGCTTAT CGTGTTCGAT
GGCCACTTGA ATTCATACAC TACGCGTTTA CAATTTCTTG ATGCAACCGA GTTCCGTCTT CCTAACGTAA ATAAACGGCC
TTCCGAAATA TAAATATGGG CGACCGCACG ATAAATATTA ATATACGTTC CGATTATAAA AGCGAGGCAT GTTGTGTGCG
TCTCTTCAAA GCGAAAATTA TGATAAGACC CGCTGTTTGT CTTGGAAAAA TGTAATAAAT TCCTGACATA ACTGAGTTTC
CTGTTTGAAC AGCCGTGTAA ATATGTAGTA GCTTAATACG GAAAGTGTTT ATAGCGCTAA TTAAAACCGT TCCTATTGTT
TAATTATTTC AATTGTTTGT AATTCTTCTG CGGCAGGATG TCGCACGATA AGCCAAAATC GCGACAAGTG GCCATTGAAT
CGAGTTTCTT GACACAATCA CTCTCTGCTG TCTCAATCCC GCCTGAATTT TTAATGGATT AAGGCGCACC TTCAGCCATA
AATTACTGAA ATTAATTCAC TAAAAGCAGC AGACAGTGGA TTGGACGAAA ATTCTCCAAC TCTGGTCAAG CATTTCGGTG
CTTGAGCACA GTTCTTGTTA AATTTACCTG AATGTTGGGA TAGGGTACCC TTGCTTCTCA AACGGGCACT TTCAAAATTT
TGGAAATTAA GTACCTGTCC TTTTTTCCAA ACTCCCGCCA CCAAATCCCC GAACATTCTC GTGGCTCTCA CAGGTTTTTG
TAATTTTTGG GTACCCTGGC CCCTGATCGC CTTTTAGATT TTTTAAGGTA AGTTGGGTTT ATTCTAGAAC CTGTCGGAAG
CGTTCTTCCG AAATTTCCGC TGGAAACGAG GAAGGAGTAC GTTTTTTCGC TAACAGATAC TCTTAATTTG TTGTGCCCGG
CCCAAGGCTA TCCAGTGCCA CTTACAAGGT ATACATTTTT GGAAAAATTT GGTACTTCTA ATTGTAGAAC CGATCGGGAG
CGTCTTGCCC AAATTCCCCA CCACTGACAT TTCGAGAACC TTCAAGGGCC ACTTGCGGGA AAGTTTGACC CTTTTGTGCC
CAGCTCAAGC CTTCCCTGTC CCAAAATACA GGTAATTTTC ACTATCCTTT TCACAATCAT TTTTTTACTG CTAGAACCCA
TTGGGAGCGT TTTGCCCAAA TTCCCGGCTA CTGACAATTT GCGAGGTTTT GAGTCGAAAA GCAACTCCAC AGTTACGTTA
CTATGCCCTG CGCAAGCCTT CCCTGTCCCA CTGTACAGGT AACTAATTTT CTTCTCGTTC TTAGAACCCG TAGGGAGTGT
CAGACCAAAC ATCCCTAAAT TCCCAACTGT CGATAAATAC ACAGCGGGTA CCACTTTTGC GATTCTATGT CCGGGGCAAG
CCTTCCCGCC ACCACTTTAC AGGTAATGAA GCAACGGTAC CCATTTTGCA AAATTTTCGC AGAACCGGTC GGAAGTGTTC
CTCCGAAACT AACTTCCGGG GACCAATCTC GAACCGTTAG GGATAAATTC GCGTCAAATG TCACTCTCCA GTGCCCGTTT
CAAGCGTTCC CTGTCCCTAG ATTCAGGTAA TTCAGGGTAC TTTTTCTATA CTAGAACCTG TGGGAAGTGT GGCACCACGT
CTCACAGCCG GTACTTCCAA AATCCAGACT TTGGATGCTT TTCTCGGTGG CAGTTCGACG TTGTTGTGCC CAGCACAGTC
GTTCCCTGTC CCAAACTACA GGTACTTGCT TTGTTTTTTC TAGAACCTGT GGGTTCTGTC AAACCCAAAT TCCCAACAAT
GGACAAAAGT CATGCGTTTG AGAGTCTGGA AGGGAACAGT GTGACCCTGA TGTGCCCAGC GCAAGCTTAC CCTGTCCCCT
CTCTAAGGTA CTTATTTTTT GTCAATAGAA CCCATCAGTA GTACCTTGCC CAAAGTCTCG TCCCATTCAA AGTACGAGGT
CATTAATTAC AAGGAGAATT CGTCTCTGGC TCTCCTTTGC CCGGCACAAG GGTACCCAGT ACCGTTGTTT AGGTTAGTAT
TTTGTAGAAC CTGTGGGTAC AAGAGCCCCC AAATTCACCT CGAACGTCAA ACTGACTTCA TTTGAAGATC GGGAAAAGAC
CGATATTGCC ATGCTTTGCC AAGCCCAGGG TTACCCTTCA CCCACATTTA GGTAATTTAG AAAAAATCAG CCTTCTGGTC
TTTAGAACCG ATTGGTCTAC GAGCCCCCAC TTTAAATTCC GATGTTAGAA GTATCACAAT TGAGAAAATT TCCGGTCATG
GTTTGACGCT TTTATGCCAA GCACAGGGTT TCCCTACACC CACATTCAGG TATTTTCTTT TTTTCTAACT CTGATAATTA
TTTAGAGCCG GTCGGGACGC GAGCTCCTAA ATTATTAGCC GATTCTAAAT TCAAATACGT GGAGAGAGAA GCAGGTTTGG
AAATTCCGGT TTTTTGCCAA GCTCAAGGAT TTCCCGTCCC TTCATTCAGG TGATTTTTTA CTAATTCTCT CGTTTAG**AAC**
CTGTGGGGAC CAAAGCTCCG GCTTTTTCCA CCGATATTAA AGTCTTGGGT TACCAGAGAC ACTCAAAACA AAATGTTGCT
TTGTTGTGCC CGGCTCAAGG TTTCCCTTCT CCTTCATTCA GGTACTTGAG TTCAATTTTT TATTTGGTTT TCTTGTGTAG

robert peuß 1/6/2015 15:31
Comment [4]: Exon 6
124 bp
Orthologous to exon 6 in D mel (Ig3)

AACCTGTGGG GTATAAAGCG CCAAGTTTTT CGTCACTTTC CAAAACTTTT TCCTATGAAA TTCAACAAGG AAACGGTTTT
GTGCTGTTTT GCCCAGCTCA AGCTCTTCCA GTTCCAATGT TCAGGTACTG AAATTTCCAA AATTGTAACT TTTGCTCTCT
TGCTAGAACC AATCGGGTTT AAAGCACCAA CTTTTCAAAA CGATGCGACT AGTATTTCGT ACACTCGTAC CATTAATCAG
AGTTTTGGAC TTTTGTGTCA AGCCCAAGCC TATCCTGTAC CAATTTTCAG GTAAGTTTTA AGATTCTAAT TGGGTTTTTT
CCCGTATTTA GAACCTGTGG GCCTTAAAGC ACCGACCTTT TCCTCGGATT CTAAAACGTT TTCGTTTGAA CGGCGCCAAA
ATCACAGTTT TGCCCTACTT TGCCCCGCAC AAGCCCAACC TACCCCACTA TTCAGGTACG TCTAAAAATT TCTAATTTGG
TTTTAATTTA GAACCAATCG GGTCGAAGGC ACCGGCATTT TCCAACGATG CTGTTAGTTC GACTTACATT CGAACGGTTG
GTATGGGTTT CGGCCTACTT TGCCAAGCGC AAGGATACCC AGCCCCCTTA TTCAGGTACT TATTACAATT TTTTGAGTAG
AGCCCCAAAC CAGTGTTGCC CCCACCGCTA AAGACAAGAC AATCTCGGGT CTTAAGAACT TTGCACCGGC GGGCAAAACG
GTACCACTTT TGTGTGCGGC TCAAGGGTTC CCTGCACCTA TCTACAGGTA ATTGCAAGGG TACTTGCTAC TCCTCTCATT
TTTTCCAGAA CCCACTGGTA AAATTTCCCC AAAAATCCCC GGCCAAAAGT ACGAAGGCGG CAAAATTTTG ATCAGGCCCC
AAAACCGAAC CACTTTTCTT ACCTGTGACG TGGTGGGGTA CCCCGTTCCT CTATACAGGT AATTGTTTTT CCAGAACCGA
CCAATAATAT CGCGCCCAAA GCCCTCGATA AGAAAATTGG GTTTACGGCC ATTTCGAATA GTGTGAAAGA GAGTGTTTTT
CTGATGTGTC CCGTGGTGGC GTTCCCGGTA CCACTCTTTA GGTACTTGTT TGTTTGATTG CATGGGTACC CGTTTTTTTT
TTGGCCACGT TTCTGATCCT TGTCAACTTC TTCCAGAACC CACGGGAAAA ATTTCTCCTA AAGTCCCCGG TCGAAAGTAT
GACGGGGGTG TGATTTTGAT TCACCCTCAA AACCATACAG CTTTTGTTAC TTGTGACGTG GTTGGCTACC CTGTCCCGTT
ATACAGGTAA TTGATTGGGA AATTTTTACA GAGCCGACCA ATAATATCGG CCCAAAGGCA TTAGATAAGA AATTTGGCTT
CACTTTGGTG TCAGAAAACG TAAAAGAGTC GATTTTTCTC ATGTGTCCCG TCGTGGCGTT CCCTGTCCCT TTATTCAGGT
ACTTACAAGT ACTTACTCTT TTGCATGGTT ACAGAACCCA CAAATAATGT CTCTCCGAAA AAAGTGGGAG CAGAGTTTGG
AGGTTGGAGG GTTTTAAATG TTGCGAACGG TACTATTGCA TGGTTAACGT GTCAAGTGAG CGGGTTTCCA GTACCACGTT
TTAAGTAAGT CCAAAATTTT GTTGAAAGTG CTCGTTTTGG TCTTTGGACT TTCCATCATC TTTGGGTTTT CTTCCAGAAC
CCACAAATAA CGTACCTCCC AAAAAAGATT TGCAACGGTT TGAGGGTCAC CAGATTCTTA ATGTACCAAA CCTGGGTATT
GCCTGGTTAA CTTGCCAGGT TACCGGTTAC CCTGTTCCTA GATATATGTA AGTAAAAAAG TTTGAAAGGC CCGTTTGAGT
TACTGTTTCG GTTGTTATGT CTCGGTTCAA TTCCTTTCTA GAACCAACCA GTAACGTAGC ACCAAAAAAA GATGGTGAAA

AGTTCGAAGG CTGGAAAGTA CTTCTAGTGG TACAAAATAG CACGGCTTGG TTATCTTGTC AAGTGACCGG ATACCCGGTC
CCGCGATACA TGTAAGTACC CGTAGGCTTA AGACAAGACA CTGCTTGGTC AACTAAGACA GGTAAGTGCA TGCATGAACC
AGCATAGGGA TTCTGCATTG TTTCGTTTTG CTTTTTCTCC TCTCACAGTT AGCTTGTTAT CACGCCTGTT GAGTACCTCC
AGAATCCTAA TTGCTGGTTC TTTTCAGAAC CTACTAATAA TTTACCACCA AAACTCGTGG GTGAAAAAAC TCCATGGAAG
ATAACATCTT TCCCCGAACA AAGCGATGGC GTAATTATGT GTCCAGTCGT TGGTTTTCCC TATCCGGTAT TTAAGTAAGT
TTGATGTTGC TAGTGTAGTA CTGTCACCTT TTCCTTGTCA CATTCTTACA TAAAATTTGT CATAGAACCG ACCAACAACG
TCCCCCCGAA ATCGTCCGGT CGCAAAATCG ACGGTAGCAT TGTCATTGAA GTACCTCTCA GTCAAGATAT CGTAATAACT
TGCGAAGTTT CCGGTTACCC TATCCCTAAA TTCAGGTACT TACAGCCTTA CAATTCCGTT TTGTCTAGAA CCGACTGGTC
TGACAGCCCC TAAAATTTCC GGAGACGGTT TAAATAAAAA ACTCGCTCAT AATTTCCAAC ACGTTGCTCT TCTATGCGAC
GCTCAGTCGT TCCCAGCACC TGCCAAAAGG TACTTCTTCT CTTACTTGTG ATTTTTCAGA ACCGACTGGA TTCGTTCGGC
CCCAATTAAT CTCCCGAACG ATTGATATAA GGGTATCAAA ATTAAACGAA ACAGCTGTGG CCTTTTGCCC CGCTCAATCT
TTCCCCGTTC CATTATTTAG GTAAAAAGCG ACCCGCATTT TTGCATTTTT CGGACTGTTG CTCGACACGC TTCTCCCTTG
TTCCAGGTGG TACAAATTCG TCGAAGGGAC CACTCGAAAA CAAGCGGTGA CTCTCAACGA CCGAGTCAAA CAAGTCGCCG
GAACTCTCAT CATTCGCGAA GCCAAAGTCG AAGACTCGGG GAAATACCTC TGCGTTGTGA ACAACTCCGT TGGAGGAGAG
AGCGTTGAAA CGGTCCTCAC CGTCACAGCG CCTTTAAAAG CCAAGATCGA ACCGCAAGTC CAGACTATCG ATTTCGGAAG
ACCCGCCACT TTCACTTGCA ATTTCGAAGG TTGGTTGCTT GTCCACAGTT GGATTTCCAT TTATTTCCAT TTTTTTTTGG
CAGGTAATCC GATTAAAACT ATAAGTTGGT TGAAAGACGG CCATCCGATC GATCACAATG AAGCCGTTTT GCGGATTGAA
TCGGTCCGCA AGGAGGACAA AGGCATGTAC CAGTGCTTCA TCCGCAACGA CCAGGAAAGT GCCGAGGCGA CCGCCGAGCT
GAAACTCGGA GGACGGTTCG AACCTCCACA AATCAGGCAC GCGTTCAATG AGGAAACAGT CCAGCCGGGC AATTCAGTTT
TTTTGAAATG TATCGCTTCT GGAAACCCGA CACCCGAAAT CACGTGGGAA TTGTACGGAC GGAGGTTGTC CAACAGTGAG
AGAAACCAAA TCGGACAGTA CGTGACAGTG AACGGTGATG TTGTCTCGCA TTTGAACATC ACAGCAATTC ACACGAATGA
TGGTGGCTTG TATCGGTGCG TCGCTAGTAG CAAGGTGGGC TCAGCGGATC ACTCAGCGAG GATAAATGTT TACGGGTTGC
CTTTTGTGAG GTCCATGGAG AAGCAAGCCA TCGTCGCGGG AGGTACACTC ATCGTCCACT GCCCTTTTGC GGGACATCCA
GTCGATAGTG TCGTATGGGA ACGAGGTTAG TTATTTTTAA TTTGTTCTTT TATTTTGTCT CTTTATGCCG AGCCAATTAC
TTTTGAAAGT ATGCAGTGAA ATAAATTAAT TTATTATTGA TTCCAGATGG GAGACAACTG CCGATCAACA GGAAACAGAA
AGTCTTCATC AACGGCACCC TCATCATCGA GAACGTCGAA CGAGCGTCCG ACCAAGCGAC CTACGACTGC GTCGCCAAAA
ACTCTCAGGG ATACAGTGCC AGAGGCTCTC TCGAGGTCCA AGTCATGGGT GAGTACAACT TTTAGTCTTT GTTGTACCCA
CTGCCACATC ACGTGACCAC ATTGGGTGAT GTGGCGGTGC AAATTTACAG CTTTGTGATA GGATTCGCGT CGGCTGGACC
ACCATACCCT GTATGTGCTT ATAATTAAGT TGTGATTTTC TTTCAGAGCG TGTTGCCGCG TAAGGTAAGC CGTGAGTGTA
TCATGCATAT CACCGTAAAC TCATTATTTC GTATTGTTTG TATACATTTC ACAGAAGAGG CCCATGTGTT ATTTTGTCCC
GTTGAATTTT TTTACAGTGG CACCTATTAT AAGTCCTTTT CAGTTCGATG GTCCGTTGAA TGCCGGCGAT ACGGCCGTTT
TGACATGTTA CGTGCCGAAA GGCGATCGTC CCCTTAAGAT CAAGTGGTTC TTTAACGGCA GACACATTAC ACATCATACG
AGAGGAATCT CGATCTCTTC ATTCGGCAGT CAAGCCAGTA TTCTTAATAT TAATTCGGTG GAGTCGCACC ATAAGGGCGA
ATATGCGTGT GTCGTCACGA ACGCGGCGGG GAAGTCCAAA TATGTTGCTT TTTTAGACGT TAATGGTACT AGACTATTTG
TTGTTTAGTT CATTTTTTGT TTCTACCTTA CCTCAAACCA TGCGCTGTTT CACCTCAAAC TTTCGAAATC TTCTAGTGGC
TCCTCAAATC TTGCCATTTG ACTTTGGGGA AGACTCAGTC AATGAGGGAG ACACAGTTTC GTTGCAATGT ACCGTAGTTA
AAGGAGACAA TCCGTTAAAC ATCACTTGGA TTTTAAACAA TAAAACTGTT GACGAATCAC ATGGAATTAT CATTAGTCAA
TTAAAACGAG TTAGTCTAAT GACAATTGAC TCGGCCCAAG CGAATCACGC TGGACGATAC ACCTGTATCG CCTCAAATCC
AGCCGGAAGT ACCACTTTTT CGGCAGATTT GAACATCAAC GGTGCGTCCA CTGTCTAACA CTTTTTCTTG TCCAG**TACCT**
CCCCAAATCC TTCCTTTCGA TTTTGGTGAA GATTCGATCA ATGAAGGAGA TGGTGTTTCT GTCCAGTGCA CTGTATCTAA
AGGGGACTAT CCTCTAAACA TTTCATGGAC TTTGAATGGC CATTTCATCG ACCAAACTAA CGGCATTGTC ATCAATAGGG
CTTCAAAACG AGTCAGTACT TTAAGTATTG ACAACGTCCA AAGCACCCAC GTGGGGAATT ACACGTGCCT GGCCAGCAAT
TCAGCTTCAG TGACCACTTA CACGACCTCA TTATTTATCA ATGGTACTTT TATGTTTCTC TTTTAACACT TTTCCTTCAC
ACACCTAGTA CCACCCCAAA TTGTCCCTTT CGAATTCGGC GAAGACCCGA CAAATTCAGG CGACTTAGCA ACTGTGAATT
GCGCCATAAC TAAAGGAGAC CTCCCCATTA ACATAACGTG GAGTTTGAAC GGTCAACCAG TTCAAACCAT TGGAGGAATA
ACCGTTGGTC AAATGGGACA ACGGATAAGT ACCTTAAGTA TCGACTCAGT TGATGCAAAA CACGCCGGAA CCTACATGTG
TACTGCCCAC AACAAAGCGG GGACGATCAC TCACTCATCC ACACTCTATG TGAACGGTAC TACTCTAAAA ATCACTTTTT
GTGCCCCAGT TTCCCCCCAA ATCCACCCTT TTGAGTTTGG CGACGAGTCT GTCAACTCAG GCGAGATGGC AATCGTGCAC
TGTGCCGTAA TTAAGGGCGA TTTGCCTCTA AAAATCACGT GGACACTTAA TGGTCGCCCT ATTGAAACGG TAGAAGGTGT
GAGTGTCATG CAGACGAAAA AAAGGGTCAG TCAGTTGACC ATTGACGACG TCCAGGCCCA CCACGCTGGG ACATACACTT
GTTGGGCGGC CAACAACGCG GGAAATGCCA GTTTTTCGTC ATATTTGCGC GTCAATGGTG ATTGCTGTTA GCTTAGGTTA
TTTATTTTAG TACCACCCCA AATTGTCCCT TTTGATTTTG GCGAGGAGTC TAACTAAAGG CGATTTTCCG ATCACTATCA
CTTGGAATTT GAACAATTGT TCGATTAGTG ACATCTCAGC GGTCACTGTT TCCAAAATCA ATAAAAGAAT CAGTACTTTG
AGCATAGATT CGGTCGAGGC GGTGCATTCA GGCACATACA CCTGTCTCGC CGTAAACAAG GCAGGGACTT CGAGTTTTTC
GGCGGTTTTG AACGTGAATG GTACTTTCGA GTGTTAGAAG TGTTGTTGTG ACTTTATTTT TGTTGGAAAG TTTTTGTTTT
GGTTTGTAGT CCCTCCCCAA ATTCATCCGT TCAATTTCGG AGACGAATCG ATTAATTCTG GTGATGCCGT GATTGCCACT
TGTGCAGTAA CCAAAGGTGA TTTTCCACTT AAGATCCGTT GGACTTTGAA TAATCAGCCA CTTAGCGACT TTGATGGTAT
AACAATCATG AATAATAAAA GAGCAAGCCA ACTTACGATC GAATCGGTCC AAGAGCGTCA CACAGGGGCG TTTAAGTGTA
TTGCGGAGAA TCGGGCGGGA GTTGCAGAAT ATTCGACTCA TCTCAATGTT AATGGTATTT TTGTCGTTCG ATAACACAGT
TGTAGCCTTC TGTTCCGTTA AACTTTGATT TCTACCTCTA GTCCCTCCTC AAATAAATCC CTTCGAGTTT GGCGAAGATG
CGGTCAATTC GGGCGATCTT CTCATGACGA CTTGTTTTGT AACAAAGGGT GACTTACCAA TTCAGATTTC ATGGACTCTG
AATAATAGAC CCATCAACGA ATTCGATGGT GTTAGCGTTA TGAATACTAA TAAAAGAGCC AGTCAGCTCA CAATAGATAG
CGTGGAAGCA CACCATCGGG GGGAATACAA ATGTATCGCA GAAAATAAGG CCGGAGTGTC CGATTTTACG ACATTCTTGA
ACGTTAATGG TACTTGCACG ATGTATTGTT TTATTTTAAT TATTTTGACC CTGTCTTTGC ACTATTCCAC GTGGCCCTTC
TGTCGTTCCA GTTGTTAACA TTTTCAGTTC CTCCTCAAAT CAATCCCTTC GAGTTTGGTC AAGATTCGGT CAATTCTGGC
GACCTTCTCA TGACTATCTG CTTCGTAACA AAGGGTGATT TGCCGATTAA AATCTCTTGG ACTTTGAACA GTAAACCTAT
CAGTGAAATT GATGGTATTA CTGTCATGAA TACCAACAAA AGAGCTAGTC AATTGACGAT TGAATCGGTG GAGGCTCACC
ACAGAGGAGA GTACAAATGT ATTGCAGAAA ACAAGGCAGG AGTGTCAGAA TTTTCAACAT TTTTAAACGT TAATGGTATT
TTTCCTTTTT ACTCTGAAGA TTTTTCTTTT TACACCTATC CCACCTTTTA ATCTTATCCA AAATAAAAAC AAATAGCGTG
GGTCGAACTT TCTACACTGT TTTTGATTTT CTGTTTTTTG TTTTTGGCAT TGAACAGTTG TCATTGTGTT TCGCAGTTCC
TCCACAAATA CATCCTTTTG ATTTTGGCGA AGACGCTATT AACTCCGGAG ACATAATAAT GGCAACTTGT TTGGTAACCA
AAGGAGATCT GCCGATTAGG TTTCACTGGA CATTAAATAA TAGGGAAATC GCAGATTTGG ACGGTATTTC TGCCACTAAC
GCCAACAAAC GAGCTAGCCA ACTCACAATA GAGTCTGTAC AAGCTCATCA CAGGGGAGAA TATAAGTGTG TGGCAGAAAA
TAAGGCTGGC GCGTCCGAAT TTTCGGCATT TTTGAATGTT AACGGTACTT TTAACTTTAT TTTTCCAAAA GTTAGTCTAC
CTTCCAACCC TTAGAACTCG CATTTTCTTG ATTAGAAAGT CTTCACTCGT TTTTCTATTA CCACATGGGC CTTTATTTTT
GGTTATACTA TTCCTGTTTT CAAGCACATT TATTGTACCT TTTAGTACCT CCCCAGATAC ACCCTTTCGA TTTTGGCGAA
GACCCTATTA ATTCGGGCGA CATGGTTATG GTAAATTGTT TCGTAACAAA AGGTGATCTA CCTATCAAAA TCTACTGGAC
ACTTAATAAC AAACGCGTAA ATGAATTGGA CGGTATTTCT GCTGTCAAAG CCAATAAAAG AGCAAGTCAG CTTACTATAG

robert peuß 1/6/2015 15:31
Comment [5]: Exon 7
263 bp

robert peuß 1/6/2015 15:31
Comment [6]: Exon 8
582 bp

robert peuß 1/6/2015 15:31
Comment [7]: Exon 9
162 bp

robert peuß 1/6/2015 15:31
Comment [8]: Exon 10
288 bp
Orthologous to exon 9 in D mel (Ig7)

```
AGTCAGTGCA AGACCACCAC AGAGGGGAAT ATAAATGTCT GGCTGAAAAC ACTGCTGGAA TTTCCGAATT TGCAGCAATT
TTAAACGTTA ATGGTACTTT AGGTTCTATT TTAACTGTTT GCACTTTTTC CTTATCCAGT GGCTCCCCAA ATCATGCCTT
TTGACTTTGG CCAACAACCT GTAAACTCCG GTGATATGAC GACAGCACAG TGTGCTGTTA CCAAAGGCGA TTTTCCCATA
ACCATCACAT GGGCTCTTAA TAACCAAACC ATTAGTACCA TTTCTGGAAT TACGGTCTCA CAAACCAATA GGCGCATCAG
CAGTTTGAGC ATAGAATCGG TCGAAGCGAT CCACTCTGGT ATTTTCACGT GTACTGCAGT AAATAAAGCA GGGTCTGCAA
GCTACTCATC GGTTTTGAAC GTCAATGGTA CCACTCTAGT TTGCTGCACA TTTTTCTTTT AATTTTCTGA CCAACCATGG
GCCTCAAACC TTCTGTGGAC CTACCTCCCA TGTCAAATCA TCCCTTTACA GTGGCACCCC AGATTTTGCC CTTCGATTTT
GGCGAAGAAT CCGTCAATTC TGAAGATGTA GCTTCGGTTC AATGTACCGT TTTTAAGGGC GATCTACCCA TCAAAATTAC
ATGGTTGCAC AACAATAAGA CTATTGAACC CAATCATGGG ATTGCAATTT CAAAAGTTAG CAAAAAAGTT ACTTCTTTGA
CCATCGACTC AGTACAAGAG GAACACGCAG GGTCGTATAC TTGTCTAGCT CAGAATAAGG CCGGAAGTAC CAGCTACACG
GCAATTTTGA ACGTCAACGG TACTTAATTG TGTTGACTCG TGCTAATTTA TAATTTCCTC CTTACGACTT CCAATTTTTG
TGAATTTCCA GTCCCTCCTC AAATTTTACC TTTCGATTTT GGCGAAGAAT CGGTAAATTC TGGCGACGTT GCATCTTTGC
AATGTACTGT GCATAAAGGC GATCTTCCGA TAAACATCAC GTGGCTCCAT AATAACAATA CCATTGGTTA CAATGATGGG
ATTTTAATAA CCAAAGCTGG GAAAAAAGTT AGTACTGTGA CCATAGATTC GGTCCAAGCA CAACACGTCG GAACCTACAC
TTGTTTTGCC GAAAACAGGG CCGGAAGTGA TAATTTTTCG GCTTACTTGC GCGTCAACGG TAAAAATAAT CGTAGTTTAG
TTGTCACTGT CCATTCCCCG CCTTACATAC CCTTTCGATG GTTATAGTTG CCCCTTCAAT CCTCCATTTT GATTTTGGGG
GAGAACCGGT TAATTCTGGC GATTTGGCTT CTTTGACTTG TTCGGTCTTC AAGGGTGACC TCCCAGTGAA CATCACGTGG
TACCATGATA AGAAAATGGT GCGTGATAGT GACGGTATTC TTGTTAGCAC AGCTGGACGT AAAATTAGTA CTCTCAGCAT
TGACTCAGTA CAGGCACATC ATGCAGGAAC TTATACTTGT CTTGCCCAAA ATCGGGCTGG TACCACTTCG TACTCGGCAG
ATTTGCTCGT TAATGGTACT TTTTTTAATA ATTTTATAGT TTTCTAGTAA TTTTATTTTG TTAAATCCTG AACCAACCTT
CCAGTGGCCC CCCAAGTCCT ACCAATTAAT TTCGGCGAAG AAACTATGAA TACAGGGGAT TCGACTTCTT TGACTTGTAC
TATAAGTAAA GGAGATCTTC CTGTTGAAAT AACATGGTCC CACAACAACA AAACTATTAC CAATAGTGAA AATATTGCTA
TAGTTAAAGT GAGCAAAAAG ATTAGTACAC TAAGCTTTGA CTCAGTCCAA GCTGAGCATA TCGGGGAGTA CCGATGTACC
GCCAAAAACT CAGCAGGGTC CACAAGCGCC TCAGCTTTCC TAAATGTTAA TGGTACTTTT ATTTTTGGTT TCTTTCACAT
TTGCTTGCAC CTATCCAAAG TACCATCCTA TTTCAGTTGC TCCCCAGATC CTCCATTTCG ATTTTGGTGA CGATCCCATC
AACACTGGTG ATTCAACTTC TCTGACTTGT TCGATAAATA AAGGAGATCT ACCGATTGAT ATCACGTGGT CTCATAATAA
CAACACTGTG GACATGTACG GTACCATCTC AGTGATTCGA GTCAACAAGA AAATCAGTAC ATTGAGCATA GAATCGGTCC
AAGCAGAGCA CATTGGAGAA TACACCTGTA TTGCCAAAAA CCGGGCTGGA GTCACAAGCT ACTCGACGTT TCTCCACGTC
AATGGTACCT AAGTGTGTTT TTTGTTCTAG TACCACCCCA AATCCTTCCT TTTGATTTCG GCGACGATCC TGTCAATTCT
GGCGATGTCG TTTCTTTAAC TTGTTCGGTA AATAAGGGTG ACTTGCCTTT GCGCATCTTT TGGAGTTTCA ACAATGATAC
CAGTGTCGAC AAATACGGTA TTGTGGTCAG TTTGGTTAAC AAACGATTGA GTACTTTGAG TATCGATTCC GTGGAGGCCC
ACCATACAGG AGACTACAAA TGTATCGCCA AAAATTCGGC CGGCGTTAGC ATACATTCGA CCCAATTGAA CGTTAATGGT
AATAATCAAA GCAAAGCTTT CTCTCCCTAG TACCACCCCA AGTTGTCCCC TTCGAGTTTG GTGACCAACA AGTCTATTCC
GGCGATAGCG TTTCCGTCGC TTGTTCCATA AGTAAAGGGG ATCTTCCCTT GAATATCACG TGGTTATTCA ACGGAGTTAT
TATCAACTCG CCGGGCGTTT TGATCAACCA AGTAACCAAA AAACTAAGTA CTTTAAGCAT CGACAGTGCC ACGGCGGACC
ACACAGGGGA ATATACATGT CGGGCGGCGA ATCGAGCCGG TTTTGCGACT TTTTCGGCCT ATTTATACAT CCACGGTACT
TAGTAGACTT AGTTTTCTTA CTTTTTGCAC CCAGTACTAC CCCACATCAC TCCTTTCGAA TTCGAGGGCG AATCAAACAC
TGGCGATAAC GTCCAAATCA ACTGCCACGT GAGCAAAGGT GACGTTCCTT TGAACATCAC TTGGAGTTTC AACGGGGCAC
CCATTAACCC ATCATCTGGG ATCACAACCC TTCCAATCGG GGCTCGTACC AACCTACTAA ATATTAATTC CGTTGATCGG
GAACACGTCG GAAATTATTC TTGCAAAGCG ACTAATCGTG GCGGTACCGC CTCACATACT GCCACCCTTT TTATTAATGG
TACTTTTTTG TAATATTTTG CACTTTTAAC CCACAGTACT ACCCTATATC CTCCCGTTCG ATTTTGATGG CGAAGCCAAT
ACTGGGGATA GTACCCAATT GACTTGTTAC GTGAGTAAGG GGGATACTCC CGTCAATATC ACGTGGACTT TGAATGGAAG
AGAAATTGAT TTGGATTTAG GGATTACGAC GACTTTAATT GGCAGTCGAA CCAACTTATT GACGATTAAT GCCGTACAAC
CTTCACATAG TGGGATTTAT ACTTGTACTG CTTGTAACAA GGGCGGGTCA GCAGTCCACA GTGCTGAACT CTTTATCAAC
GGTACTTCCG TTCTCCCTTT TTAGTTTTTC GCTATTAGTT AGTTTTATTA GTTTGAATTC TTTCACAGTA CCACCCCAAA
TAGTCCACTT TGATTTCGGC GACGAACCTG TTAATTCTGG CGACATGGCT TCTGTCACGT GCATTGTTAC TAAAGGCGAT
TTTCCAATTG AAATTACTTG GAATTTAAAT AACCAATCCA TCGAGACAAT TCAAGGAATC ACAGTCATGC GTACCAACAA
ACGAATCAGC CAACTCAGCA TCGATTCAGT CCAAGCCGAC CATTCCGGCG AGTTCACTTG TATGGCCAAA AATCCGGCCG
GCACAGCATT CTACTCCTCA ACATTGCATG TCAATGGTAC CAAAACCCAT TTTTGTTGTA GTACCTCCCC AAATTACTCC
TTTCGAATTT ACCGACAATC CTGTCAACTC AGAGGATATG TGTTCCTTGT TGTGTACCGT CAGTAAAGGG GATTTTCCCA
TTGAAATCAC TTGGAGGCTT AACAACCGCT CAATTGAGGC GTACCAAGGA ATCTCAGTCT TGCGTACTAA TAAAAGGATC
AGCCAACTCA GTATTGACTC AGTCCAGGCC GAGCACGCCG GCGAATACAC GTGTCTTGCC AAAAATCCGG CTGGAATAAC
CCACCAATCG GCCATTTTGC ACGTTAATGG TACATTTAGG TTTTTTATAT TTTAGTACCA CCCCAAATTA TCCCATTTGA
TTTTACCGAC GATCCGGTCA ATTCCGAAGA CATGTCTTCA CTCACTTGCA CGGTCAACAA AGGGGATTTT CCGATTGCGA
TATCTTGGAG TTTGAATAAT CGTTCAGTTG AAACAATTCA GGGAATCACA GTTATGAGGA CCAACAAAAG GATCAGTCAA
CTCAGTATTG ACTCAGTCCA GGCTGAGCAC GCCGGCGAAT ACACTTGTAT TGCCAAAAAT TCGGCCGGAA TCACACACCA
ATCGGCCGTT TTGCACGTGA ATGGTATTTC TGATTCGTTT ATTATTTATT CCCAGTTTTG CCCCACATTA CCCCTTTTAA
TTTCGACGGA GAGGCCAATA GAGGGGATAG TGTGCAAGTG TCGTGTTACG CTAGTAAAGG GGATATTCCG GTCAGTTTTT
CCTGGCTTTT GAACGGAAAA CCTACTTCTA ATTTGGAGGG AATTGGAGTT TCTTCTTTTG GGAAAAAAAC TTCAGTTTTG
AGTATTGACT CAGTAGATGA GCACCATGCC GGGAACTACA CGTGTTTGGC CTCAAATCGG GCAGGAGTTT CGGCATTTAC
GGCAACTTTA ATCGTCAAGG GTACTTGGGC ACGCTTTTTT GCTTTTTGAA CCAAAGTCCT TGACCCAGAT CCAAATTGTT
TTTTGTTCCT AGTTTTGCCA AAAATCCAAC CTTTCAATTT CGGAGATGAG CCAAAATTTA TCAAAGATTC GGCCAACGTG
CAATGTGGTC TTTCATCGGG TGATACCCCC GTGATCTTCT CGTGGATGTT AAATGGCAAA CCAGTTGAAA ACATTGATGG
AATAACTGTT GGGAACTTTG GCAAAAAAAT GTCGGTTTTG AGTATTGACT CACTTTCGGA AATACACGCC GGAAATGTGA
CATGTTTAGC TTCCAACAAG GCCGGAATTT CAAGCTACAC CACCGAACTC ATAATCAAGG GTACTTTCAC ACTTTTTCTC
ACGTTTCCTT TCTTTTTTCC TAGTGTTACC ACGAATTACC CCTTTTTATT TCGAGGACAA TCCCGTCCAT TCCGGTCAGT
ACGTTCAAGT CAATTGTCTC GTATCGGACG GCGACTTACC AATAGAAATC GAATGGATGC TAAATGGGAA GAACATTGAA
GATTTCCTTG AAATTTCCGC TTCAAAGATA GGAAAGCGGA GTTCAATTTT GACTATAGAA GCCGTTTCAT ATATCCATGC
CGGGAATTTC ACTTGTCTGG CCAAAAACAG GGCCGGAAGT GCCAAATTCG TCACCCAACT ACAAGTTAAT GGTTGATTGT
GTTACAAATT TTCCTCCCTT CTTTTGCTTT ATTGTTTTTG CATTGTTCCT TCCAGTTCCT CCCCAAATCA CTCATTTCGA
TTTTGGCAGC GAACCCATCA ATTCGGGTGA CATGGTTTCC GTGTTTTGTA TCGTGAACAA GGGGGATTTC CCTTTGGAAA
TACAGTGGAC CCTGAATGGG AAAAACGTTG GGCAAATTGA TGGTATTACG GTACTGAGGA CCAGCAAAAG AATCACTCAG
TTGAACATTG ATTCTGCCCA AGCCGAACAC TCGGGGAAAT ACGTTTGTTT GGCGGAAAAT CCGGCTGGGA AAATCGAGCA
TTCGGCCTAT TTGCGAGTAA ATGGTACTTT TGATTTCCCT TCTTTTTAGT TTTACCAAAA CTTCTCCCTT TCACATTTGG
TGACGAACCC TCCTATCTGG GTGAATCCAC AACTGTTCAA TGTAGTCTTT CTTCGGGTGA TATGCCGGTG AAATTTTCTT
GGACCTTGAA TGGCAAACCT CTAACCGATG TTCAGGGCGT GAATATTGCT TCTTTCGGCA AAAAAACTTC CGTTATAAGT
ATAGAATCGG TAGATGAGCA CCATGCAGGG AATTACTCAT GTCTCGCAGA AAACAAAGCA GGAATTGCTA GTCATTCCTC
TGTCTTAACA GTGAAGGGTA ATTGTCAATT CTGTTTTGTA TCCATTTAGT TTCACCCCGA ATCATCCCTT TCTATTTCGA
```

AGAAAATCCC CTTCATTCCG GTCAGTATGC CCAAATCAGC TGTTTAGTAG CTGAAGGAGA CCTCCCTATC GCAATAGATT
GGACTTTAAA CGGGAAAAAC CTCGATAATT TTGCCGAAAT TTCGACCGCA AAAGTAGGAA AAAGAAGCTC AATTTTGACC
ATTGAATCTG TCTCCTACAC CAGCGGCGGG AATTACTCCT GCCGGGCTAA AAACACAGCA GGAGAGGCCG AATACGTGAC
TCAGCTTTTA GTCAACGGTT AGTTTTGAGT AGTTTTTCTT TCCAGTTTTG CCCCACATTT CACCGTTCAG TTTTGGCGAT
ACTCCTGTCC ATTCCGGTCA AACCGCTCAG GTGACTTGTT TGGTGTCCGA AGGCGATCTT CCTTTGACCA TTACTTGGAG
TTTTCAAGGG GGTCTTCTCG ATTCAAACAG TGGGGTTATA ACGACAATGA TCGGCAAAAA GGCGAGTTCG CTTCTGATCG
ATTCCGTGTC GGAAGTCCAA AGTGGAAATT ACACTTGCAC GGCTCGGAAT CGTGCCGGTT CTAGTGATTA CACGGCGTCG
TTAGATGTTT ATGGTACTTT TACAAGTTTT ATGTCTGTTG CGTGTTCGAC GGATGATGGG CTTCTGTTTA AAGCTTCCAC
GTGGCTAATA CTTGTTGCAC CATCTCGAGA CTTTGGCAAT GATGCGATCT CAGCTGCACC CTTTCCTTTC TCCTACACCA
CTCCTGAGCT GAGATCTAGG GATTTTCCCG TTCTCTATGT CCCGTGCACT TTTTCTTCTT CTTCTTCTTT AAGTTAACTC
TTTGCATGCA GTTTGTTTCT GTTGTCCTTT CACTTATTTC AGTTACTAAC TGGCACGTAA GGAGTGAAAT AATATTAGGA
AAGTTCAAAT ACAATATTAT TAGTTCCTTC GTGCAGAAGA TAGAAGGTAC TGATTTTTTC GAATAATAAA ATTTCAGTTT
TGAAAAAATC AGTCCTTTAA TATTTTCGGC CAACAAAATT ATTAATGCAT GCCAGAAGCT CAATTTCGGC ACTCAAAATT
AATTGCATGT CATTTGCAAG TTCAAAATGC ATTTTGACTG ACCGAACGCA ATTTTTAGTG CCACCGAGGT GGATATTAGA
GCCAACAGAC AAAGCATTTG CTCAAGGCTC TGATGCCGCT GTTGAATGTA AAGCCGACGG GTTCCCCAGG CCTGTAGTGA
CATGGAAAAG GGCTACTGGT TAGTCCCTCC ACTCAATAGA AATTCTCAAT TACAAATTCT CAATTCTAGG GGTTTCACCC
GGCGATTACA AAGATTTCAA ACCAAACAAT CCCGATATTA AAGTCGAAGA CGGCACTCTT ACTATTAACA ATATCCAGAA
AACAAACGAG GGTTATTATT TATGTGAGGC TGTCAATGGG ATTGGATCAG GATTATCTGC AGTTATTCAA ATCAGTGTTC
AAGGTACTGG ACACAATATT GTAATGGGGA AAAATTAACT GTTGCTTGTA GCTCCCCCAC AGTTTGATAT TAAACTCAGG
AACCAAACCT CCCGCCGTGG AGACCCTGCC GTCCTCCAAT GTGAGGCCAA AGGCGAAAAA CCGATTGGTA TTTTATGGAA
TATCAACAAT AAGCGTTTGG AACCAAAAGG CGACAATAGA TACACGATCC GGGAGGAGAT CCTCGCCAAT GGTGTTCTTT
CCGGCCTCAG TATCAAACGC ACAGAACGCT CCGACTCCGC TCTCTTTACT TGTGTAGCTA CCAACGCCTT CGGCAGTGAC
GATACCAGCA TTAACATGAT TGTGCAAGAA GTACCAGAGG TACCATACGG GCTGAAAGTA TTGGACAAGT CGGGCCGAAG
CGTCCAATTG TCATGGGTTG CACCATTCGA TGGCAACTCC CCGATCACTC GTTACATGAT CGAGTACAAG CAAAGCAAAG
TCAGTTGGGA GGGAAACACC GAACGGGTAC TAGTCCCAGG TGACCAAACT GAAGCTGGGG TTTTCACTCT GCGACCTGCC
ACCACGTATC ACATCCGCAT AGTCGCAGAA AATGAGATTG GGTCAAGTGA ACCCTCTGAG ACTGTGACCA TAATCACGGC
AGAGGAGGTA CCCGGGGGGC CACCAACCAG TATCCGAGTT GAAACAAACG ACCAGCATTC ATTGGTCGTC TACTGGAAGC
CTCCAGCTCG AGAGGAGTGG AACGGCGATA TTTTGGGCTA TTATGTTGGT TACCGCTTGG CTAATTCAGA CAAGCCGTAC
CTTTTCGAAA CCGTCGAATT CGGACGTGAG GAGGGCAAGG AGCACCATTT GAAAATATCG AACTTGAAGA CCTACACCCA
GTACTCTGTG GTGGTCCAAG CGTTTAATAA AGTGGGAGCT GGTCCCTTGT CTGATGATAT CAAGGCGTAT ACGGCTGAGG
GAGTACCTGA ACAACCCCCA GATAAGGCCA CTTGCACCAC CTTGACGGCC CAAACGATTC GCGTTTCGTG GGTGTCGCCG
CCCCTCACGG CCGCCAACGG GGTTATTAAG GGCTACAAAG TCATCTATGG ACCATCAGAC ACTTGGTTTG GTAAGTCATG
TTGGAAAAAA ATCTATGTCG CTCATACATT TTTCCAGATG AGAACACCAA AGACACCAAA ATTACCGCTG CTAGTGAAAC
GATCCTCCAC GGCCTCAAAA AATACACCAA CTACAGCATG GAAGTCCTGG CTTACACTTC CGGGGGTGAC GGTGTTCGCA
CAACCCCGAT TCACTGCCAG ACTGAACAAG ACGGTACTTA TCGGTTTTTA GTGAAAGTTT GCCTAATTTT GTAATTGCAG
TGCCGGAGGC TCCAATCGCC GTTAAGGCTC TAGTAATGTC AACTGATTCG ATTTTGGCCA GTTGGAAGCC CCCAGTTGAA
CCAAATGGCA TTGTCGAGTA CTACACGGTT TACTACAAAC CGGTCTCAAC CGATGACAAA ACCGAGGTCA AGCCAACGTC
TCAAAAAATC GTACCAAACT TGCGCAACCA GAATTTGAGC CACCAAGCGA AAAACCTTGA CAGTAACTTG AAATACGAGT
TTTGGGTTAC CGCAGCTACC ACTATTGGAG AAGGGCAACC GTCGAAGAAA GTTACAGTGT CTCCAAGCGC GAGCGGTAAT
TTTTTCTAGT GGTTTAAGCA CCAAAGGAAT GTTTGGTCAG TTCCAGCCAA AATCGCCTCG TTTGACGATA CCTTCACCAC
GACGTACAAG GAAGACGTGA CTCTCCCTTG CCTCGCCGTT GGGTTGCCAC CACCGGTCAT CACATGGAAA ATCAAGGGGG
TTCAGTTCAC CACAAGCGAC AAAATCAGGC AACAACCAGA CGGGTCACTG TTTATTCGTG ATGTCAGTCG GAATAACGCA
GGGGAGTACT CGTGTCACGT TGAGAATGAC TATGGACAGG ACTCGGTGAC TCACCAGTTG ATTGTCAATG CTCCTCCACA
CGCACCACAA ATTGCTCTCA CTTCAACTAC CACAAATTCG CTCACGTTTA AGTTAAAGCC GCATGAGTCG GATGTTGAGC
CGATCCATGG ATACACTATT CACTACAAGC CAGAGTTTGG CGATTGGGAG ACGGTCCAGA TTGGACCAAC TGTCGAAAAG
TACACTTTGG AGAAGTTGCT GTGTGGGACG CGGTACCAGG TCTACGCCAA GGCGTATAAC AGGTCAGTGA CTTAGTTGGA
ACGATGTGTG GTTTCGTGCC AATTCGGTTT GTAAGACAGA GGCAACAGCG TTATTTTAGC AGAGATTTGT GGTGATACGA
AAGATCAACA TTTGCCTTTT CACCAGATCA TTATAATACG CGTGTGTGAG GATTCAAAAT AGGAGGGTAT AATAAAACAG
TTTATCATGG GGAAAACATT AGTTTATTCA ATAATAAGTT AAAGTAACAG TGTGTAATGT AAATCTAGTT ACATATTAAC
GGAGCCTAGT AACAGACCCA TTACATGGCT CTCAGCGACT TCCTTGAGAA AAGCATCAGA ATAAATAACA CTACACAAAT
AAACTCCAAA TAAAGGTACC CGTCCGTAAA CAACAAATCA AAAATTAACG GTCACTTTAT GAACGCTTTT CCACTGTCAT
CTAGACTTAC TTCTTGCTTA CTCAACACTC CAAGAGTAGC AAAACCGTTG ACAAAAAAAT TTTACATGGA ACCAAATAAT
TAATAAATCT AGTTTAATAA AAACAGGAAC AAAAACTTGA TTTTGTATAA AAACGTGTTT AAATCTTCAG AATTTGAAAA
AACAATGGCG TTTTTGGTAT AATTCGGCTA TTTATTGGCT TACTTTTTAA GACATTTTGC GCATAGTAGA GTTTTTGCTG
AATAAATGTA AATTTTTGAT GATTTTCGAC CAAATTTGGC GATTTTTTGT TCTAGTTTAA TAAAAAAAAT GGAAGAAAAA
CTTGGTTTTT CATCTAAAAA CGTGTTTAAA TCCTCAAAAC TTGAAAGAGT AATGTCATTT TTGACATAAT TCTGTGAATT
ATTGGCTTAC TTTTTAAGAA ATTTTGCGTA ATAATTAAAT TTTTGCTTAG AAAAGGTAAA TTTCTTGTTA TTTTCGACCA
AATTTAGTCT AAGCAGTAAG CGTTAGTCGA TAATTCACGA AACTGAAAGC ATTTTAAAAA ATTTTTCGTG GTGTAGCAAC
TAATTCTTGT GCATGATTTA GTGGTCACGT GACCAAATTG CACCAATCAG ATGTTCGCTT TCACACACTA ACTTTTAACA
ACGCATTAAA TTTTAATTGC GCTTTGATTT TCGTTCCATC TGGTGCTACA TTTTTAGGAA AGAAAGGCAC CAAACAAACG
CAGAATTAAC AGTTTATCAC TGAACACAAA AAACATCGCA AAAGCAACAC GCGTGGGTAG CGTTTCTTTT TTTCCCAGCC
TACTAAGTTC TTCCCCCAAC AACAGATTTT TTATTGCCAA CGTGCAAAAA AATTCCTAAC AACCAACCGC GAATTTTGAA
TTGGAGTTGA TTAGTTTTCA TTTTCCGATC TTGTAATTGC AGCATCGGAA CTGGGGACCC ATCCGACACC CTGAACCCGC
GAACACGTGG CGAAAAGCCC ATAGTGCCCA GCGCTGAGAA GTTCATTGAA GTTTCGTCAA ACAGCATCAC TCTGCACCTC
AGCGCCTGGT CTGACGGTGG TTGTCCCATG CTCTACTTCG TCATCGAGCA CAAAAAGAAG TGAGTTGTAC TTTTGTGCCA
ATTTCCCCAG CTAAGCTTTG TGGTGCTAGG ACTAGTACGG AGTGGATCCA AGTGTCCAAC AACGTGAAGT TGGGCCAGAA
TTTCGTGCTT CTGGACCTGG ACCCGGCCTG CTGGTACCAC CTCCGCGTCA CCGCGCACAA CAACGCCGGC TTCAACGTCG
CCGAATACGA ATTCGCGACG CTCACCGTGA CTGGAGGTAA ATTCGGTTGT TGTCGTTCGT TGTTGTTTTG CTTCCAGTAC
GTGCAATAGT ACCTGAAGAT GAGTTTTCGC GCGGTAACGA GCCTGTTTTT CGTTTCAG**GT ACTATTGCCC CGGCCCGTGA**
AATCCCCAAA GAGGACACTT TTCAAATAAT CCTGGCGAAC CTAAACCTTG TAGTCCCCGT AGTGGCAGCA GTTCTGGTCA
TAATTGTTGC CATTATTGTG ATTTGTGTAT TGCGGGGCAA AGGGAATTAT CATAAAGGTA AAGTTGGTGT TTTCTCTTAT
TTCTAGGTAC AATCGCGCCA ATTAGGGAAT CGCCCGGTGT TAAGATGCTC TTTCCTTGGA TACCCGACTG GGTCGACTTG
AATATTGCCG TTCCCGTTGC CGCAACGGTT GTTGTTGTCG TTGTCGGCAT TGTCGTGATT TGCGTCGCGC TCTCTCGCAG
GGCCAACGGC CCCTTACAGA CGCGCCTCAG AAGCGACTGT ACGTCACAAC ACAAACATTT TACACCAACC AAATATCTAA
GCGCTTGGAG GGACACACGC AAAGCTCGAC TCAATGAACA AATAAAGCGC GCTCGATATG GAATATCCAC CAAAATGTCT
TTATTGCCTG CTTGGCTGTA CAGTCCTGCT TGAAATTATT CCATATTCTG CACTATTTCC AATTTTCTTC TAGCACTCAC
TTCACCTTTT TCGTTTCGAA TTTTTTCCCT ATCAAACTCA CACACTCTGA CACATAAACT GCACATTCTT TCACCTGTTC
TATATTTTTT CGTCCGTTGT CATTTAAAAA GCAACTTCCA ATCTTTTTTT CTTAGATATT TTTCCATTAT TTTTATCATT

robert peuß 1/6/2015 15:31
Comment [9]: Exon 11
120 bp

robert peuß 1/6/2015 15:31
Comment [10]: Start ibeetle RNAi construct (iB_05264)
Lenght: 510 bp
For more details see: http://ibeetle-base.uni-goettingen.de/details/iB_05264

robert peuß 1/6/2015 15:31
Comment [11]: qPCR Primer Forward
see Table S1 for more detail

robert peuß 4/6/2015 13:02
Comment [12]: Dex-12 Primer Forward
See Table S1 for more detail

robert peuß 1/6/2015 15:31
Comment [13]: qPCR Primer Reverse
see Table S1 for more detail

robert peuß 4/6/2015 13:02
Comment [14]: Dex-12 Primer Reverse
See Table S1 for more detail

robert peuß 1/6/2015 15:31
Comment [15]: Exon 12
174 bp

robert peuß 1/6/2015 15:31
Comment [16]: End iBeetle RNAi construct

robert peuß 1/6/2015 15:31
Comment [17]: Exon 13
1059 bp

robert peuß 1/6/2015 15:31
Comment [18]: Exon 14
156 bp

robert peuß 4/6/2015 13:02
Comment [19]: Dex-15 Primer Forward
See Table S1 for more detail

robert peuß 4/6/2015 13:02
Comment [20]: Dex-15 Primer Reverse
See Table S1 for more detail

robert peuß 1/6/2015 15:31
Comment [21]: Exon 15
315 bp

robert peuß 1/6/2015 15:31
Comment [22]: Exon 16
503 bp

robert peuß 1/6/2015 15:31
Comment [23]: Exon 17
177 bp

robert peuß 1/6/2015 15:31
Comment [24]: Exon 18
167 bp

robert peuß 1/6/2015 15:31
Comment [25]: Exon 19
159 bp
Ortholog to exon 17.1 in D mel

TCCTTCATCT TATTCTTGCT CTTTGCTACA ATTATCAAAT CTATCTATCA AAACTGTTCC ACCAACTTGT GCTTTTTTTA
GAACCTTATC TAGGTATTTC CCCCAAGTAA GCTAAGAATA ATACAGGACG TAACGAACCC TTGTGGTTGG TATCTTTAGT
TTTTTGCTTT TCTTCGCCAA AAACAATCGC TCACTTCTCT CTAATTTGTA AATTAATATT TATGAGCCTT GTTATCCGCT
TTTTTCTTCA AACAAGCCAG GACTGGACTC TCGATACGGA ATATAGTATG TTTATGTTAA GATCACTTTG TGGGCCCTCT
GAGTCTTGCA CCCATAGTAG ATCCCGCCAC GCCCTTTTGG AGACACCTTT TATTTTGCAG ACGACGTCGT TTACAACCAG
TCCGTAAACG CCTCTTCGAC TCTGGACAAG CGCCGGCCCG ACTTGCGCGA TGAGCTGGGC TACATTGCGC CCCCTAACCG
CAAACTGCCC CCCGTTCCAG GCTCAAACTA CAACACCTGC GATCGCATCA AGCGAGGTAC CGTCTTAAGT AAGAATCAGC
CATCGCTTCT TATGCATGTC CTGTTAATTT CGAATTTAAT TTTGTGTATA TTCAGTTCAC TGCTGTGCTC ATTCGTCAAC
TTCCCGCACC GGAATTTGAC GAACTGTTTC ATCCTAATCC GCGCTGTTTA TTATTTTTAA CAATTTTGCT GTGTGAGCCG
ACTTCCATTT CATGCGACAT GTGCTTGATT ATTTTAAATG TGCAATAGGA GCTCATTATC GTTCTCATTC TACCTGGGAT
CCGAGACGAC ACTTGTACGA GGAGCTTAGG AGTAGAAGGG GCTCAAACGA GACTGTACAT ACGCACAGAG GTAAAATTTG
ACACTTACAG AATGGTTTGT AAGCTTTCGT GCTTCTGGTA CTAACTACAG TACTTAGATT TGTCATCTGT CTGCGTGTTT
GAGTTACTAA CAGGTTTGAC ACTAACACCA CAAACATGTT TTATACGCTT TTGTCCAGCC ATGGTCATTA ATTAAAGTGT
TTTCGCTTTT TTGTTCTGAA GTTTTTCAAG TTTATGATTG TGTAAAACGT AACCTGGTCT AATAGTTATT CCGTGTACTG
AAGATGGAAG AGATTAAGAA ACATTTTTTC ATAAGTTTAA ATCGTTTATT CATATTTTGG TTGAGTGGAA ATTTTTGACC
CGGCGCATCC ACCATTTTTC TCGATGTTTA ACTATTGTTC ATTTTCGTTG CACACTTTGT ATAAAACATG TTACACTGCG
AAACAACCGA CTAACGCTCT AACATGTAGG CTTATGTCTC CTCCGAACGT AAGTTATCAC TGGCTTTTGT CCGCTTGCTG
CCCATCACCC ACCCATTGAA ACCATCGCAA GCCAACCAAA CAACCATTTT TTAGGCATGG ACGACGAGAT CTGTCCCTAC
GCCACCTTCC ACCTTTTGGG CTTCCGTGAA GAAATGGATC CGAGCAAAGC GATGCAATTC CAGACTTTCC CCCACCCCCA
CTCAGGAACA ATGGGACCAT CGGGGATGAA CACACCCCAT CAGATCCACT CGCGCTCAGG ATCACAGTCG ATGCCGCGCC
AAAATCGGCG ATACGACCGC GTCGGGTCAC AAGGTACGGC CTCTATCCGA ACGTATTGAA ATCTTGCATA CTTACGTAAT
ATTTACTTTA GTTGCTAATA CTTTATAAAT AATTAATAAT TGCCATTAGT ATTTACCAAA ACTTGAAAAG TAAAGTATAC
AGTGCACGAT TAGCCCAGTT AATCGCGACT AGTTCTCCCC TCATAGAAAA AAATATTTAA TACACTGTGC AATTGTAATG
GTTGTGGTTT AGGTAATGGC AGCATCTACT CCCCTGGGCC CGAGTACGAC GACCCGGCGA ACTGCGCGCC CGAAGACGAG
CAGTATGGCT CGCAATATGG GGGCTATGGG GCCCCCTACG ACCAGTATGG CAGCCGGGGG TCGATCGGCC GGCGCTCCTT
GGGGTCTTTG CGGCTCCAGC CGACGAGCAG CAGCCCCGAG CCGCCGCCGC CGCCCCCACG CAACCACGAC CCGTCTTTCA
ACGACTCCAA AGACAGCAAT GAGATTTCGG AGGCCGAGTG TGACCGAGAC CAACTGATCA ACAGTCGCAC CTATGGCGGT
AAGTAGCGCT TAGGTCGGCC TGTTTTCACT AATCTCGCTT CAATTAATTA TTAATTGGCC GTTTGACGAA CGAAATTTTA
AAAACCGATT TATTTTCAGA AAATGAACAG GGTAAGTGAG TTGTTTGACC GTAAAATCTA TTAGGATTAG ATGAGGTTCT
TCAACTCATT CATCACTCAT TGAAATCACT TCATGATTTT TACGTGTGTT GCATGGACTC ATATTGTGTG TTAATCGCAT
GTGGACATTT TTTTCCTTCA TTTTGGCATT GCATACTTTT GTTTCGTTTT GTTTTGTTTA AGCTAGTGAC GTCTCGTGGT
CGTGTAGTAA GTGTGGTTTG TTTTCAGTAA TGAGAGGTAG TTCAAAGGAT GGCATGTCTC ACGAGGAAAT GCGCAAACTG
ATTGAAAGGT CAGTACACAC AGATACAACC CCCTTAAATT CGCAAAAGCC AGCTAAGCCA GTCAAATCGC ACAATTAAAA
AAAATTAAAT AAAAAATTAC GAGCCATTAG TTCAACATTG AAGCTTGGCT ATTATTAGCA ATAATTATTG CTAATAACAA
TTTTGTAAAA ATTAATAAAA ATGGTCACGC GTCAAAGGAT TCGAAAGTAA ATAAGTAATA AAAACGCTAT CCATTTTTGA
CGAAGGTAAA AATACAACCC CTAAATCAGT TTGACAGTTT GTATATTTTC AAAATATGTT TCGAGTTACT TATCTCACAG
ACTGTAAATA AAACTAGAAA TTTTTTTGCA GTATACGAGT ACAAACTTAT TGAAATAAAA ATTGATGTAC TTATTAGGTT
TTTTTTCATA AAGGTTTCTT TTTTTTAAAC ATTTGATAAA TAAATTGTGT TTTGAATGGC CCTGAAGTTT TCTTGAAACA
AATTTTGAGT ATAATTTTAG GCATATTTCA GTTTTTCATG CATATTTTGC TTAAATTTTA AACTTATTTT AGCGCTTCTT
TCCATCATTT TTGGCCTAAA AAATCCGAAC TCTACGAATT TCTTTAATAA ATTCGGGGTT TATAGCACAT TTAAATGCAA
CCAAATTTTA AATTAAAGGA ATTTGTTTAT CTTAATAGAA TTATAATTGA AAAAATTAAA AAATCAATTT GGAATAAGTT
TCAGATGAGG GTTTATATTT TTTTATCTAT TATGCGTGAT TAACTGATCA CGTGACCAAA ATGAACAAAT TTGATTGGTC
TATTCGCGAT GTTAACTTTT AACAGCGCAG TTAACTTTAA TTGAGCTTTC TATAAGTCGG CACTTAGTGT CAAATTTTGT
TGCGTTTGAC TGGCTTATAT CCAAAAATCG TTGAGTTTGT ACTGGAGTGA CTGAAATAAG TATTCTTCAA AAATATTTTA
CCGAAATAAT AACAAAATCG CATTATATTC CGACCACTGA GGGGACAAAA AGTGGGCATG CGACAGTCCC TTCACTGATT
GGGATTTCAA TGTTTAGTAT TTTTTTGTCA CTTCAGTACA ATCAATCGTT GTGATAGCGT TGTGATAATG ATGTTTGTTA
TTTCATGAAT TCCTCAAATA GGAAACTGTA AGTGTGTACT GCAATTAAAC CGCTTTAAGT GTTTTTCTTT TTTCACTTTT
GTGTTTACGT TTGTGCACAC TAATTTCTAC TAACTGTGTT TTCTCTGTCT GACGTACTGT GCTGATGGGT GTGTGAGCAT
TTTGTGTTTG TTTGTTTTTA GAAACGAAAC AGGCCAAGCA AACGGGGGAC TCACAGCCTA CGATACTGTG GCAGTGTAA

robert peuß 1/6/2015 15:31
Comment [26]: Exon 20
168 bp

robert peuß 1/6/2015 15:31
Comment [27]: Exon 21
102 bp

robert peuß 1/6/2015 15:31
Comment [28]: Exon 22
219 bp

robert peuß 1/6/2015 15:31
Comment [29]: Exon 23
306 bp

robert peuß 1/6/2015 15:31
Comment [30]: Exon 24
61 bp

robert peuß 1/6/2015 15:31
Comment [31]: Exon 25
58 bp

References

1. Milutinović B, Stolpe C, Peuß R, Armitage SAO, Kurtz J. The Red Flour Beetle as a Model for Bacterial Oral Infections. *PLoS ONE*. 2013;8(5).

2. Wiegmann BM, Trautwein MD, Kim J-W, Cassel BK, Bertone M, Winterton SL, et al. Single-copy nuclear genes resolve the phylogeny of the holometabolous insects. *BMC Biol*. 2009;7:34-.

3. Misof B, Liu S, Meusemann K, Peters RS, Donath A, Mayer C, et al. Phylogenomics resolves the timing and pattern of insect evolution. *Science*. 2014;346:763-7.

4. Clancy DJ, Kennington WJ. A simple method to achieve consistent larval density in bottle cultures. *Drosophila Information Service*. 2001;84:168-9.

5. Werner T, Liu G, Kang D, Ekengren S, Steiner H, Hultmark D. A family of peptidoglycan recognition proteins in the fruit fly *Drosophila melanogaster*. *P. Natl. Acad. Sci. USA*. 2000;97:13772-7.

6. Lord JC, Hartzer K, Toutges M, Oppert B. Evaluation of quantitative PCR reference genes for gene expression studies in *Tribolium castaneum* after fungal challenge. *J. Microbiol. Meth.* 2010;80(2):219-21.

7. Toutges MJ, Hartzer K, Lord J, Oppert B. Evaluation of reference genes for quantitative polymerase chain reaction across life cycle stages and tissue types of *Tribolium castaneum*. *J.Agricult. Food Chem.* 2010;58:8948-51.

8. Ling D, Salvaterra PM. Robust RT-qPCR data normalization: Validation and selection of internal reference genes during post-experimental data analysis. *PLoS ONE*. 2011;6(3).

9. Ponton F, Chapuis MP, Pernice M, Sword G, Simpson SJ. Evaluation of potential reference genes for reverse transcription-qPCR studies of physiological responses in *Drosophila melanogaster*. *J. Ins. Physiol*. 2011;57(6):840-50.

10. Martins NE, Faria VG, Teixeira L, Magalhães S, Sucena É. Host Adaptation Is Contingent upon the Infection Route Taken by Pathogens. *PLoS Path*. 2013;9(9).

11. Behrens S, Peuß R, Milutinović B, Eggert H, Esser D, Rosenstiel P, et al. Infection routes matter in population-specific responses of the red flour beetle to the entomopathogen *Bacillus thuringiensis*. *BMC Genomics*. 2014;15(1):445-.

12. Zou Z, Evans JD, Lu Z, Zhao P, Williams M, Sumathipala N, et al. Comparative genomic analysis of the *Tribolium* immune system. *Genome Biol*. 2007;8:R177-R.

13. Shrestha S, Kim Y. Activation of immune-associated phospholipase A2 is functionally linked to Toll/Imd signal pathways in the red flour beetle, *Tribolium castaneum*. *Dev. Comp. Immunol.* 2010;34(5):530-7.

14. Yokoi K, Koyama H, Minakuchi C, Tanaka T, Miura K. Antimicrobial peptide gene induction, involvement of Toll and IMD pathways and defense against bacteria in the red flour beetle, *Tribolium castaneum*. *Resul. Immunol*. 2012;2:72-82.

15. Jacobs CGC, Van Der Zee M. Immune competence in insect eggs depends on the extraembryonic serosa. *Dev. Comp. Immunol.* 2013;41(2):263-9.

16. Lemaitre B, Reichhart JM, Hoffmann J. *Drosophila* host defense: differential induction of antimicrobial peptide genes after infection by various classes of microorganisms. *P. Natl. Acad. Sci. USA*. 1997;94(December):14614-9.

17. Leulier F, Parquet C, Pili-Floury S, Ryu J-H, Caroff M, Lee W-J, et al. The *Drosophila* immune system detects bacteria through specific peptidoglycan recognition. *Nature Immunol.* 2003;4(5):478-84.

18. Irving P, Ubeda JM, Doucet D, Troxler L, Lagueux M, Zachary D, et al. New insights into *Drosophila* larval haemocyte functions through genome-wide analysis. *Cell. Microbiol.* 2005;7:335-50.

19. Romeo Y, Lemaitre B. *Drosophila* immunity: methods for monitoring the activity of Toll and Imd signaling pathways. *Meth. Mol. Biol.* 2008;415:379-94.

20. Lemaitre B, Hoffmann J. The host defense of *Drosophila melanogaster*. *Annu. Rev. Immunol*. 2007;25:697-743.

21. Pfaffl MW, Horgan GW, Dempfle L. Relative expression software tool (REST) for group-wise comparison and statistical analysis of relative expression results in real-time PCR. *Nucleic Acids Res*. 2002;30(9):e36-e.

22. Pfaffl MW. A new mathematical model for relative quantification in real-time RT–PCR. *Nucleic Acids Res*. 2001;29(9):e45-e.

23. Sullivan W, Ashburner M, Hawley RS. *Drosophila Protocols: Cold Spring Harb. Laborat. Press;* 2000.

24. Knorr E, Schmidtberg H, Vilcinskas A, Altincicek B. MMPs regulate both development and immunity in the *Tribolium* model insect. *PLoS ONE*. 2009;4(3).

25. Posnien N, Schinko J, Grossmann D, Shippy TD, Konopova B, Bucher G. RNAi in the red flour beetle (*Tribolium*). *Cold Spring Harb. Protoc*. 2009;4(8):1-22.

26. Elbashir SM, Martinez J, Patkaniowska A, Lendeckel W, Tuschl T. Functional anatomy of siRNAs for mediating efficient RNAi in *Drosophila melanogaster* embryo lysate. *EMBO J.* 2001;20(23):6877-88.

27. Roth O, Sadd BM, Schmid-Hempel P, Kurtz J. Strain-specific priming of resistance in the red flour beetle, *Tribolium castaneum*. *Proc R. Sci. B.* 2009;276(1654):145-51.

28. Therneau TM. Coxme: Mixed effects cox models. 2011.

29. Therneau TM. A Package for Survival Analysis in S. 2.37-4 ed2013.

30. Therneau TM, Grambsch PM. Modeling Survival Data: Extending the Cox Model. New York: Springer; 2000.

31. Pinheiro J, Bates D, Debroy S, Sarkar D, Team RDC. nlme: Linear and nonlinear mixed-effects models. 2011.

32. Therneau TM. bdsmatrix: Routines for block diagonal symmetric matrices. 2009.

33. Bates D, Maechler M. Classes and methods for dense and sparse matrices and operations on them using 'LAPACK' and 'SuiteSparse'. 1.2-0 ed2013.

34. Milutinović B, Fritzlar S, Kurtz J. Increased survival in the red flour beetle after oral priming with bacteria-conditioned media. *J. Innate Immun.* 2014;6:306-14.

35. Sokoloff A. The Biology of *Tribolium*: Oxford University Pess, London; 1974.

36. Lewis SM. Multiple mating and repeated copulations: Effects on male reproductive success in red flour beetles. *Anim. Behav.* 2004;67:799-804.

37. Edvardsson M, Arnqvist G. Copulatory courtship and cryptic female choice in red flour beetles *Tribolium castaneum*. *Proc R Soc B*. 2000;267(1443):559-63.

38. Brown SJ, Shippy TD, Miller S, Bolognesi R, Beeman RW, Lorenzen MD, et al. The red flour beetle, *Tribolium castaneum* (Coleoptera): A model for studies of development and pest biology. *Cold Spring Harb. Protoc.* 2009;4(8):1-12.

39. Michalczyk L, Millard L, Martin OY, Lumley aJ, Emerson BC, Chapman T, et al. Inbreeding Promotes Female Promiscuity. *Science*. 2011;333(September):1739-42.

40. Tremmel M, Müller C. Insect personality depends on environmental conditions. *Behav. Ecol.* 2013;24(November):386-92.

41. Semeao A, Campbell JF, Whitworth RJ, Sloderbeck PE. Response of *Tribolium castaneum* and *Tribolium confusum* adults to vertical black shapes and its potential to improve trap capture. *J. Stored Prod. Res*. 2011;47:88-94.

42. Jasrapuria S, Specht C, Kramer KJ, Beeman RW, Muthukrishnan S. Gene families of Cuticular Proteins Analogous to Peritrophins (CPAPs) in *Tribolium castaneum* have diverse functions. *PLoS ONE*. 2012;7(11).

43. Jacobs CGC, Rezende GL, Lamers GEM, van der Zee M. The extraembryonic serosa protects the insect egg against desiccation. *Proc. R. Soc. B.* 2013;280(1764).

44. Flinn PW, Campbell JF. Effects of flour conditioning on cannibalism of *T. castaneum* eggs and pupae. *Environ. Entomol*. 2012;41(50):1501-4.

45. Park T. Cannibalistic predation in populations of flour beetles. The University of Chicago Press. 1965;38(3):289-321.

46. Dönitz J, Schmitt-Engel C, Grossmann D, Gerischer L, Tech M, Schoppmeier M, et al. iBeetle-Base: a database for RNAi phenotypes in the red flour beetle *Tribolium castaneum*. *Nucleic Acids Res*. 2014;43(November 2014):D720-D5.

47. Nilsson T, Fricke C, Arnqvist G. Patterns of divergence in the effects of mating on female reproductive performance in flour beetles. *Evolution*. 2002;56(1):111-20.

48. Watson FL, Püttmann-Holgado R, Thomas F, Lamar DL, Hughes M, Kondo M, et al. Extensive diversity of Ig-superfamily proteins in the immune system of insects. *Science*. 2005;309:1874-8.

49. Lee C, Kim N, Roy M, Graveley BR. Massive expansions of *Dscam* splicing diversity via staggered homologous recombination during arthropod evolution. *RNA*. 2010;16:91-105.

50. Armitage SAO, Freiburg RY, Kurtz J, Bravo IG. The evolution of *Dscam* genes across the arthropods. *BMC Evol. Biol.* 2012;12(1):53-.
